# Pervasive non-triplet alternative splicing drives functional isoform diversity

**DOI:** 10.1038/s41467-026-71615-5

**Published:** 2026-04-10

**Authors:** Shameerudeen Athavudeen, Neethu Issac, Adam Norris

**Affiliations:** 1 Department of Biochemistry, University of California, Riverside, CA USA; 2 Center for RNA Biology and Medicine, University of California, Riverside, CA USA; 3 Department of Immunology and Theranostics, Arthur Riggs Diabetes and Metabolism Research Institute, Beckman Research Institute of City of Hope, Duarte, CA USA; 4 Irell & Manella Graduate School of Biological Sciences, City of Hope, National Medical Center, Duarte, CA USA

**Keywords:** Alternative splicing, Transcriptomics, Caenorhabditis elegans

## Abstract

Alternative mRNA splicing is an important mechanism for regulating gene expression and generating transcriptomic diversity. Most cases of alternative splicing studied to date are triplet, meaning that both isoforms retain the same translational reading frame. Indeed, non-triplet alternative splicing is sometimes considered evidence of splicing errors or noise. Nevertheless, some examples of functionally important non-triplet alternative splicing exist. We set out to determine the global prevalence, regulation, and function of non-triplet alternative splicing in vivo in *C. elegans*. Here we use RNA-Seq and bioinformatic analysis of wild-type and NMD-deficient mutants to categorize the molecular consequences of non-triplet alternative splicing into three classes: NMD-sensitive isoforms, alternative C-terminal length isoforms, and dual-coding isoforms. We identify hundreds of non-triplet alternative splicing events across these three categories. Genetic and molecular analyses reveal cases of developmental regulation, splicing factor autoregulation, cell-specific splicing, and physiologically important isoform-specific function. Analysis of human transcriptomes reveals broadly similar patterns and distributions of non-triplet alternative splicing. Together these experiments demonstrate the importance of non-triplets, a large but underappreciated class of alternative splicing, for regulating gene expression and generating protein-coding diversity.

## Abstract (web summary)

Most alternative splicing preserves the reading frame, leaving the role of non‑triplet events unclear. Here, the authors identify hundreds of non‑triplet splicing events in C. elegans and humans, showing they generate NMD‑sensitive, C‑terminal‑altered, and dual‑coding isoforms with regulated and functional roles.

## INTRODUCTION

Alternative mRNA splicing can be used by a cell to regulate gene expression and to generate molecular diversity^1–3^. Great strides have been made in understanding the mechanisms of splicing and the regulation of alternative splicing. For most genes, however, the molecular or biological significance of alternatively-spliced isoforms remains unknown^4,5^.

Most commonly studied alternative splicing events are triplet, meaning the difference in length between the two splicing choices is divisible by three nucleotides, and thus the translational reading frame of the mRNA is preserved. Indeed, triplicity is sometimes considered a criterion for functional alternative splicing^6–8^ and non-triplicity considered evidence of non-functional splicing noise or errors^9,10^. This is based on the notion that non-triplet alternative splicing often leads to premature stop codons that either trigger Nonsense Mediated Decay (NMD)^11,12^ or result in truncated proteins^13,14^. Nevertheless, over half of alternative splicing events observed in humans cells are non-triplet^15^. And examples of functionally-important non-triplet isoforms, both NMD-sensitive and NMD-insensitive, hint that non-triplet alternative splicing is more important than currently suspected^16–21^.

In one scenario, non-triplet alternative splicing coupled to NMD could represent a functional, regulated process used to control gene expression levels. Such regulation is well-established for a class of exons known as poison exons. These exons harbor in-frame stop codons, and their inclusion downregulates the host gene. Strikingly, many poison exons are “ultra-conserved,” suggesting the importance of this gene regulatory mechanism^22,23^. Non-triplet alternative exons could represent an additional means of poison generation by causing a premature stop codon downstream in the altered reading frame. Indeed, such poison non-triplets have been identified with important functions^16,24–26^. Non-triplet alternative splicing could also generate “essential exons” where the exon-included version is productive, and the exon-skipped version is poisonous. Likewise, poison alternative 5’ or 3’ splice sites could also be generated by non-triplet splicing. Together, these phenomena could greatly expand the post-transcriptional gene regulatory options available to a cell.

In a second scenario, NMD-insensitive non-triplet alternative splicing could generate alternative protein isoforms due to alternative mRNA reading frames. If one of the two reading frames encodes a stop codon immediately downstream of the splicing event, then the primary effect of such alternative splicing would be to generate proteins with different C-terminal lengths. If both reading frames lack stop codons immediately downstream, then a dual-coding region would be produced, in which the nucleotide sequence is identical between isoforms, but the encoded amino acids differ due to altered reading frames. Functional examples of both of these types of NMD-insensitive non-triplet alternative splicing exist^17,18,27,28^. These examples demonstrate that non-triplet alternative splicing, though relatively understudied, is clearly used in animal transcriptomes to regulate gene expression and generate protein-coding variants.

To obtain a global picture of non-triplet alternative splicing in vivo and to interrogate its regulation and functional consequences, we performed RNA-Seq on wild-type and NMD-deficient *C. elegans*, then classified non-triplet alternative splicing into three possible functional outcomes: (i) NMD degradation, (ii) alternative C-terminal protein length, and (iii) dual-coding alternative reading frames. We identified hundreds of non-triplet alternative splicing events across these three categories, including individual cases of cell-specific, developmentally regulated, and auto-regulatory splicing events. Endogenous genetic perturbations revealed functional requirements for both alternatively spliced isoforms in multiple genes with non-triplet alternative exons. Together these results demonstrate the importance of non-triplet alternative splicing, a large but underappreciated class of regulated splicing that is leveraged for controlling gene expression levels and for generating protein-coding diversity.

## RESULTS

### Abundant non-triplet alternative splicing in *C. elegans*

**Fig. 1:**
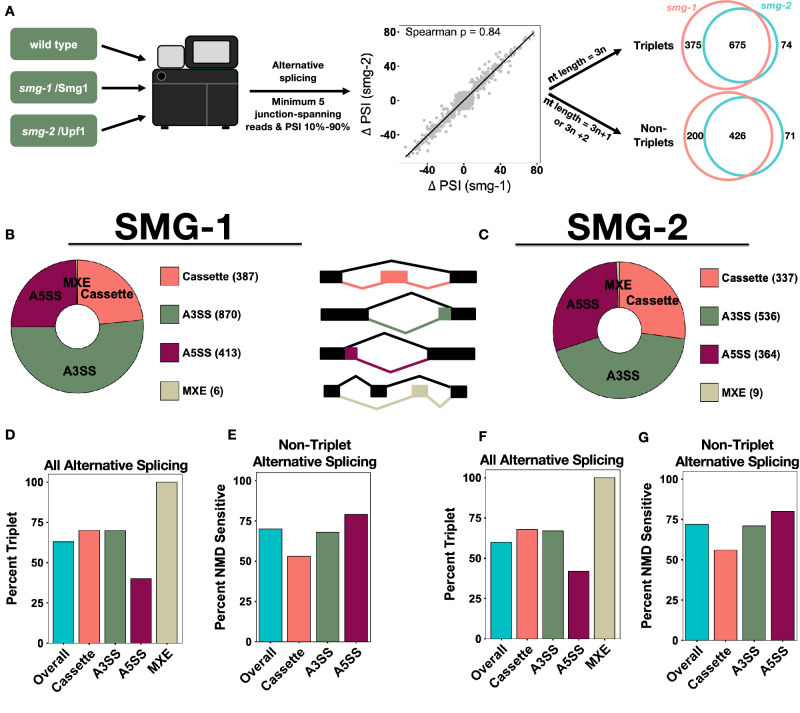
Identification of abundant non-triplet alternative splicing. **A** Schematic overview of experimental workflow used to identify triplet and non-triplet alternative splicing events. Correlation plot compares the magnitude of splicing change (ΔPSI) for splicing events (*n* = 1101) detected in both *smg-1* and *smg-2*. Venn diagram indicates the number of shared triplet (reading frame preserving) and non-triplet (reading frame disrupting) alternative splicing events in *smg-1* and *smg-2* versus wild-type comparisons. **B**, **C** Pie chart quantifying the distribution of total alternative splicing identified in four categories: cassette exons, Alternative 3′ splice sites (A3SS), alternative 5′ splice sites (A5SS), and mutually exclusive exons (MXE) in *smg-1* (**B**) and *smg-2* (C). **D**–**G** Triplicity and NMD attributes of identified alternative splicing events. Histogram displaying the proportion of all alternative splicing events that are triplet in nature in *smg-1* (D) and *smg-2* (F). For MXE, the difference in length between the alternative exons are considered for triplicity. (E-G) Histogram quantifying percentage of non-triplet alternative splicing events that are NMD-sensitive: >10% |ΔPSI| in *smg-1* (**E**) and *smg-2* (**G**).

We set out to systematically identify non-triplet alternative splicing across the transcriptome of *C. elegans*. We aimed to globally identify non-triplet cassette exons, as well as lesser-studied types of non-triplet splicing such as alternative 5’ or 3’ splice sites (Fig. 1A). To ensure inclusion of non-triplet alternative splicing events that might be produced in substantial quantities but efficiently degraded by NMD, we performed RNA-Seq in both wild-type and NMD-deficient conditions. Conveniently, in *C. elegans*, NMD mutants are not lethal, as they are in most other organisms, making it a powerful model organism to study non-triplet alternative splicing at an organismal level^29–32^. We used two different NMD mutants for this analysis: *smg-1/SMG1* and *smg-2/UPF1*^33,34^. RNA-Seq was performed in triplicate for each genotype. Splicing was measured by JUM^35^ and gene expression by DESeq2^36^.

We first identified all alternative splicing events transcriptome-wide, both triplet and non-triplet. We set conservative thresholds to distinguish between constitutive and alternative splicing events, requiring Percent Spliced In (PSI) values between 10%–90% to qualify as alternatively spliced (See Methods). This analysis reveals splicing changes to be very similar between *smg-1* and *smg-2* mutants (Fig. 1A), and to be independent of the bioinformatic pipeline used (Fig S1A–D).

Our analysis yielded nearly 2000 alternative splicing events, with the most common types being alternative 5’ and alternative 3’ splice sites (Fig. 1B, C). Over a third of these cases (37% in *smg-1*, 40% in *smg-2*), are non-triplet (Fig. 1D–F). The alternative splicing type most enriched for non-triplets is alternative 5’ splice sites ( ~ 60% are non-triplet), while the splicing type most enriched for triplets is mutually exclusive exons, which are 100% triplet (reading frame preserving) (Fig. 1D–F). This is in agreement with a previous report in *C. elegans* identifying only frame-preserving mutually exclusive splicing events^37^. We thus identified a substantial proportion of alternative splicing events to be non-triplets in the *C. elegans* transcriptome (Fig. 1B–G).

### Three classes of molecular outcomes for non-triplet alternative splicing

**Fig. 2:**
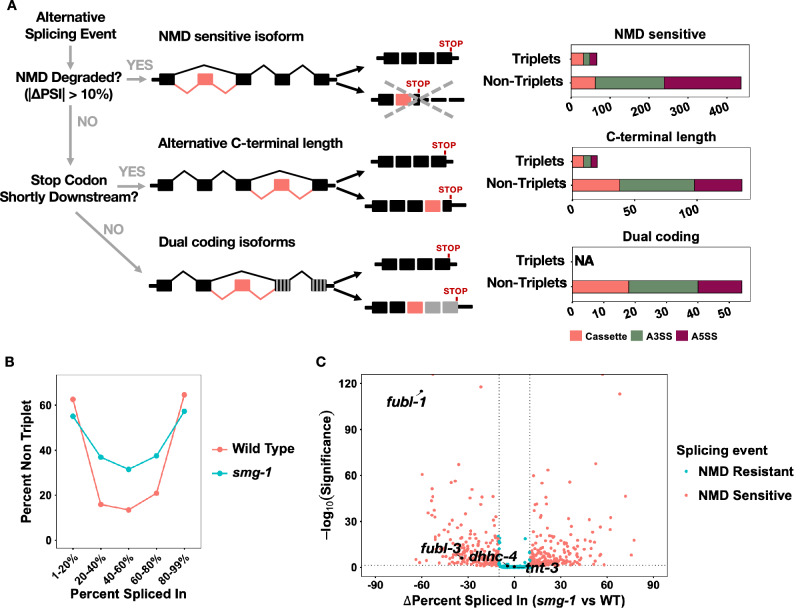
Characterization of functional molecular consequences of non-triplet alternative splicing. **A** Schematic illustrating the potential functional molecular outcomes of alternative splicing, accompanied by a stacked histogram displaying the proportion of each outcome generated by triplet and non-triplet alternative splicing in three categories: cassette, A3SS and A5SS (*smg-1* comparison data). NMD-resistant alternative splicing events (defined as |ΔPSI | <10%) are classified as either alternative C-terminal length or dual coding based on the number of nucleotides recoded (60-nt minimum to be classified as dual coding). Only non-triplet alternative splicing can result in dual coding isoforms. X-axes represent the number of identified alternative splicing events in each category. **B** Fraction of non-triplet splicing events across PSI bins in wild-type and *smg-1*. **C** Volcano plot of non-triplet alternatively spliced genes between wild-type and *smg-1*, Negative and positive change in PSI values represent exon skipping and inclusion in *smg-1* relative to wild type respectively, color coding is based on the NMD sensitivity of the splicing event.

We categorized the non-triplet alternative splicing events into three molecularly distinct outcomes: The first and most abundant class (over 50% of non-triplets, Figs. 1E, 1G) is NMD-sensitive alternative splicing, which we define as splicing events that significantly change in NMD mutants (ΔPSI > 10%, FDR p < 0.05). We find 436 such NMD-sensitive non-triplets (Fig. 2A for *smg-1*, see Fig S2A for *smg-2* data). This molecular outcome is schematized in Fig. 2A, upper row.

The second-most abundant category is alternative C-terminal length isoforms (136 identified, Fig. 2A, and S2A). In these cases, alternative splicing causes a stop codon in one of the two reading frames shortly downstream of the splicing event, causing this isoform to have a shorter coding sequence and a correspondingly longer 3’ UTR (Fig. 2A, middle row).

Finally, dual coding isoforms occur when both reading frames lack stop codons for a substantial length downstream of the splicing event, resulting in a nucleotide sequence that encodes two different polypeptides in two different reading frames (Fig. 2A, bottom row). We set a threshold of 60 recoded nucleotides to qualify as dual coding, reasoning that this is a sufficient number of codons to encode a small protein domain^38,39^. We find 54 such dual coding events (Fig. 2A, and S2A). Note that the threshold set to qualify as dual coding will affect the relative distribution between alternative C-terminal isoforms and dual-coding isoforms (Fig S2B, C).

We tested whether these alternative splicing events shows differential evolutionary trends, but found similar conservation scores between triplet and non-triplet events, and among the three molecular outcome categories (Fig S2D). Taken together, our analysis identifies substantial quantities of all three molecular consequences of non-triplet alternative splicing (Fig. 2A, Supplementary Data 1-4). In the following sections, we proceed to explore the regulatory and functional implications of each outcome in detail, beginning with NMD-sensitive isoforms.

### Steady-state measurements underestimate the amount of non-triplet alternative splicing

Our NMD-deficient RNA-Seq data allowed us to ask whether levels of de novo non-triplet alternative splicing (prior to NMD degradation) might be even higher than those observed at steady state (with active NMD degradation). If this is the case, we might expect to observe a global shift in non-triplet inclusion levels from extreme inclusion values (*i.e*. PSI 1-20% and 80-99%) in wild-type animals to more moderate values (*e.g*., 40–60%) in NMD mutants. This would indicate splicing events where both isoforms are spliced in equal amounts, but one is strongly degraded by NMD.

Indeed, binning alternative splicing by PSI values bears out this notion. In wild-type animals, non-triplets are low-abundance in the middle PSI bins, but their abundance is doubled in NMD mutants (Fig. 2B, and S2E). This indicates that non-triplet alternative splicing is more common than steady-state measurements suggest. Substantial amounts of non-triplet alternative splicing occur, only for the NMD-sensitive substrate to be rapidly eliminated (Fig. 2B, and Fig S2E). These effects can be very strong, with ΔPSIs near 90% between WT and *smg-1* (Fig. 2C, and Fig S2F). This indicates cases where the splicing machinery selects isoform A nearly 100% of the time, but this isoform is so efficiently degraded by NMD that isoform B is detected nearly 100% of the time at steady state.

### Non-triplet alternative splicing expands the reach of poison and essential exons

**Fig. 3:**
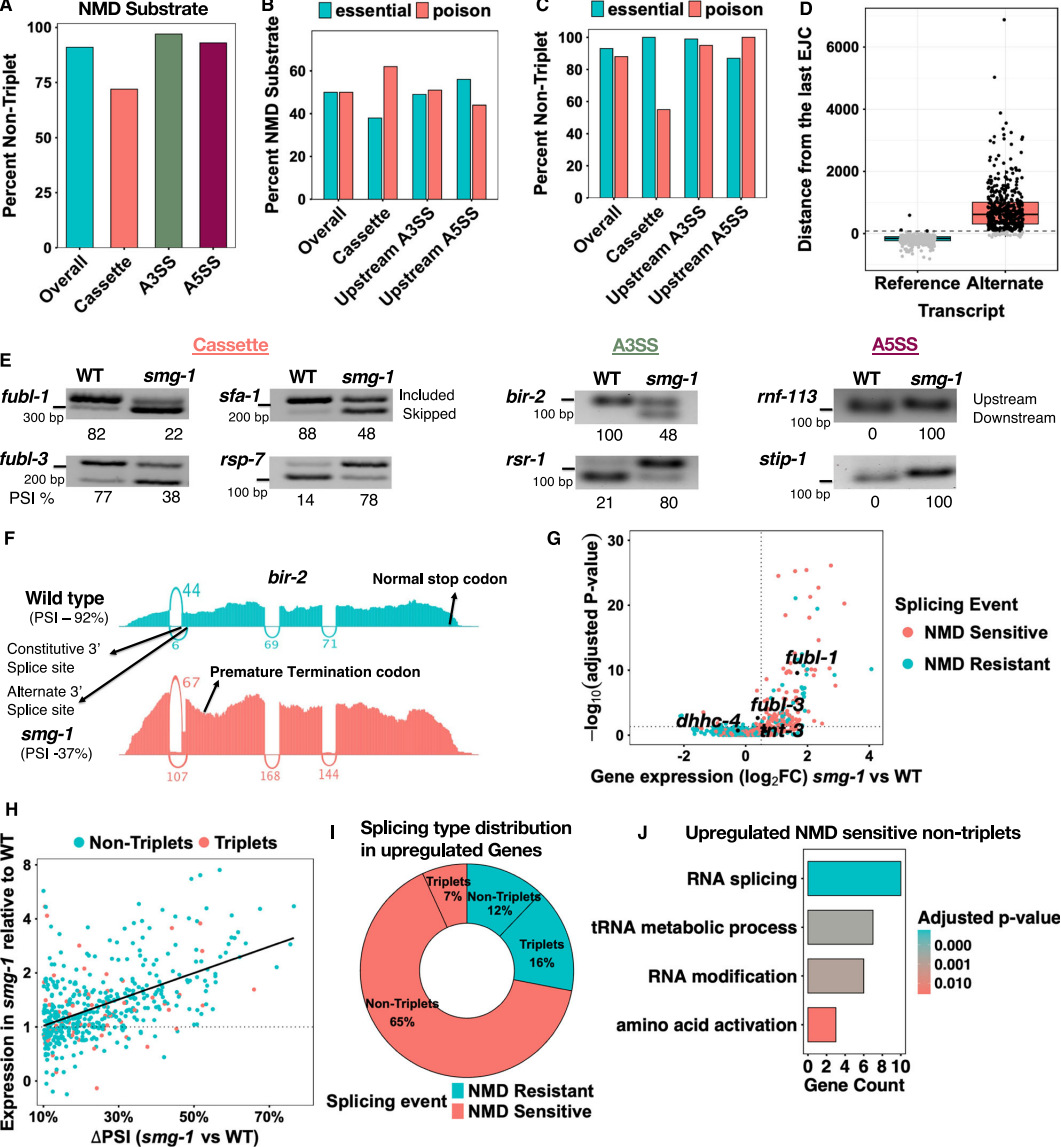
Non-triplet alternative splicing produces abundant poison and essential splice sites. **A** Histogram showing proportion of NMD-sensitive splicing events that are non-triplet in *smg-1* comparisons. **B** Histogram quantifying the contribution of essential versus poison categories in generating NMD-sensitive alternative splicing. **C** Proportion of essential and poison splicing events that are non-triplet. **D** Box plot depicting distance between stop codon and last Exon Junction Complex (EJC) for the reference and alternate transcript for NMD sensitive non-triplets (*n* = 436 events), gray = cases where distance is less than 55 nucleotides, dashed line = 80 nt upstream of the final exon boundary, corresponding to the canonical ~55 nt threshold from the last exon–junction complex (EJCs are deposited ~25 nt upstream of exon–exon junctions). **E**RT-PCR validation of NMD-sensitive isoforms with examples from cassette exons (*fubl-1, fubl-3, sfa-1 and rsp-7*), alternative 3’ (*bir-1, rsr-1*), and alternative 5’ (*rnf-113, stip-1*) splice sites. **F** Sashimi plot of *bir-2* showing alternative 3’ splice in wild type and *smg-1*. **G** Volcano plot of differential gene expression for alternatively-spliced genes (from Fig. 1A), color coding is based on NMD-sensitivity of the splicing events, genes with an adjusted *p* value < 0.05 and log₂ fold change > 0.5 were considered significantly upregulated, analysis was performed using DESeq2 with a two-sided Wald test, and p-values were adjusted using the Benjamini Hochberg false discovery rate (FDR) method. **H** Scatter plot correlation between change in PSI values and gene expression in *smg-1* relative to wild type for NMD-sensitive alternative splicing. **I** Distribution of alternative splicing types for significantly-upregulated genes identified in (**G**). **J** Gene enrichment for non-triplet upregulated NMD sensitive splicing identified in (**I**), statistics were calculated using hypergeometric test (equivalent to a one-sided Fisher’s exact test), Benjamini Hochberg correction was applied to control the false discovery rate (FDR) for multiple comparisons, adjusted *p* values < 0.05 were considered significant.

Alternative splicing coupled to NMD has been well-studied in the context of poison exons – cassette exons that encode an in-frame premature stop codon and are often triplet^40,41^. We find 24 such triplet poison exons in *C. elegans* (Fig. 3A, and S2A), some of which have been previously described^23,42^. However, we find much more NMD-coupled splicing arising from non-triplets (62 cassettes, 177 A3SS, 197 A5SS), suggesting a much broader landscape of splicing/NMD gene regulation than previously anticipated (Fig. 3A, and S2A).

We classified NMD-coupled splicing as either poison or essential based on whether inclusion or exclusion generates NMD substrates. For example, cassette exons whose inclusion causes NMD are poison, while those whose skipping causes NMD are essential. We find that poison cassette exons are slightly more abundant than essential exons (Fig. 3B). For 5’ and 3’ splice sites, we asked whether upstream or downstream splice sites are preferentially poison or essential but observed no strong bias (Fig. 3B, Fig S2B). Next, we asked what proportion of NMD-coupled splicing is contributed by triplets versus non-triplets and found that the vast majority of essential and poison alternative splicing is non-triplet (Fig. 3C, and Fig S2C). The only exception is cassette poison exons, where triplets and non-triplets are equally represented. Together these results indicate that non-triplet frame disruption is a substantially more common mechanism than triplet in-frame stop codons for generating NMD, thus greatly expanding the reach of poison and essential alternative splicing (Fig. 3C, and S3C).

We next asked whether the NMD sensitivity resulting from alternative splicing of non-triplets conforms to current models of NMD-sensing mechanisms, namely the presence of a stop codon 55 nucleotides upstream of an exon junction complex (EJC)^43^, and/or an unusually long 3’ UTR^44–48^. In 91% of cases, the premature termination codon in the alternative transcript is located more than 55 nucleotides upstream of the last EJC, consistent with the EJC-dependent model of NMD (Fig. 3D, and S3D). For the remaining 9% of cases, many possess long 3’ UTRs, consistent with the long 3’ UTR model of NMD^49^, although 3′ UTRs as short as 132 nucleotides also appear (Fig S3E, F). This highlights a complex interplay between EJC positioning, 3′ UTR length, and NMD, with some details yet to be elucidated. We further validated a handful of NMD-sensitive alternative splicing events by RT-PCR (Fig. 3E). PSI values calculated from gel densitometry closely align with the RNA-Seq data. For example, in wild-type worms, the non-triplet exon 3 of *rsp-7* is predominantly skipped (PSI = 14%). Inclusion of this exon disrupts the reading frame, introduces a premature termination codon, and subjects the transcript to NMD. However, in *smg-1* mutants, the unproductive isoform (exon 3 included) becomes the major isoform (78%, Fig. 3E). In another example, wild-type animals predominantly use the upstream 3′ splice site (productive isoform) in the *bir-2* gene, while *smg-1* mutants display equal usage of both upstream and downstream 3′ splice sites (Fig. 3F). Selection of the downstream alternative 3′ splice site renders the transcript NMD sensitive (Fig. 3F).

We next investigated the effect of NMD-sensitive non-triplet splicing on gene expression. Many of the most highly upregulated genes in NMD mutants contain NMD-sensitive splicing events (Fig. 3G, and S3G). The magnitude of alternative splicing change (ΔPSI) is correlated with the magnitude of gene expression change (Spearman’s coefficient = 0.48) for NMD-sensitive alternative splicing (Fig. 3H, and S3H), but not for NMD-resistant alternative splicing (Fig S3I, J). Genes with upregulated expression in NMD mutants are highly enriched for encoding NMD-sensitive non-triplet splicing events (Fig. 3I, and S2K–M). These results indicate that non-triplet alternative splicing coupled with NMD plays a major role in regulating gene expression.

Gene ontology analysis performed on genes encoding NMD-sensitive non-triplets reveals categories related to mRNA binding and splicing (Fig. 3J, S3N). This parallels previous reports of poison cassette exons enriched in splicing factor genes^23,40^, indicating that non-triplet poison/essential splicing affects similar gene networks to those of traditional poison cassette exons.

### Non-triplet essential exons mediate autoregulation of FUBL-1 and FUBL-3 RNA binding proteins

**Fig. 4:**
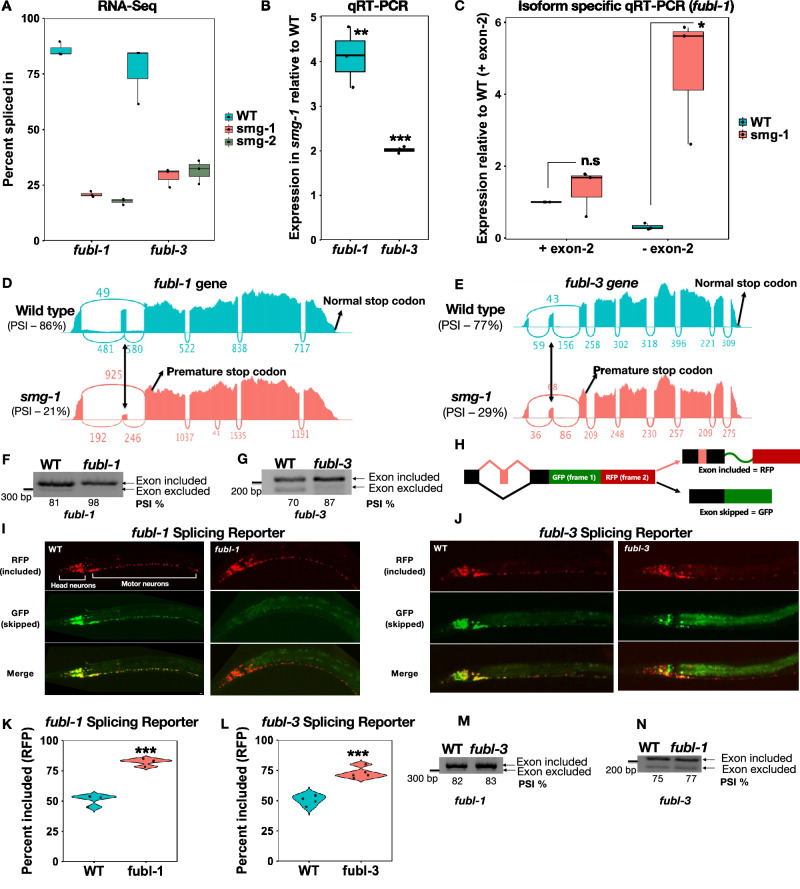
Non-triplet essential exons mediate autoregulation of FUBL-1 and FUBL-3 RNA binding proteins. **A** Percent spliced in values for *fubl-1* and *fubl-3* in wild type, *smg-1* and *smg-2* RNA-Seq data (*n* = 3 biological replicates). **B** qRT-PCR confirmation of upregulated *fubl-1* and *fubl-3* transcripts in *smg-1* relative to wild-type., WT vs fubl-1 (*p* = 0.004869) and WT vs fubl-3 (*p* = 0.0006551) (**C**) Isoform specific qRT-PCR of the *fubl-1* gene, +exon-2 is the productive isoform and -exon-2 is the NMD substrate, expression is relative to the productive +exon-2 isoform, WT vs smg-1 +exon-2 (*p* = 0.99) and -exon-2 (*p* = 0.013). Data points in B and C represent individual biological replicates (*n* = 3). **D**, **E** Sashimi plot showing differential usage of a non-triplet exon 2 in *fubl-1* (**D**) and *fubl-3* (**E**). **F**, **G** RT-PCR confirming the absence of the exon-skipped isoform in the *fubl-1* (**F**) and *fubl-3* (**G**) loss-of-function mutants. **H** Schematic showing the design of the minigene two color splicing reporter plasmid construct. A reading-frame shift due to inclusion or skipping of a frame-shifting alternative exon causes GFP to be translated in frame followed by a stop codon when skipped, while GFP is read out-of-frame (and without stop codons) followed by RFP being read in frame when included. **I**, **J** Fluorescence images of splicing reporters expressed under the control of the neuronal *rgef-1* promoter, RFP = non-triplet exon inclusion and GFP = exon skipping. Scale bar = 20 μm (**I**) and *fubl-3*, scale bar = 50 μm (**J**) loss of function mutant worms. (**K**, **L**) Quantification of the RFP/GFP fluorescence intensities, the ratio is expressed as percent spliced in values (*Y*-axis) in wild-type and *fubl-1* (*n* = 4 worms, *p* = 5.67 × 10⁻⁵) (K) and *fubl-3* (*n* = 5 worms, *p* = 3.44 × 10⁻⁵) (**L**). (**M**, **N**) RT-PCR confirming the absence of cross regulation in *fubl-3* (**M**) and *fubl-1* (**N**) loss of function mutants. For Figure **A**–**C**, the center line in the box plots show the median, with the bounds of the box representing the 25th and 75th percentiles (interquartile range). Whiskers extend to the minimum and maximum values within 1.5 × the interquartile range, points beyond the whiskers represent outliers. Student paired two-sided t-test for 4B, one way ANOVA for 4 C and unpaired two-sided t-test for 4 K&L were performed. Significance levels: *p  <  0.05, **p  <  0.01, ***p  <  0.001.

We next explored the molecular and physiological implications of NMD-sensitive non-triplet splicing. Two of the top NMD-sensitive splicing events from Fig. 1E are in the gene *fubl-1* and its paralog *fubl-3*. Both genes harbor KH domains involved in RNA recognition and are homologous to human FUBP1 and FUBP3. In contrast to poison exons, which have been extensively studied for their roles in regulating transcript abundance, both *fubl-1* and *fubl-3* encode alternatively spliced essential exons, meaning that when the exon is skipped, the transcript encodes a premature stop codon and is subject to NMD. In agreement with our RNA-Seq results (Fig. 4A), qRT-PCR on *fubl-1* and *fubl-3* reveals a strong increase in transcript levels in NMD-defective mutants (Fig. 4B). Isoform-specific qRT-PCR on *fubl-1* reveals that these changes are specifically due to stabilization of the unproductive NMD sensitive isoform, while the levels of the productive isoform remain unchanged (Fig. 4C).

Under wild-type conditions, exon 2 of *fubl-1* is predominantly included (83%). Skipping this non-triplet exon disrupts the reading frame and introduces a premature stop codon (Fig. 4D). In NMD-deficient worms, the inclusion level drops to 22% (Fig. 4D), making the unproductive isoform predominant. Likewise, skipping of the essential non-triplet exon 2 in *fubl-3* also leads to the incorporation of a premature stop codon resulting in NMD sensitivity (Fig. 4E). Thus, for both *fubl-1 and fubl-3*, the splicing machinery predominantly selects the unproductive (NMD substrate) isoforms that skip the essential exon. However, due to their active degradation by NMD, the productive isoforms become the predominant species at steady state conditions.

Many RNA binding proteins are known to autoregulate poison exons in their own transcripts by alternative splicing coupled to NMD^40,50,51^. We considered whether analogous regulation of essential exons might be used by *fubl-1* and *fubl-3* for autoregulation. To test this, we generated loss-of-function *fubl-1* and *fubl-3* mutants and assessed splice isoform usage via RT-PCR. Both *fubl-1* and *fubl-3* mutants display a marked reduction in the unproductive isoform (Fig. 4F, G), while the overall gene expression levels in their respective loss of function are not significantly different from wild type (Fig. S4A, B). This suggests that both RNA binding proteins inhibit inclusion of their own essential exon.

To distinguish between autoregulation via *trans* activity of FUBL-1/3 RNA binding versus potential *cis*-acting effects of the *fubl-1/3* nonsense mutations, we constructed transgenic minigene two-color splicing reporters for exon 2 of *fubl-1* and *fubl-3* (Fig. 4H–J). We expressed these transgenes in neurons, where both genes are highly expressed^52^. The reporter is designed to be translated in-frame with RFP when the exon is included and with GFP when the exon is excluded^53,54^ (Fig. 4H). In wild-type worms, both RFP and GFP signals are observed. No strong signature of unique cell-specific splicing patterns is apparent, as most neurons robustly express both GFP and RFP. In contrast, *fubl-1* and *fubl-3* mutants display a striking reduction in GFP signal, corresponding to a decrease in the exon-skipped (unproductive) isoform (Fig. 4I–L). This indicates that FUBL-1 and FUBL-3 RNA binding proteins autoregulate their expression in trans by alternative splicing of their essential exon, such that low levels of the RNA binding protein led to increased productive splicing, and vice versa.

Because related splicing factors often cross-regulate each other^55,56^, we next asked whether FUBL-1 and FUBL-3 influence each other’s splicing, but found no evidence of such cross-regulation. Neither *fubl-1* nor *fubl-3* mutants influence each other’s essential exon splicing (Fig. 4M, N). Our results thus support the notion that autoregulatory alternative splicing of essential exons, much like the known role for alternative splicing of poison exons^57^, is essential to maintain splicing factor homeostasis. This finding, coupled with the discovery of similarly large numbers of essential and poison 5’ and 3’ alternative splice sites (Fig. 3B, and S3B), greatly expands the known universe of NMD substrates available for alternative splicing-mediated regulation of transcript levels.

### Autoregulation of *fubl-1* is essential for normal development

**Fig. 5:**
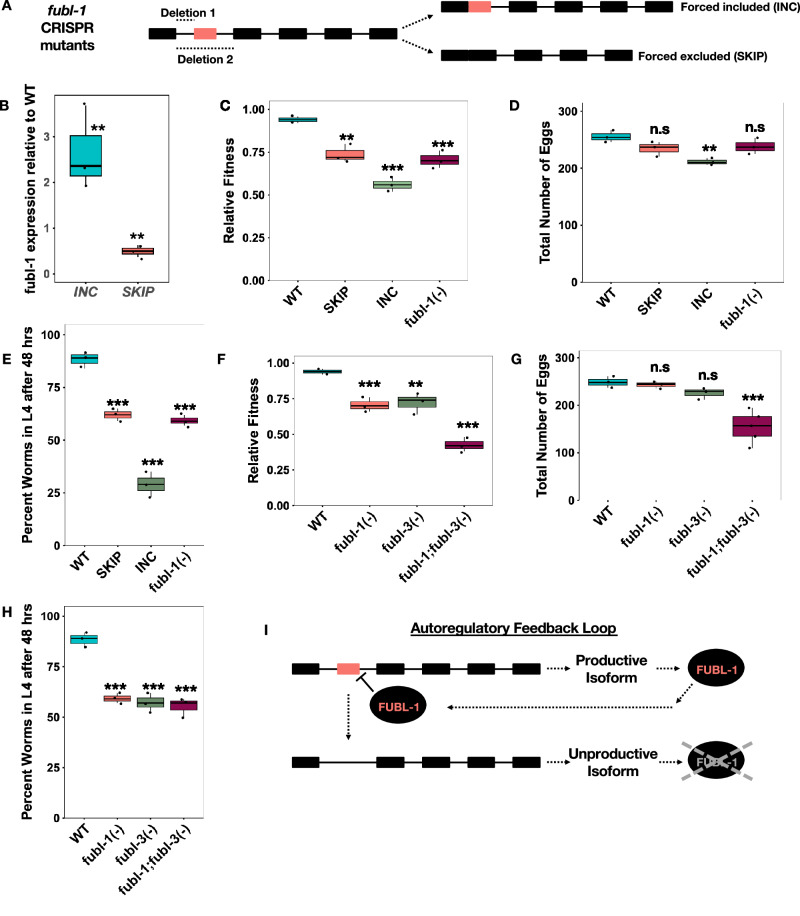
*fubl-1* autoregulation is essential for proper development. **A** Schematic showing the generation of *fubl-1* splice isoform CRISPR mutants. “Forced inclusion” (INC) = intron #1 deletion, and “Forced skipping” (SKIP) = deletion of the non-triplet exon 2 and flanking introns, thus fusing exons 1 and 3 together. **B** qRT-PCR showing changes in *fubl-1* gene expression in the CRISPR mutants relative to wild-type, *n* = 3 biological replicates, WT vs INC (*p* = 0.00179) and WT vs SKIP (*p* = 0.00672). **C** Relative fitness assay (*n* = 3 populations per genotype), each point represents one independent experiment, WT vs INC (*p* = 1.09 × 10⁻⁵), WT vs SKIP (*p* = 0.00250) and WT vs fubl-1(-) (*p* = 0.000795). **D** Brood size, *n* = 3 P0 worms, WT vs INC (*p* = 0.00625), WT vs SKIP (*p* = 0.180) and WT vs fubl-1(-) (*p* = 0.316), points represent individual P0s. **E** Developmental timing assay showing percentage of worms 48 hours after hatching (*n* = 3 plates per genotype), each point represents one independent experiment. WT vs INC (*p* = 1.19 × 10⁻⁸), WT vs SKIP (*p* = 8.02 × 10⁻⁵) and WT vs fubl-1(-) (*p* = 3.04 × 10⁻⁵). **F** Relative fitness, *n* = 3 populations per genotype, WT vs fubl-1(-) (*p* = 0.000795), WT vs fubl-3(-) (*p* = 0.00125) and WT vs fubl-1;fubl-3(-) (*p *= 5.30 × 10⁻⁷). **G** Brood size, *n *= 4 P0s for fubl-1; fubl-3, *n* = 3 for all other genotypes, WT vs fubl-1(-) (*p* = 0.989), WT vs fubl-3(-) (*p* = 0.607) and WT vs fubl-1;fubl-3(-) (*p* = 0.000985), **H** Developmental timing assay (*n* = 3 populations per genotype) for *fubl-1*, *fubl-3* single and double loss-of-function mutants, WT vs fubl-1(-) (*p* = 3.04 × 10, WT vs fubl-3(-) (*p* = 1.53 × 10⁻⁵), and WT vs fubl-1;fubl-3(-) (*p* = 7.92 × 10⁻⁶). For Figure B-H one way ANOVA was performed, the center line in the box plots show the median, with the bounds of the box representing the 25th and 75th percentiles (interquartile range). Whiskers extend to the minimum and maximum values within 1.5 × the interquartile range, points beyond the whiskers represent outliers. **I** Autoregulatory model of alternative splicing-coupled NMD mediated by the essential exon of *fubl-1*: FUBL-1, an RNA-binding protein, binds to its own pre-mRNA and promotes exclusion of the essential exon #2. This exon loss causes a frameshift that introduces a premature termination codon, targeting the transcript for NMD. Through this mechanism, high FUBL-1 protein levels favor the unproductive splicing of its own gene thereby establishing a negative feedback loop that maintains homeostasis. Significance levels; *p  <  0.05, **p  <  0.01, ***p  <  0.001.

While the mechanisms of poison exon autoregulation have been well documented^58^, the in vivo organismal functional relevance is just beginning to emerge^59–61^, and whether essential exons have similar functional attributes remains unexplored. We therefore leveraged the powerful genetic tools available in *C. elegans* to explore the functional consequences of disrupting the *fubl-1* essential exon autoregulatory loop in vivo. We used CRISPR/Cas9 to generate precise genome edits forcing either inclusion (INC) or skipping (SKIP) of the non-triplet exon (Fig. 5A). qRT-PCR reveals that *fubl-1* expression increases in INC mutants, which force expression of the productive isoform, and decreases in SKIP mutants, which force expression of the unproductive isoform (Fig. 5B).

We performed phenotypic assays with these mutants to test for functional consequences of dysregulated *fubl-1* splicing. First, we performed competitive fitness assays, which report on general physiological defects such as growth, development, and reproduction^62,63^. We observe that, somewhat surprisingly, the INC mutant in which the productive *fubl-1* isoform is forced is the least fit (Fig. 5C). The loss-of-function and SKIP mutants (forcing the unproductive *fubl-1* isoform) resemble each other with mild fitness defects. These trends are also apparent in fertility assays, where INC mutants lay fewer eggs than the other genotypes (Fig. 5D). The most striking phenotype is in developmental timing, where INC mutants display a strong delay in developmental timing (Fig. 5E) while again the forced skip and null mutants exhibit similar, milder developmental delays. The pronounced phenotypic impact of increasing the functional *fubl-1* isoform, compared to the mild phenotypic impact of losing *fubl-1*, suggests that an excess of *fubl-1* is more detrimental to the organism than *fubl-1* deficiency. In such a circumstance, non-triplet autoregulation would be an ideal mechanism to prevent detrimental over-expression.

Since *fubl-1* and *fubl-3* are paralogous genes, we investigated whether *fubl-3* and *fubl-1* have partially redundant functions. To test this, we generated *fubl-1; fubl-3* double mutants and observed strong reductions in fitness (Fig. 5F) as well as in fertility (Fig. 5G) compared to wild-type or single mutants. On the other hand, the developmental timing of the double mutants does not differ significantly from that of the single mutants (Fig. 5H). Taken together, our data demonstrate that either too much *fubl-1* or too little *fubl-1* (especially when accompanied by too little *fubl-3*) results in negative consequences for worm health and development. This highlights the importance of stringent control of *fubl-1* gene expression mediated in part by non-triplet alternative splicing autoregulation (Fig. 5I).

### Non-triplet C-terminal isoforms with regulated cell-specific splicing

**Fig. 6:**
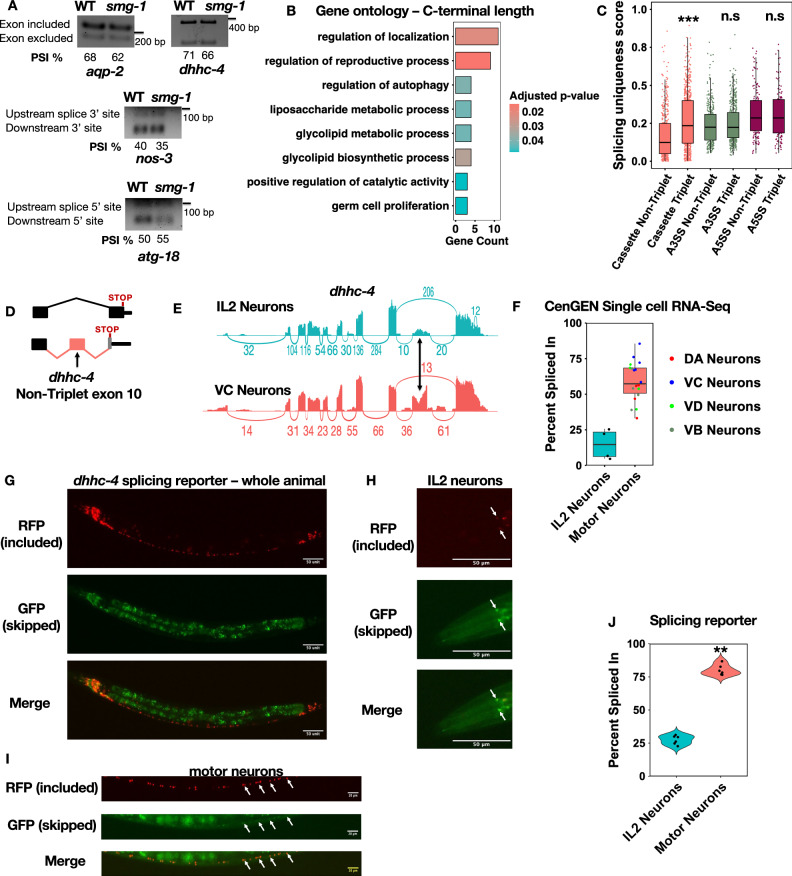
Alternative C-terminal isoforms resulting from non-triplet splicing are differentially expressed in the nervous system. **A** RT-PCR validation of NMD-resistant non-triplet splicing events including cassette (*aqp-2*, *dhhc-4*) and alternative 3′ splice site (*nos-3*, *atg-18*) events producing alternative C-terminal isoforms. **B** Gene ontology analysis for alternative C-terminal isoform genes, statistics were calculated using hypergeometric test (equivalent to a one-sided Fisher’s exact test), Benjamini Hochberg correction was applied to control the false discovery rate (FDR) for multiple comparisons, adjusted *p*-values < 0.05 were considered significant. **C** Uniqueness index for different splicing categories, each data point represents an individual splicing event, *n* = 717, statistical comparisons between triplet and non-triplets were performed using two sided Wilcoxon rank sum tests, cassette events (*p* < 2.2 × 10⁻¹⁶), A3SS (*p* = 0.191), and A5SS (*p* = 0.435), center line in the box plots show the median, with the bounds of the box representing the 25th and 75th percentiles (interquartile range). Whiskers extend to the minimum and maximum values within 1.5 × the interquartile range, points beyond the whiskers represent outliers. **D** Schematic showing stop-codon repositioning in *dhhc-4* by alternative splicing of non-triplet exon #10. **E** Sashimi plot displaying the usage of non-triplet exon 10 (indicated by a double arrow) in IL2 and VC neurons. **F** Percent spliced in values for *dhhc-4* exon 10 in the IL2 (*n* = 4) and in different classes of motor neurons including DA (*n* = 4), VC (*n* = 6), VD (*n* = 4), and VB (*n* = 4) based on single cell RNA-Seq data, center line in the box plots show the median, with the bounds of the box representing the 25th and 75th percentiles (interquartile range). Whiskers extend to the minimum and maximum values within 1.5 × the interquartile range, points beyond the whiskers represent outliers. (G-J) Splicing reporter images for the *dhhc-4* exon 10 expressed under the neuronal *rgef-1* promoter. **G** 10× (scale bar = 50 μm); **H** head region 20× zoomed with arrows indicating IL2 neurons (scale bar = 50 μm); **I** motor neurons zoomed in 20× (scale bar = 20 μm); (**J**) quantification of splicing reporter plasmid images, *n* = 6 worms, statistical tests performed using two-sided Wilcoxon rank-sum test (*p *= 0.00217). Significance levels: *p  <  0.05, **p  <  0.01, ***p  <  0.001.

We next focused on the second class of non-triplet alternative splicing outcomes, the generation of alternative C-termini lengths. In these cases, the reading-frame shift of the alternative isoform causes a change in the stop codon position (Fig. 2A), without resulting in NMD and without re-coding a substantial number of amino acids. Thus, between the two resulting alternatively spliced proteins, one encodes an extended C-terminus and a correspondingly shorter 3′ UTR, while the other encodes a truncated C-terminus and a longer 3′ UTR. We identified 136 such non-triplet alternative splicing events: 38 cassettes, 60 alternative 3’ splice sites, and 38 alternative 5’ splice sites (Fig. 2A, S2A). We further validated a subset of these alternative splicing events with RT-PCR, confirming substantial expression of both isoforms, and confirming their resistance to NMD (Fig. 6A). Gene ontology analysis reveals over-representation of terms related to protein lipidation and glycolipid biosynthesis (Fig. 6B, and S5B). This is in contrast with the NMD-sensitive class of non-triplets, which are enriched in mRNA splicing terms (Fig. 3J), indicating that these two classes of alternative splicing are substantially different in nature.

As with NMD-sensitive non-triplets, alternative C-terminal isoforms could arise either through splicing errors or through regulated alternative splicing. One piece of evidence often considered in favor of functional or regulated splicing is the existence of tissue-specific or cell-specific splicing patterns^64,65^. We therefore used deep neuron-specific transcriptomes generated by the CeNGEN consortium^66^ to compare cell-specific regulation of triplet and non-triplet alternative splicing. We previously developed an algorithm that determines the degree of cell-specific uniqueness exhibited by alternative splicing events^67^, with a score from 0 (no differential splicing across any cell types) to 1 (one cell type expresses 100% isoform A, while all other cell types express 100% isoform B). We find that non-triplet alternative splicing has similar uniqueness scores to triplet alternative splicing for 5’ and 3’ splice sites (Fig. 6C), while triplet cassettes have slightly higher average uniqueness values. Broadly speaking, therefore, non-triplet alternative splicing is subject to similar cell-specific regulation as triplet alternative splicing.

We focused on one such splicing event with an above-average uniqueness score (0.303) in the *dhhc-4* gene. *dhhc-4* encodes a palmitoyl transferase, a protein family responsible for attaching palmitate to cysteine residues on target proteins^68^. In *dhhc-4*, alternative splicing of the non-triplet exon generates transcripts with two different C-terminus lengths due to stop codon repositioning. Inclusion of the non-triplet exon results in a frameshift-induced stop codon that causes the C-terminus to lack the final 52 amino acids of the exon-skipped isoform (Fig. 6D). Both C-termini are predicted to be largely disordered (Fig. S5C), but with differences in predicted short linear motifs (Fig. S5D) including phosphorylation and ubiquitination sites^69–71^.

Single-neuron splicing data^67^ indicate that *dhhc-4* is alternatively spliced across different neuron types. For example, chemosensory IL2 neurons primarily skip the exon (15% included), while various motor neuron types primarily include the exon (*e.g*., VC motor neurons 70% included, Fig. 6E, F). To further verify this observation in vivo, we constructed a splicing reporter for the *dhhc-4* non-triplet exon and expressed it throughout the nervous system. We observed substantial diversity of splicing patterns, with some neurons expressing primarily RFP (included), others primarily GFP (skipped), and others both. Focusing on the IL2 neurons and motor neurons, we found primarily GFP expression in IL2 (Fig. 6H) and primarily RFP expression in motor neurons of the ventral nerve cord (Fig. 6I). Quantitative analysis of these reporters across multiple animals reveals splicing patterns similar to those reported by cell-specific RNA-Seq (Figs. 6J, 6F). This indicates that the *dhhc-4* non-triplet exon is subject to strict cell-specific splicing regulation and serves as an example of potentially hundreds of other non-triplet splicing events observed to undergo substantial cell-specific regulation (Fig. 6C).

### Regulation and function of non-triplet dual coding isoforms

**Fig. 7:**
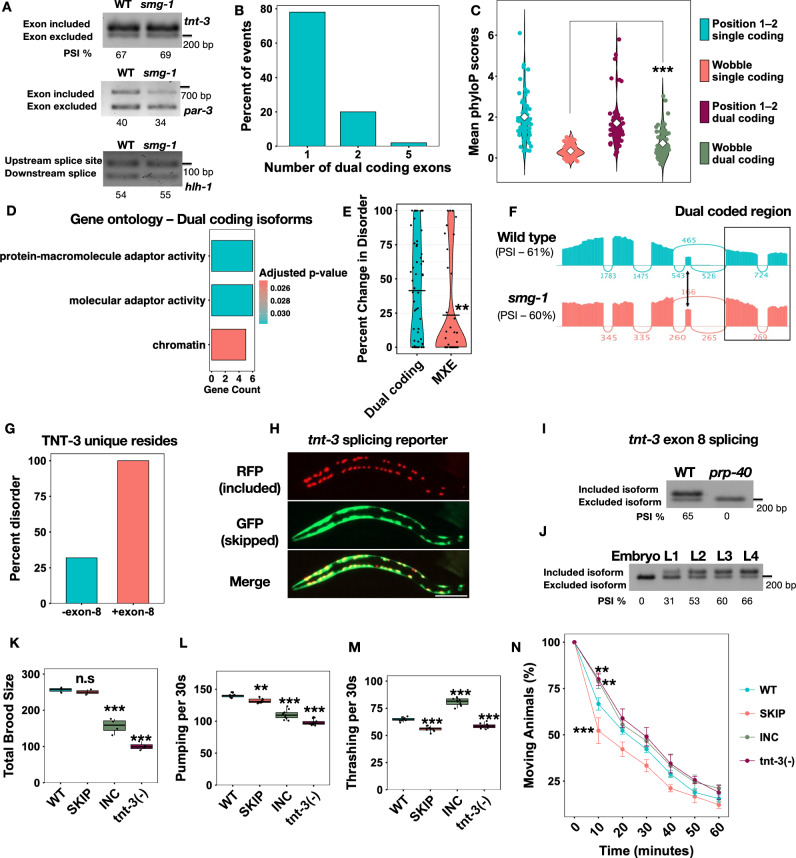
Non-triplet alternative splicing generates dual-coding isoforms with distinct reading frames and functional properties. **A** RT-PCR confirming the presence of the dual coded isoforms in wild-type and *smg-1* transcriptome. **B** Histogram of the number of dual coding exons that result across all dual-coding alternative splicing events (*smg-1* dataset). **C** Violin plot of mean phyloP scores comparing the wobble position in single and dual coded regions, each dot represents one event, white diamond = mean, statistics performed using one sided Wilcoxon signed-rank test, *p* = 1.10 × 10⁻⁴ (wobble single vs dual coding). **D** Gene ontology for genes encoding dual-coding isoforms, statistics were calculated using hypergeometric test (equivalent to a one-sided Fisher’s exact test), Benjamini Hochberg correction was applied to control the false discovery rate (FDR) for multiple comparisons, adjusted *p* values < 0.05 were considered significant. **E** Violin plot showing distribution of change in the percentage of intrinsic disorder-promoting residues between reference and alternate transcripts for frame-disrupting dual coding isoforms (*n* = 54) compared to frame-preserving mutually exclusive isoforms (*n* = 40), horizontal line = mean, statistical significance was assessed using a two sided Wilcoxon rank sum test (*p* = 0.00633). **F** Sashimi plot showing usage of the *tnt-3* non-triplet microexon in wild-type and *smg-1* worms, box indicates dual coded region. **G** AIUPred (per residue disorder propensity predicting tool) disorder scores for the unique amino acid residues in the microexon skipped and included isoforms of TNT-3 protein (refer methods). **H** Splicing reporter images for *tnt-3* microexon under the *myo-3* body wall muscle promoter (scale bar = 50 μm), representative image shown from four independent experiments with similar results. **I** RT-PCR of *tnt-3* microexon under wild-type and *prp-40* null background. **J** RT-PCR showing progressive increase in microexon usage during larval stages of development. **K** Brood size assay, *n* = 3 worms (WT vs INC (*p* = 9.76 × 10⁻⁶), WT vs SKIP (*p* = 0.952), WT vs tnt-3(-) (*p* = 1.56 × 10⁻⁷). **L** Pharyngeal pumping assay, *n* = 10 worms (WT vs INC (*p* < 2.2 × 10⁻¹⁶), WT vs SKIP (*p* = 0.00661), WT vs tnt-3(-) (*p* < 2.2 × 10⁻¹⁶). **M** Thrashing locomotory assay, *n* = 10 worms (WT vs INC (*p* < 2.2 × 10⁻¹⁶), WT vs SKIP (*p* = 8.44 × 10⁻⁹), WT vs tnt-3(-) (*p* = 1.09 × 10⁻⁵). For Figure K-M one way ANOVA was performed, the center line in the box plots show the median, with the bounds of the box representing the 25th and 75th percentiles (interquartile range). Whiskers extend to the minimum and maximum values within 1.5 × the interquartile range, points beyond the whiskers represent outliers. **N** Levamisole induced time dependent paralysis comparing wild-type and *tnt-3* mutants, Points represent the mean of three independent biological replicates (*n* = 30 worms per replicate), measure of center for the error bars is the mean; error bars represent standard deviation, statistical comparison at the 10 minute time point was performed using two way ANOVA (WT vs INC, *p* = 0.0091; WT vs SKIP, *p* = 5.99 × 10⁻⁴; WT vs tnt-3(-), *p* = 0.0024). Significance levels: *p  <  0.05, **p  <  0.01, ***p  <  0.001, n.s = not significant.

The final class of non-triplet alternative splicing is dual coding isoforms, in which a single nucleotide sequence encodes a stretch of amino acids in two different reading frames, with the reading frame being determined by the alternative splicing choice. We applied a minimum threshold of 60 dual-coding nucleotides, reasoning that this is sufficient to potentially encode a small protein domain^38,39^. We identified 54 such dual coding events (Fig. 2A, and S2A). We validated a subset of these events using RT-PCR, confirming abundant expression of both isoforms, as well as their resistance to NMD (Fig. 7A). Most dual-coding transcripts contain a single dual-coding exon, although some harbor up to five (Fig. 7B, and S6A).

Dual-coding nucleotide regions tend to contain fewer than 200 nucleotides but can extend to a maximum of 829 nucleotides (S6B). Dual-coding regions show significantly higher conservation of wobble-position nucleotides (*i.e*., the 3^rd^ nucleotide in each codon) compared to standard single-coding regions, consistent with the notion that both reading frames are under evolutionary selection (Fig. 7C). Gene ontology analysis reveals that dual-coding isoforms are enriched for DNA binding proteins (Fig. 7D), which is again distinct from the categories enriched for NMD-sensitive (Fig. 3J) and alternative C-terminal (Fig. 6B) classes.

Bioinformatic analysis of human transcriptomes predicts increased levels of structural disorder in dual coded regions for one of the alternative reading frames^20,72^. We likewise find that many dual coding regions resulting from non-triplet alternative splicing exhibit substantial increases in disorder propensity in one reading frame (Fig. 7E). This is higher than for other types of coding-sequence substituting alternative splicing such as (triplet) mutually exclusive exons (Fig. 7E).

To test for physiological functions of dual-coded isoforms in vivo we focused on *tnt-3*, which encodes one of four *C. elegans Troponin T* homologs. TNT-3 is expressed in muscle, where it interacts with tropomyosin, which in turn binds to actin. This prevents actin-myosin interaction in the absence of Ca2 + ^73^. Exon 8 of *tnt-3* is a highly-conserved (Fig. S2D) non-triplet “microexon” of only 28 nucleotides. If skipped, the canonical isoform is produced encoding 185 amino acids, including a conserved Troponin domain. If included, a reading frame shift occurs and a non-canonical isoform is encoded (Fig. 7F) of 83 alternative amino acids lacking the Troponin domain. Therefore, while the exon itself only encodes 9 amino acids, the resulting dual coding region encodes 83 alternatively translated amino acids. In line with the tendency of dual coding regions to be intrinsically disordered^20^, the 83 residues in the microexon-skipped isoform are predicted to be entirely disordered, in contrast with the ordered 185 residues in the microexon-included isoform (Fig. 7G). This disordered isoform is predicted to encode unique phosphorylation and protein-protein interaction motifs (Fig. S6C).

Both in vivo splicing reporters and RT-PCR confirm substantial splicing of both isoforms (Fig. 7A). Splicing reporters expressed in the muscles of both the body (Fig. 7H) and the pharynx (Fig. S6D) indicate substantial expression of both isoforms in both muscle types. The *tnt-3* microexon is unusual in that it is present in a muscle-specific gene, unlike the majority of microexons which are enriched in neuronal genes, where their regulation and function have been extensively studied^9^. We therefore tested whether, like neuronal microexons, splicing of the *tnt-3* microexon requires the spliceosomal component PRP-40^74,75^. Indeed, inclusion of this exon is completely lost in *prp-40* mutants (Fig. 7I, and S6E). Therefore, even though the *tnt-3* microexon is unusual in being both muscle-specific and non-triplet, it is nevertheless under strict regulation by the canonical microexon regulatory machinery.

RT-PCR analysis reveals a striking pattern of developmental regulation. Embryos express exclusively the canonical exon-skipped isoform, and progression through the developmental stages is marked by increasing inclusion of the microexon, reaching an inclusion level of 66% by the L4 stage (Fig. 7J). This dynamic expression is also observed in the in vivo transgenic splicing reporter (Fig. S6F). To determine whether this developmental shift is due to upregulation of the included isoform, downregulation of the skipped isoform, or both, we performed isoform-specific qRT-PCR. We find that the developmental pattern is driven primarily by the exon-included isoform, which progressively increases during development (Fig. S6G). Thus, embryos and younger worms predominantly express the canonical *tnt-3* isoform encoding the conserved Troponin domain, while adults express higher amounts of the non-canonical dual-coded isoform.

Given the strong regulation and striking developmental dynamics of the *tnt-3* non-triplet exon, we next asked whether the two dual-coded isoforms have distinct effects on worm development or muscle function. To do so, we made endogenous genome edits to either force exon inclusion (INC) or exon skipping (SKIP) (Fig. S6H). Loss-of-function *tnt-3* mutants and INC mutants exhibit fecundity defects, while SKIP mutants are indistinguishable from wild-type (Fig. 7K). Likewise, *tnt-3* loss-of-function mutants display defects in pharyngeal pumping that are again comparable with the INC mutants, while the SKIP mutants display only mild defects (Fig. 7L). In contrast, locomotory assays reveal INC mutants to be hyperactive, while SKIP and loss-of-function mutants are both modestly hypoactive (Fig. 7M). Finally, we performed pharmacological assays measuring paralysis kinetics following exposure to levamisole, an agonist of muscular acetylcholine receptors that causes excessive muscle depolarization and eventual paralysis^76^. We find that SKIP mutants are hypersensitive to levamisole, while the INC and loss-of-function mutants are resistant, indicating that the two *tnt-3* isoforms induce opposing dynamics of muscle activity (Fig. 7N). These results indicate that each of the dual-coding *tnt-3* isoforms possesses a unique function, and that both together are required for proper coordination of muscle contraction and excitability.

### Human transcriptomes harbor thousands of non-triplet alternative splicing events

**Fig. 8:**
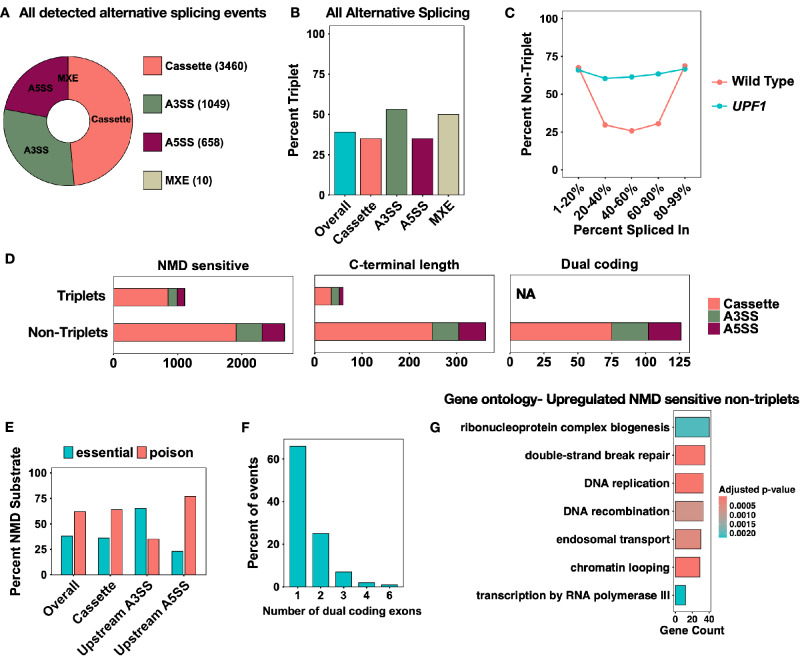
Human transcriptomes harbor thousands of non-triplet alternative splicing events. **A** Pie chart quantifying the distribution of total alternative splicing events (10–90% PSI in either wildtype or Δ|PSI | >10%) in *UPF1* knockdown HCT116 cells identified in four types: cassette, alternative 3’, alternative 5’ splice sites and mutually exclusive exons (MXE). **B** Histogram displaying the proportion of all alternative splicing events that are triplet in nature. **C** Fraction of non-triplet splicing events across PSI (percent spliced in) bins in wild-type vs *UPF1* knockdown cell line, percent non-triplet in 40-60% PSI range is 26% in WT cells compared to 61% in the NMD mutant cell line (**D**) Stacked histogram displaying the proportion of each outcome generated by triplet and non-triplet alternative splicing. **E** Histogram quantifying the contribution of essential and poison categories in cassette exons, upstream alternative 3’ and 5’ splice sites in generating NMD substrates (a subset of NMD sensitive cases whose alternative splicing changes the stop codon position identified from our computational pipeline). **F** Histogram indicating the proportion of dual coding exons. **G** Gene enrichment analysis of upregulated (log_2_FoldChange > 0.50, p-adjusted <0.05) NMD sensitive non-triplet category, statistics were calculated using hypergeometric test (equivalent to a one-sided Fisher’s exact test), Benjamini Hochberg correction was applied to control the false discovery rate (FDR) for multiple comparisons, adjusted *p* values < 0.05 were considered significant.

To test whether our observations on *C. elegans* non-triplets apply to human transcriptomes as well, we analyzed RNA-Seq from a recent high-efficiency auxin-induced degron knockdown of UPF1 in HCT116 colorectal cancer cells^77^. Using our same bioinformatic pipeline (see Fig. 1), we found broadly similar patterns of non-triplet alternative splicing, with a few modifications (Supplementary Data 5). First, the total number of alternative splicing events is much higher in humans (Figs. 8A, 1B, C), in agreement with previous observations^78^. Second, the fraction of splicing events that are non-triplet is much higher in humans (61%), closely matching a previous report in lymphoblastoid cell line^15^ (Figs. 8B, 1D, F). Third, we identified a number of NMD sensitive non-triplet mutually exclusive splicing events in human (Fig S7A) but not worm (Fig. 1E, G) transcriptomes.

Similarities between worm and human transcriptomes include the observation that de novo levels (without NMD) of non-triplet alternative splicing are substantially higher than the steady-state levels of alternative splicing (Figs. 8C, 2B, and S2E), and this is even more pronounced in the human data (Fig. 8C). Likewise, the relative abundance of the different functional outcomes of non-triplet splicing is similar between the worm and human data (Figs. 8D, S7B, 2A, and S2A). In humans, this amounts to thousands of NMD sensitive non-triplet splicing events, and hundreds of alternative C-terminal and dual-coding isoforms.

Additional similarities include the large fraction of essential exons, in addition to their better-known counterpart poison exons (Fig. 8E, and S7C), as well as similarities in the metrics of dual coding exons (Figs. 8F, 7B). As in worms, gene ontology terms for NMD-sensitive non-triplet splicing are enriched in RNA-related processes, while the other categories of splicing yield distinct and non-overlapping ontology terms (Fig. 8G, S7D–E). Broadly speaking, therefore, the molecular outcomes for alternative non-triplets are similar between worms and transcriptomes, but even more abundant in human transcriptomes, both in terms of total quantity and in terms of proportion of all alternative splicing events. Taken together, these results indicate that non-triplet alternative splicing is broadly used to regulate gene expression and generate protein-coding diversity across metazoan transcriptomes.

## DISCUSSION

Here we demonstrate that non-triplet alternative splicing is abundant, strictly regulated, and functionally important for *C. elegans* development and health. This is in contrast with earlier studies, which often consider such splicing to be evidence of noise, errors^15^, or non-functional splicing^79,80^. We categorize non-triplet splicing into three groups based on their molecular effects: NMD-sensitive splicing, alternative C-terminal lengths, and dual coding isoforms – and identify dozens to hundreds of such splicing events in each category. These numbers are surely underestimates: first, because of the conservative thresholds used to classify alternative splicing (requiring 10-90% inclusion); second, because our whole-animal transcriptomes likely miss events with highly tissue-specific or cell-specific splicing patterns. This notion is supported by comparing whole-animal data (684 non-triplets detected) with neuron-specific data (Fig. 5C) where 888 non-triplets are detected.

Analysis of human transcriptomes reveals similar patterns of non-triplet splicing, but to an even greater degree, with thousands of NMD-sensitive isoforms and hundreds of alternative C-terminal and dual-coding isoforms. Relative abundances of molecular categories are similar between worm and human, but the overall number is much higher (6-fold) in the human transcriptome. As with the worm data, these are likely underestimates given that the data come from a single cancer cell line.

Substantial work has been performed on frame preserving stop codon-containing poison exons that trigger NMD^25^. We find that many more NMD substrates are collectively generated by non-triplet essential exons, alternative 3’ splice sites, and alternative 5’ splice sites. This substantially increases the scope of the poison/essential splicing phenomenon and highlights additional alternative splicing mechanisms that can be leveraged for splicing/NMD control of gene expression. We demonstrate that essential non-triplet exons, much like canonical poison exons, confer autoregulatory feedback to control splicing factor levels, and that this autoregulation is essential for organismal fitness and development. Together these findings expand the scope and impact of the exciting field of splicing factor autoregulation^59–61,81^.

We also identify many non-triplet alternative splicing events that do not cause NMD but rather generate C-terminal protein coding differences. This occurs by the alternative reading frame producing a shorter protein coding region (alternative C-terminal length) or a substantial stretch of re-coded nucleotides (dual coding isoforms). Our systematic sequencing and bioinformatic approaches reveal that these splicing categories are abundant and are regulated in much the same way as their better-studied triplet counterparts. A striking example is found in the gene *tnt-3*, which harbors an alternative non-triplet microexon. While the exon itself encodes only 9 amino acids, its splicing dictates the selection of 83 amino acids in the downstream dual-coding region. Both isoforms are functionally important, as forcing exon inclusion causes hyperactive muscle activity, while exon skipping causes hypoactive muscle activity. This functional example aligns with existing examples of isoform-specific functions for C-terminal length^17,82^ and dual-coding isoforms^21,27,28,83^ generated by non-triplet alternative splicing. Together with our global analysis of NMD-resistant non-triplets, these examples suggest an even broader role for alternative splicing in shaping the protein-coding transcriptome.

The extent to which alternative splicing is functionally important to an organism versus a result of noise or splicing errors is an important question and matter of ongoing debate^79,84–86^. One point often considered in favor of the noise/error hypothesis is non-triplet splicing that leads to reading-frame shifts and often causes premature stop codons and NMD^12^. We provide evidence that this is an oversimplification: non-triplet alternative splicing often yields stable isoforms that are strictly regulated and functionally important to the organism. Even those that are unstable and NMD-sensitive can provide important functional regulation of gene expression^85^. In the case of *fubl-1*, ~80% of the de novo spliced transcripts are of the NMD-sensitive unproductive isoform and forcing only the productive isoform to be produced causes defects in organismal fitness, development, and fertility. Together these results reveal an even more diverse and heterogenous suite of roles for alternative splicing, including the generation of poison/essential exons and splice sites, proteins with variable C-terminal lengths, and proteins with re-coded amino acid sequences.

## METHODS

### *C. elegans* strain maintenance

*C. elegans* were maintained by standard techniques^87^ on NGM agar plates seeded with OP50 *E.coli*.

### Strains used

ADN1542 (PHX7865 *fubl-1a(syb7865)* null mutation – small 139 bp deletion, chrV:10,237,409-10,237,547), ADN1249 (PHX5655 *fubl-1(syb5655)* forced inclusion → in-frame), ADN1250 (PHX5667, *fubl-1(syb5667)*, forced skipping → out-of-frame), and ADN00001753 (PHX7883 *fubl-3b(syb7883)* null mutation - 149 bp deletion, chrX:17,435,745-17,435,893). *smg-1* (TR1331), *smg-2* (CB4043), ADN1743 (Ex[*rgef-1::dhhc-4* splicing reporter]), ADN1742 (Ex[*myo-2::tnt-3* splicing reporter]), Ex[*tnt-3::myo-3* splicing reporter], ADN1717 (AN130, PHX9050 *tnt-3(syb9050)*, forced microexon skipping), ADN1711 (AN129, *tnt-3(syb8820)*, forced non-triplet microexon inclusion), ADN1691 (*tnt-3* transgene balanced by *prp-40/elt-1*), *tnt-3(ok1011)* loss-of-function mutant, ADN1692 (*fubl-1; fubl-3* double mutant), ADN00001687 (Ex[*rgef-1::fubl-1* splicing reporter]), ADN1689 (*rgef-1::fubl-3* splicing reporter]).

### RNA-Seq and raw data processing

Total RNA was extracted from synchronized L4-stage larvae using Trizol. Three biological replicates were extracted per genotype, and mRNA was purified from each using NEBNext® Poly(A) mRNA Magnetic Isolation Module. cDNA libraries were prepared using NEBNext® Ultra™ II RNA Library Prep Kit for Illumina. Paired end 150 bp reads were generated on an Illumina HiSeq 2000. Reads were then mapped to the worm genome using STAR version 2.5.3a^88^. Differential gene expression was determined by DESeq2^36^ (1.36.0) and alternative splicing was analyzed by JUM (2.0.2^35^),requiring a minimum of five junction-spanning reads per event. Splicing events with BH adjusted P values < 0.05 were considered statistically significant. Events were classified as alternative if either the wild-type PSI ranged between 10–90% or if a significant splicing change was observed in NMD mutant samples (ΔPSI | > 10%). Events were further categorized as NMD-sensitive or NMD-resistant based on an absolute ΔPSI threshold of 10%. For alternative 3′ splice site (A3SS) and alternative 5′ splice site (A5SS) events, PSI values were assigned to the splice site that is upstream in genomic coordinates irrespective of the strand orientation. For all event types, the genomic coordinates of the alternatively spliced region were identified, and events were classified as triplet or non-triplet depending on whether the length of the alternative region was divisible by three. In addition to JUM, aligned BAM files were analyzed using MAJIQ^89^ and VOILA v2.5.7. For cassette exon events, only non-complex local splicing variation (LSV) events were retained for downstream analysis. Events with a posterior probability of splicing change > 0.95, as reported by MAJIQ, were considered high-confidence differentially spliced events. Categorization into NMD-sensitive isoforms, alternative C-terminal isoforms, and dual-coding isoforms was done using the computational pipeline described below.

### Bulk RNA-Seq analysis of UPF1 knockdown HCT116 cells

Raw FASTQ files corresponding to UPF1 auxin-inducible degron (AID) cells were downloaded from the European Nucleotide Archive (ENA) under accession Array Express: E-MTAB-13787^77^. 48-hour indole-3-acetic acid (IAA) treatment condition to degrade UPF1 was selected for the analysis. Reads were aligned to the human reference genome (GRCh38, GENCODE release 42 transcript annotations) using STAR^88^ (v2.7.10b) with default parameters, and splice junctions were quantified as described for the *C. elegans* analysis using JUM^35^.

### Gene Ontology

Gene Ontology enrichment analysis was performed using the *clusterProfiler* R package (version 4.16.0)^90^ applying a *p* value cutoff of 0.05 and *q* value cutoff of 0.05 for multiple-testing correction (Benjamini–Hochberg method). To avoid redundancy, GO terms which were subsets of another larger category were removed and the remaining unique terms were used for visualization and reporting.

### Fluorescence microscopy

Zeiss epifluorescence microscope equipped with a CCD camera was used to take images with a 10X and 20X objective. Both wild-type and null mutants were exposed for the same duration to ensure consistency. If a specific region was selected in GFP, the corresponding region in RFP was analyzed to measure their respective intensities. PSI was then calculated using a formula where RFP represents inclusion and GFP represents skipping. Images were processed and analyzed in ImageJ.

### Levamisole assay

30 L-4 animals were transferred to 12 mm unseeded agar plate and 1 ml of 0.4 mM levamisole solution prepared from 100 mM stock solution in 1x M9 was added to the plate so that all animals float freely in the solution. This point was noted as time=0, for every 10 minutes until 60 minutes the number of moving animals were recorded.

### Brood size assay

A single L-4 stage animal was seeded on a NGM plate and incubated at 20 °C. Animals were transferred to a new plate every 24 hour until egg production stopped. The number of eggs in each plate was counted to calculate eggs produced each day. Total Brood size was calculated as the sum of eggs produced each day. Experiment was performed in triplicates.

### Developmental timing assay

For the developmental timing assay, synchronized L1-stage worms of each genotype were plated on NGM plates at 20 °C and cultured for 36 hours. The proportion of worms that reached the L4 stage was then determined by scoring vulval morphology across the plated population, and the results were expressed as the percentage of L4 worms.

### Competition assay

3 L-4 stage animals of each genotype were placed together with wild type on a seeded NGM plate. Worms were allowed to grow, reproduce, and compete for 5 days at 20 °C. For each replicate, 100 worms were randomly scored from a defined plate region and relative fitness value for each genotype was calculated using the formula *F* = (# mutant/# total)/50%. This normalization assumes that wild-type fitness = 1.0 (i.e., 50% of the population in a neutral competition). If a mutant produces fewer offspring (i.e., <50% of total), F < 1.0, indicating reduced fitness. If F > 1.0, the mutant outcompetes the wild type.

### Splicing Reporter

Two color splicing reporters were generated for non-triplet exons in *fubl-1, fubl-3, dhhc-4* and *tnt-3* by synthesizing the flanking exons and introns (Invitrogen) and inserting this region into a dual GFP-RFP cassettef^91,92^ followed by recombination into appropriate promoter constructs.

### Quantitative- PCR

Total RNA was extracted from L4-staged animals using Tri-Reagent followed by purification with the Direct-zol RNA MiniPrep Kit (Zymo Research, Genesee Scientific) according to the manufacturer’s instructions. Complementary DNA (cDNA) was synthesized from 300 ng of total RNA using the Verso cDNA Synthesis Kit (Thermo Fisher Scientific) in a 20 μL reaction volume, following the manufacturer’s protocol. Quantitative PCR was performed using 1 μL of cDNA per reaction with PowerUp SYBR Green Master Mix (Thermo Fisher Scientific). The *act-1* gene was used as a housekeeping control for normalization. Data analysis was done using the ΔCt method (Ct_target − Ct_actin). Statistical significance was assessed using ΔCt values across the three biological replicates.

### RT-PCR

RNA was extracted as from L-4 animals as described above. RT-PCR was performed using the Luna script multiplex one step kit (NEB) according to the manufacture’s protocol.

### Statistics & reproducibility

All statistical tests were performed in R version 4.3.3. Information about the type of statistical test used is mentioned in the figure legends wherever appropriate. Statistical significance was defined as *p  <  0.05, **p  <  0.01, ***p  <  0.001, n.s (not significant). All microscopy experiments were repeated independently at least four times with similar results. The exact number of biological replicates used for quantification is provided in the corresponding figure legends of the splicing reporter quantification plots (Figs. 4K, 4L and 6J). For RT–PCR agarose gel experiments (Figs. 3E, 4F, 4G, 4M, 4N, 6A, 7A, 7I and 7J), representative images are shown. The experiments were repeated independently at least two times with similar results. No statistical method was used to predetermine sample size. No data were excluded from the analyses. The experiments were not randomized. The investigators were not blinded to allocation during experiments and outcome assessment.

### RT-PCR Primers

Oligonucleotides were synthesized by Invitrogen.

**Table:** 

**Gene**	**Sequence 5’- 3’**
*aqp-2 fw*	CGGAGCTGTCGTCGGAATC
*aqp-2 rv*	GGAGGCAGGATGGTAGCATTG
*atg-18 fw*	AGTCAGACACTAGTCGAGCA
*atg-18 rv*	CAGGATCGTCCATATTCGGAAC
*rnf-113 fw*	GTGATTCAAAAGCGACGTCG
*rnf-113 rv*	CGTCAGAGTCGTCAGAATCA
*rsr-1 fw*	ATGAAGTTTGAGCCGCAGT
*rsr-1 rv*	GTATATCGTTGACACGGGCC
*stip-1 fw*	ATACGCAATGAATCCAGGCG
*stip-1 rv*	CATCGTCATCATCGTCGCT
*act-1 fw*	ACGACGAGTCCGGCCCATCC
*act-1 rv*	GAAAGCTGGTGGTGACGATGGTT
*bir-2 fw*	AACATCGCCTGTACTTCCG
*bir-2 rev*	CAGCATACAAAACGGGCAT
*nos-3 fw*	GAGGATCAGGAGGAGGACAACG
*nos-3 rv*	TGTCGTTGGACATCGGTGC
*dhhc-4 fw*	CAACCACGGGATTCGAGC
*dhhc-4 rv*	TACGCCGTCACCGAGT
*tnt-3 fw*	AAGGCTGCTCAGAACGACAAGTTT
*tnt-3 rv*	TCGGTCTCGAGTTTGCATATTCTC
*fubl-3 qpcr_fw*	AACCACACTTCGACGCAG
*fubl-3 qpcr_rv*	AAGGTCTACGTGTGTGCTC
*fubl-1 qpcr_fw*	ACTGCTTCAGCTTTGTCGTG
*fubl-1 q_pcrrv*	ACGACGACACCTACCGTT
*fubl-1 fw*	GGAAGGATTTAACCCGCAGGTAATC
*fubl-1 rv*	CTTGGCGGGAAGCGAATCTGAA
*fubl-3 fw*	ACTCCCTCCTCATCTAACACAC
*fubl-3 rv*	GGACGGCGACTTTGACGATG
*rsp-7 fw*	AACGGCTGGTCAGCG
*rsp-7 rv*	TAAGCCGGTAGTCCGAGC
sfa-1 fw	CCGGGAATGCCAACAATTCTA
sfa-1 rv	CTGTTCCAATTCCTGCCGTTTC
fubl-1 (exon+2) qpcr_fw	AGGATGAGGAATATTCGCTTCCC
fubl-1 (exon+2) qpcr_ rv	TCGGATACTTCTCGTTAACGGC
fubl-1 (exon-2) qpcr_fw	TAACCCGCAGATTCGCTTCCC
fubl-1 (exon-2) qpcr_ rv	TCGGGAATCGGATACTTCTCGTT
tnt-3 inc qpcr_fw	AGGCTGCTCAGAACGACAAG
tnt-3 inc qpcr_rv	GATAATCGTGTACGCTTCGCGTC
tnt-3 skip qpcr_fw	AGAAGGCTGCTCAGAACGACA
tnt-3 skip qpcr_rv	GGAATGCACGCTTCGCGT
par-3 fw	TCATCTGAACGTGCTCCACC
par-3 rv	GGAATTTGTTGATGGTAGGCGAC
hlh-1 fw	TGACGGATTCGGACGAC
hlh-1 rv	AATCTGTCCAAAGAGTTTCGC

### Categorization of alternative splicing molecular effects

*Simulation Code* - The workflow integrates genome annotations, FASTA sequences, and custom scripts to simulate alternatively spliced isoforms^93^.

### Data sources

*Genome Annotation*: WS290 GTF file for *C. elegans*, downloaded from Worm base.

*Genome Sequence*: Raw FASTA file for the *C. elegans* genome from Worm base.

*JUM output*: Processed files from RNA-Seq alternative splicing analysis.

*Coordinate Conversion*: To simulate inclusion or exclusion of alternative cassette exons, the 3’ and 5’ splice site coordinates from JUM (0-based) were converted to 1-based GTF coordinates for compatibility with the annotation file.

*Reference Transcript Selection*: For each gene, the script rt.py identifies the isoform with the widest CDS as the reference transcript. The script status.py determines the status of the alternatively spliced region in the reference transcript: Included: If the splice region overlaps with the CDS of the reference transcript. Skipped: If the splice region does not overlap with the CDS.

*Splicing Simulation and Sequence Extraction*: Using the script simulation.py, the following steps are performed for each reference transcript:

*Exon and CDS Extraction*: Exon and CDS annotations are retrieved from the GTF file. Each exon’s DNA sequence is extracted from the FASTA file.

*Spliced Transcript Construction*: CDS exons are sorted in the order of transcription (5’ to 3’). The spliced transcript is generated by concatenating the CDS exon sequences. If the transcript’s end extends beyond the CDS end (i.e., includes a 3’ UTR), the 3’ UTR sequence is extracted from the genome and appended to the spliced transcript.

*Reference Stop Codon:* Starting from the first ATG (CDS start), the genomic coordinate of the first in-frame stop codon is identified and reported in the sc_ref column.

*Alternative Transcript Construction*: An alternative transcript is generated based on the status of the splice region. If the reference transcript includes the splice region, the alternative transcript excludes it. If the reference transcript skips the splice region, the alternative transcript includes it. The first in-frame stop codon in the alternative transcript is reported in the sc_alt column.

### Calculated metrics

In_frame_sc: Indicates whether the alternative splice region contains an in-frame stop codon (YES or NO).

Exon Junction: Defined as the start coordinate of the last annotated exon (cassette and 5’ splice site) and as end coordinate of the penultimate exon (alternative 3’ splice site). 25 Nucleotides upstream of this coordinate location was considered as the site for Exon Junction Complex deposition^85^.

Distance_sc_ref: The nucleotide distance in the spliced reference transcript between the exon junction and the sc_ref stop codon.

Distance_sc_alt: The nucleotide distance between the exon junction and the sc_alt stop codon. Positive values indicate the stop codon is upstream of the junction; negative values indicate it is downstream.

Exon_after_sc_alt: Number of exons downstream of the sc_alt stop codon in the alternative transcript, excluding the exon containing the stop codon.

Exon_after_end: Number of exons downstream of the splice region’s end coordinate in the reference transcript.

Recoded_exons: Number of coding exons between the splice region’s end coordinate and the sc_alt stop codon, including the exon containing the stop codon.

Ntd_recoded_ref: Number of nucleotides between the splice region’s start coordinate and the sc_ref stop codon.

Ntds_altv_rec: Number of nucleotides between the splice region’s start coordinate and the sc_alt stop codon.

Dual_coding: Number of nucleotides common to both reference and alternative transcripts (i.e., between the splice region’s end coordinate and the sc_alt stop codon).

End_UTR: Genomic coordinate of the last nucleotide of the last annotated exon.

Dist_ref_UTR: Length of the 3’ UTR in the reference transcript (distance from sc_ref to end_UTR).

Dist_alt_UTR: Length of the 3’ UTR in the alternative transcript (distance from sc_alt to end_UTR).

#### Filtering invalid cases

To ensure that only cleanly mappable and biologically interpretable alternative splicing events were analyzed, stringent quality filters were applied. Events exhibiting > 10% ΔPSI but were statistically insignificant were excluded from further analysis. Splice regions were excluded from downstream analyses if they fell outside the annotated coding sequence (CDS) of the reference transcript, overlapped multiple CDS exons, or belonged to genes lacking a defined reference isoform. For cassette-type events, alternate exons containing an in-frame ATG corresponding to the first exon of an alternate transcript were also excluded. First exons were identified using a custom Python script (*first_exons.py*) and further assessed for upstream and downstream RNA-seq read support. Alternative 3′ and 5′ splice-site (A3SS and A5SS) events with length exceeding 120 nucleotides were removed to avoid mis-categorization of first/last exons as alternative 5’/3’ splice sites. Finally, technically invalid or unresolvable cases, including transcripts lacking a stop codon, were excluded and logged in *error.tsv*.

#### Output

The final output is a TSV file containing the processed splicing events with all calculated metrics, alongside separate TSV files for excluded cases. In addition, a separate directory containing the alternative sequence and the simulated region is generated.

### Disorder scores

Dual coding isoforms and *dhhc-4* **-**Both the reference and alternative sequences obtained from our simulation pipeline were translated into proteins. To identify unique residues resulting from the frameshift, the sequences were compared and the divergent regions specific to each isoform were extracted starting from the first differing amino acid. AIUPred^94^ (version 2.x) was run with GPU acceleration and default smoothing parameters to calculate per-residue disorder scores for the sequences in both the reference and alternate isoforms. Percent disorder was defined as the proportion of residues with a score greater than 0.5.

### Mutually exclusive exons

Dataset from Wolfe et al.^67^. was combined with our RNA-Seq data to obtain a comprehensive list of frame preserving mutually exclusive exons with their coordinates. With modification to our previous script, the script mxe.py determines status of the mutually exclusive exons in the reference transcript as either included or skipped. An alternate transcript sequence was then constructed, reversing the status of the reference transcript. Both the transcripts were translated into a protein sequence with codons mapped to their corresponding exons to identify the protein residues encoded by the mutually exclusive exon. AIUPred (version 2.x) was run with GPU acceleration and default smoothing parameters to calculate the per residue scores for the mutually exclusive amino acid sequence for both reference and alternate sequence. Percent disorder denotes the percentage of amino acid residues with a score above 0.5.

### Uniqueness score

Values were obtained from the Wolfe et al.^67^ uniqueness index with one modification: scores were normalized to the total number of cells in which the splicing event is detectable, such that the uniqueness value reported here is a measure of the degree of uniqueness per represented cell.

### ELM analysis

Short Linear Motifs (SLiMs) were identified using the ELM database^95^ via the command-line tool gget. Isoform FASTA sequences were queried with *gget elm*. Motif annotations retrieved in CSV format were analyzed to get enrichment terms.

### Phylogenetic conservation analysis

PhyloP conservation scores are computed for ce11 phyloP 26-way multiple species alignment (*ce11.phyloP26way*), which measures nucleotide-level evolutionary conservation across 26 nematode species. Scores were extracted using bigWigSummary, sampling one value per base across the alternative region. Positions lacking phyloP coverage were excluded, and the mean phyloP score across all valid nucleotides within the region was calculated to obtain an event-level conservation score.

For Fig. 7C, dual-coding regions were identified by comparing coding sequence (CDS) between reference and alternatively spliced transcript isoforms. For each nucleotide position within the alternative region, codon positions were computed for both isoforms. Shared dual-frame positions were defined as nucleotides that remained within the CDS in both isoforms but occupied different codon positions across reading frames. Wobble positions were defined as third codon positions in the reference frame. For each event, a length-matched single-coding control region was selected from the same CDS, upstream or downstream of the alternative region in a strand-aware manner, excluding overlap with dual-coding positions. Phylogenetic conservation of dual-coding and control single coding regions was assessed using nucleotide-level phyloP scores, with both per-base and region-level summaries used for downstream analyses.

### Reporting summary

Further information on research design is available in the Nature Portfolio Reporting Summary linked to this article.

## Supplementary information

Supplementary Information

Description of Additional Supplementary Files

Supplementary Data 1

Supplementary Data 2

Supplementary Data 3

Supplementary Data 4

Supplementary Data 5

Reporting Summary

Transparent Peer Review file

## Source data

Source Data

## Data Availability

The RNA-Seq data generated in this study have been deposited in the NCBI GEO database under accession code GSE313026 (https://www.ncbi.nlm.nih.gov/geo/query/acc.cgi?acc=GSE101099). The human RNA-Seq data re-analyzed for this study have been deposited in the European Nucleotide Archive under the accession code E-MTAB-13787. Source data are provided with this paper.

## Code availability

All the python and R scripts used for simulating alternative transcripts, processing JUM can be accessed publicly from the github repository (https://github.com/shameerudeen/Non-Triplet-splicing) and archived on Zenodo^93^ (10.5281/zenodo.19042709).
