# Supplementary material for: Pervasive non-triplet alternative splicing drives functional isoform diversity
Source: Nat Commun. 2026 Apr 10;17:5112. doi: 10.1038/s41467-026-71615-5 (PMC13247117; doi:10.1038/s41467-026-71615-5)
Supplement: Supplementary file 1 — Supplementary Information [file 41467_2026_71615_MOESM1_ESM.pdf]

Supplementary information:

Pervasive non-triplet alternative splicing drives functional isoform diversity

Shameerudeen Athavudeen[1,2], Neethu Issac[3,4], Adam Norris[1,2,5*]

1. Department of Biochemistry, University of California, Riverside, CA 92521, United States.

2. Center for RNA Biology and Medicine, University of California, Riverside, CA 92521, United States.

3. Department of Immunology and Theranostics, Arthur Riggs Diabetes and Metabolism Research Institute, Beckman Research Institute of City of Hope, Duarte, CA, United States

4. Irell & Manella Graduate School of Biological Sciences, City of Hope, National Medical Center, Duarte, CA, United States.

* Correspondence: adam.norris@ucr.edu

Containing:

Figures S1-7

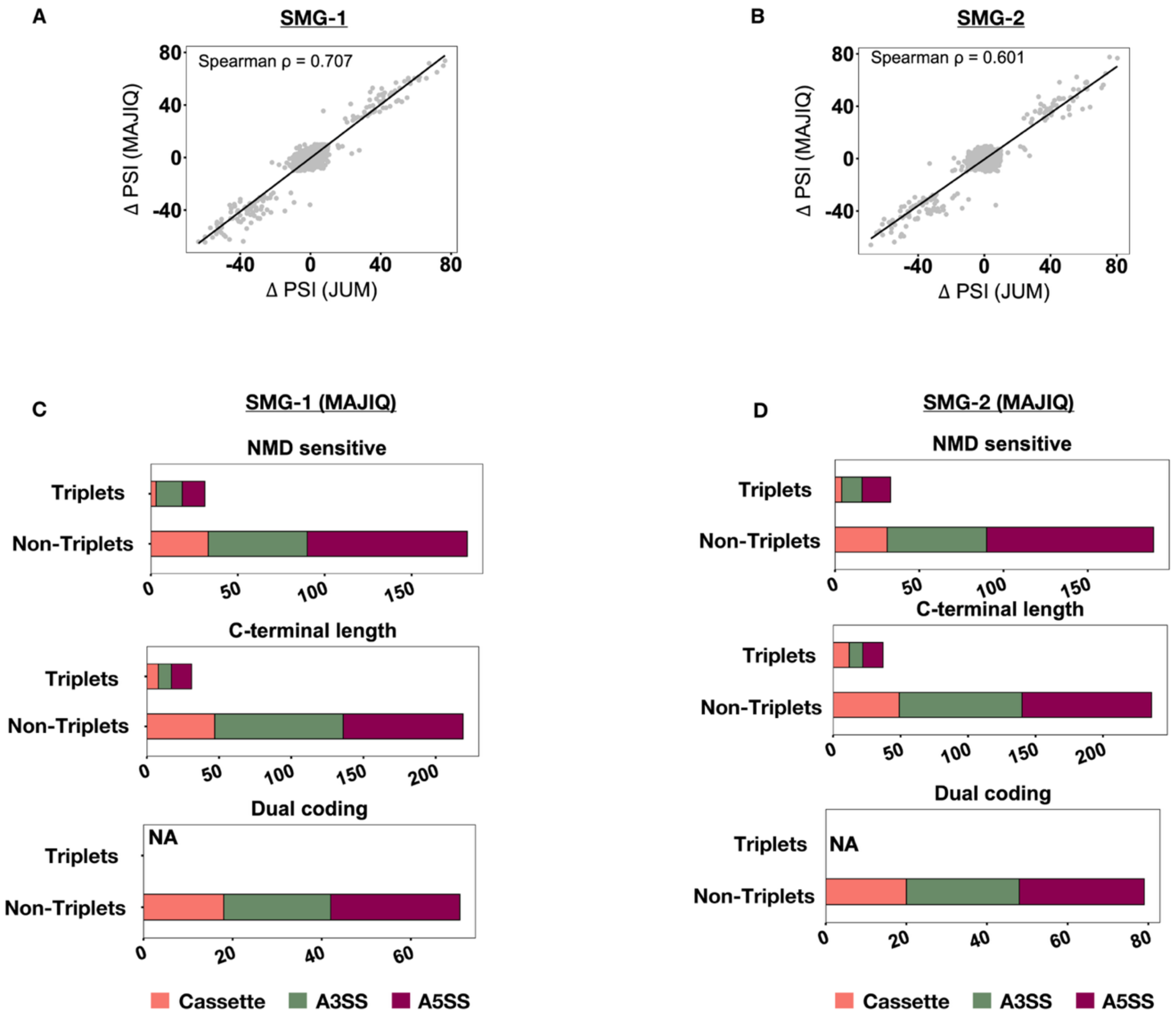

Figure S1: Concordance of splicing changes detected by JUM and MAJIQ. (A-B) ΔPSI correlation plot for the common alternative splicing events identified using JUM and MAJIQ for smg-1 and smg-2 respectively. (C) Molecular categorization of non-triplet splicing outcomes into NMD sensitive, alternative C- terminal length and dual coding isoforms for the events detected by MAJIQ in smg-1 for comparison with JUM refer Fig.2A. (D) Categorization for smg-2, for comparison with JUM refer Fig.S2A

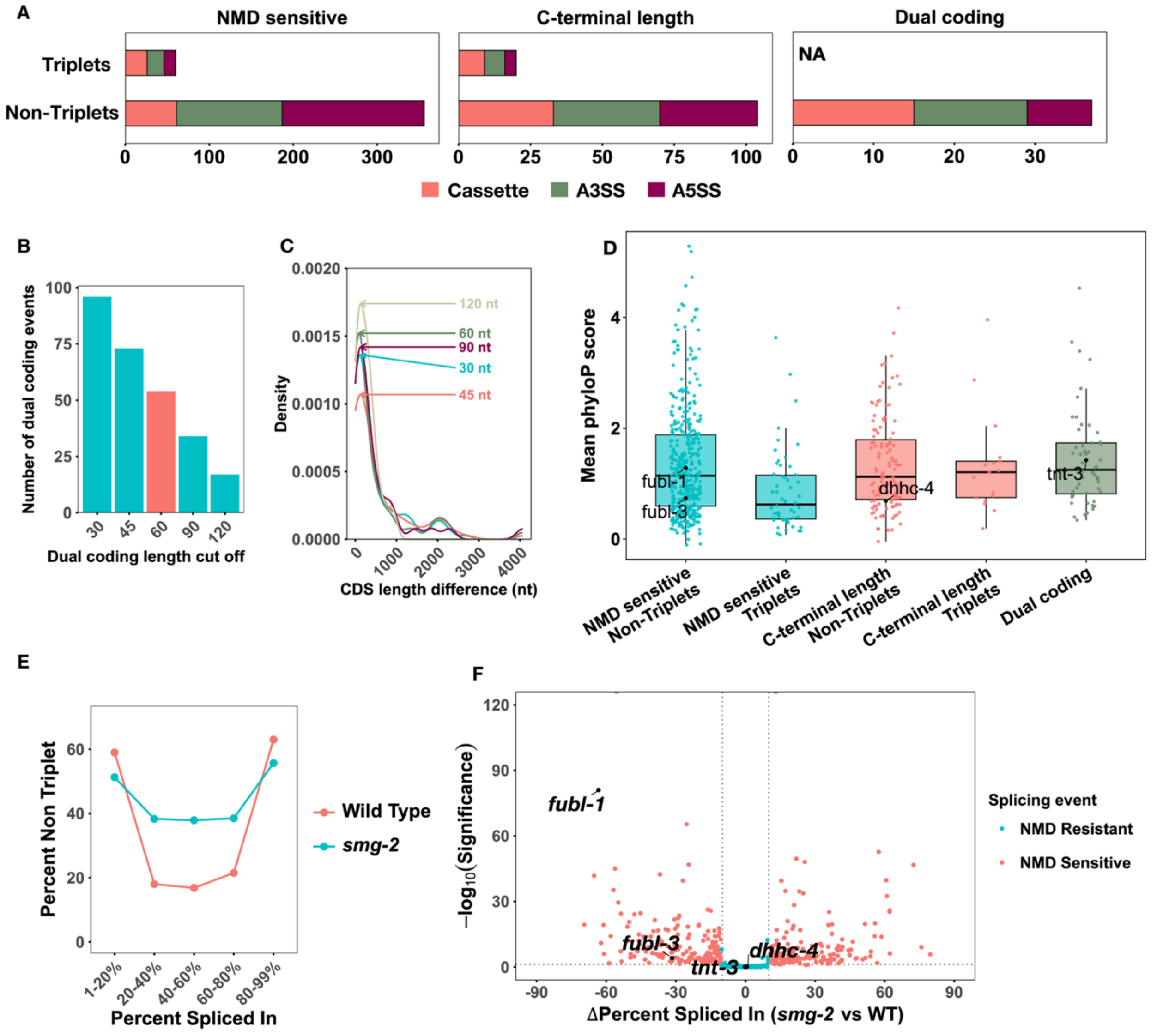

Figure S2: Molecular outcomes of non-triplet splicing. (A) Molecular categorization of non-triplet alternative splicing events identified in smg-2 mutants into NMD-sensitive, alternative C-terminal length, and dual-coding isoforms, analogous to the classification shown for smg-1 in Fig. 2A. **(B)** Number of dual coding events across different dual coding cutoff lengths for smg-1. **(C)** Distribution of CDS length difference between the two dual coding isoforms based on the cutoffs in B. **(D)** Distribution of mean phyloP conservation scores for the three outcomes defined in Fig. 2A. The center line in the box plots show the median, with the bounds of the box representing the 25th and 75th percentiles (interquartile range). Whiskers extend to the minimum and maximum values within 1.5 × the interquartile range, points beyond the whiskers represent outliers. **(E)** Distribution of non-triplet alternative splicing events across PSI bins in wild-type and smg-2, demonstrating increased representation of non-triplet events at intermediate PSI values. **(F)** Examples of non-triplet alternative splicing events exhibiting large ΔPSI values between wild-type and smg-2, consistent with efficient degradation of one isoform by nonsense-mediated decay.

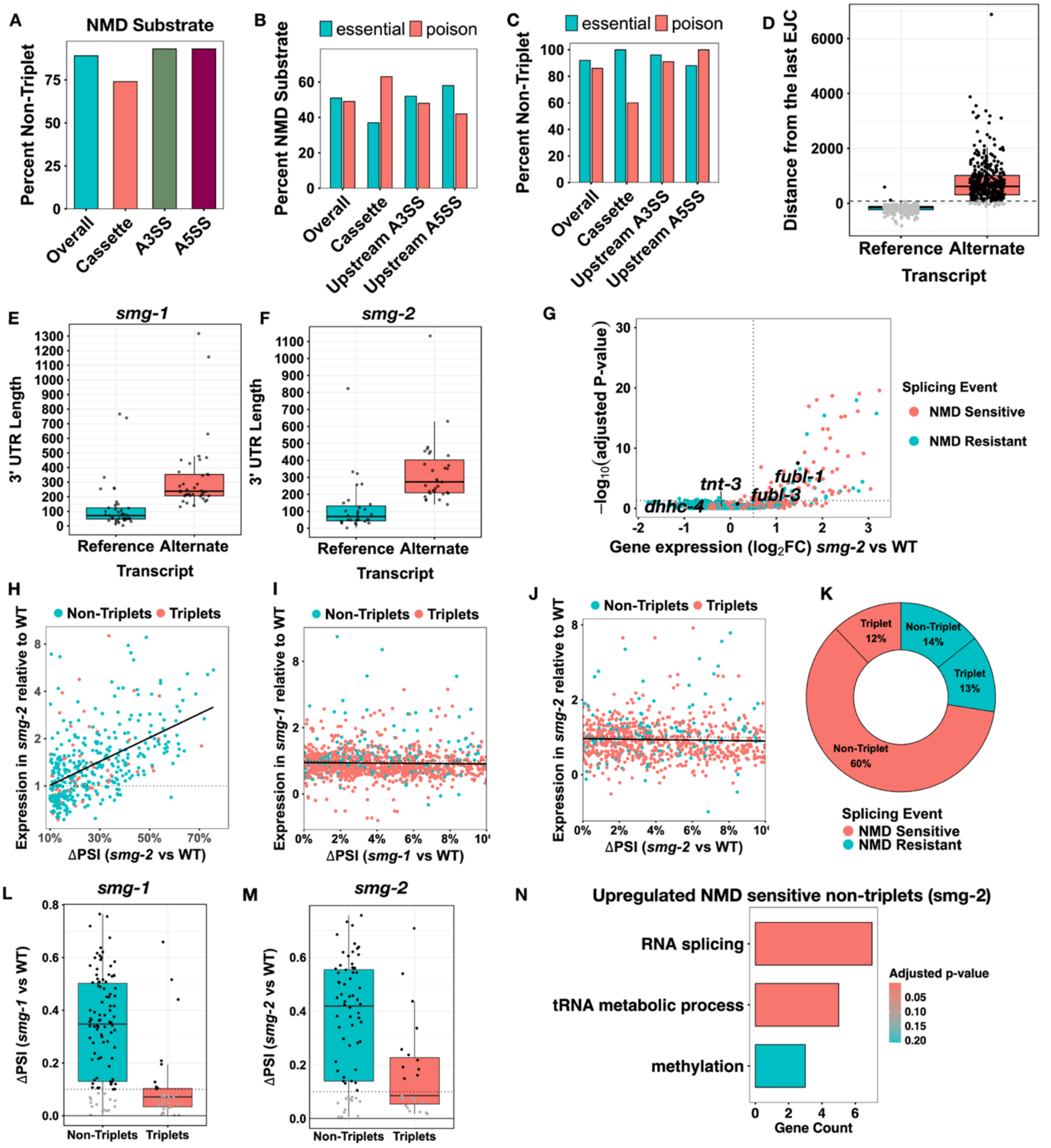

Figure S3: Non triplet splicing coupled with NMD affects transcript abundance. (A) Histogram showing the proportion of non-triplets among the NMD substrates in *smg-2.* (B) Histogram quantifying the contribution of essential and poison exons, Upstream alternative 3' and 5' splice sites in generating NMD substrates in *smg-2*. (C) Proportion of non-triplets among essential and poison exons, alternative 3' and 5' splice sites in *smg-2*. (D) Box plot depicting the distance between the stop codon and last exon junction complex for the reference and alternate transcript in *smg-2* for the non-triplets NMD sensitive category. Box plot of the 3'UTR length for reference and alternate transcript which are non-triplet NMD sensitive but violate the 55-nucleotide rule in *smg-1* (E) and *smg-2* (F). (G) Volcano plot of significant differentially expressed and alternatively spliced genes in *smg-2*. (H) Scatter plot showing correlation between change in PSI and expression in *smg-2* relative to wild type for NMD sensitive group. (I) Scatter plot showing correlation between change in PSI and expression in *smg-1* relative to wild type for NMD resistant group

(spearman correlation = -0.059). (J) Scatter plot showing correlation between change in PSI and expression in *smg-2* relative to wild type for NMD resistant group (spearman correlation = -0.078). (K) Distribution of significantly upregulated expressed genes among NMD resistant and sensitive categories in *smg-2*. (L) Box plot for the distribution of change in PSI among triplets and non-triplets in *smg-1* and *smg-2* (M). (N) Gene enrichment analysis of non-triplet upregulated NMD sensitive genes in *smg-2*, statistics were calculated using hypergeometric test (equivalent to a one-sided Fisher's exact test**)**, Benjamini Hochberg correction was applied to control the false discovery rate (FDR) for multiple comparisons, adjusted p-values < 0.05 were considered significant. The center line in the box plots (Figure D, E, F, L and M) show the median, with the bounds of the box representing the 25th and 75th percentiles (interquartile range). Whiskers extend to the minimum and maximum values within 1.5 × the interquartile range, points beyond the whiskers represent outliers.

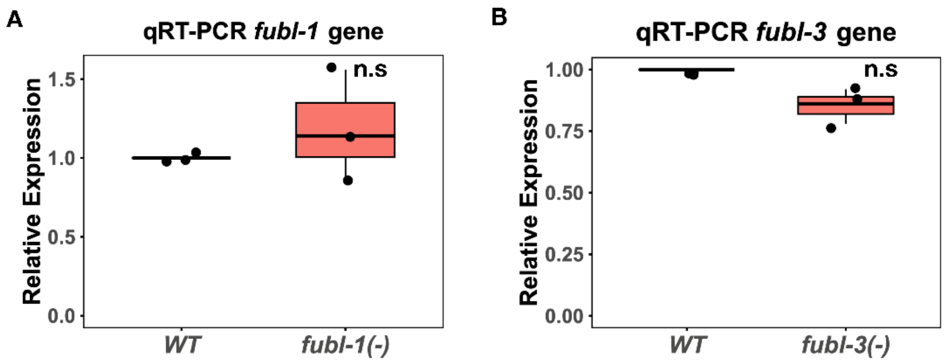

Figure S4: Expression of fubl-1 and fubl-3 in loss-of-function backgrounds. **(A)** Expression levels (in fold change) of *fubl-1* measured by quantitative RT–PCR in fubl-1 loss-of-function mutants relative to wild type.
(**B)** Expression levels of *fubl-3* measured by quantitative RT–PCR in fubl-3 loss-of-function mutants relative to wildtype. n.s (not significant) two sided paired t-test was performed. The center line in the box plots show the median, with the bounds of the box representing the 25th and 75th percentiles (interquartile range). Whiskers extend to the minimum and maximum values within 1.5 × the interquartile range, points beyond the whiskers represent outliers.

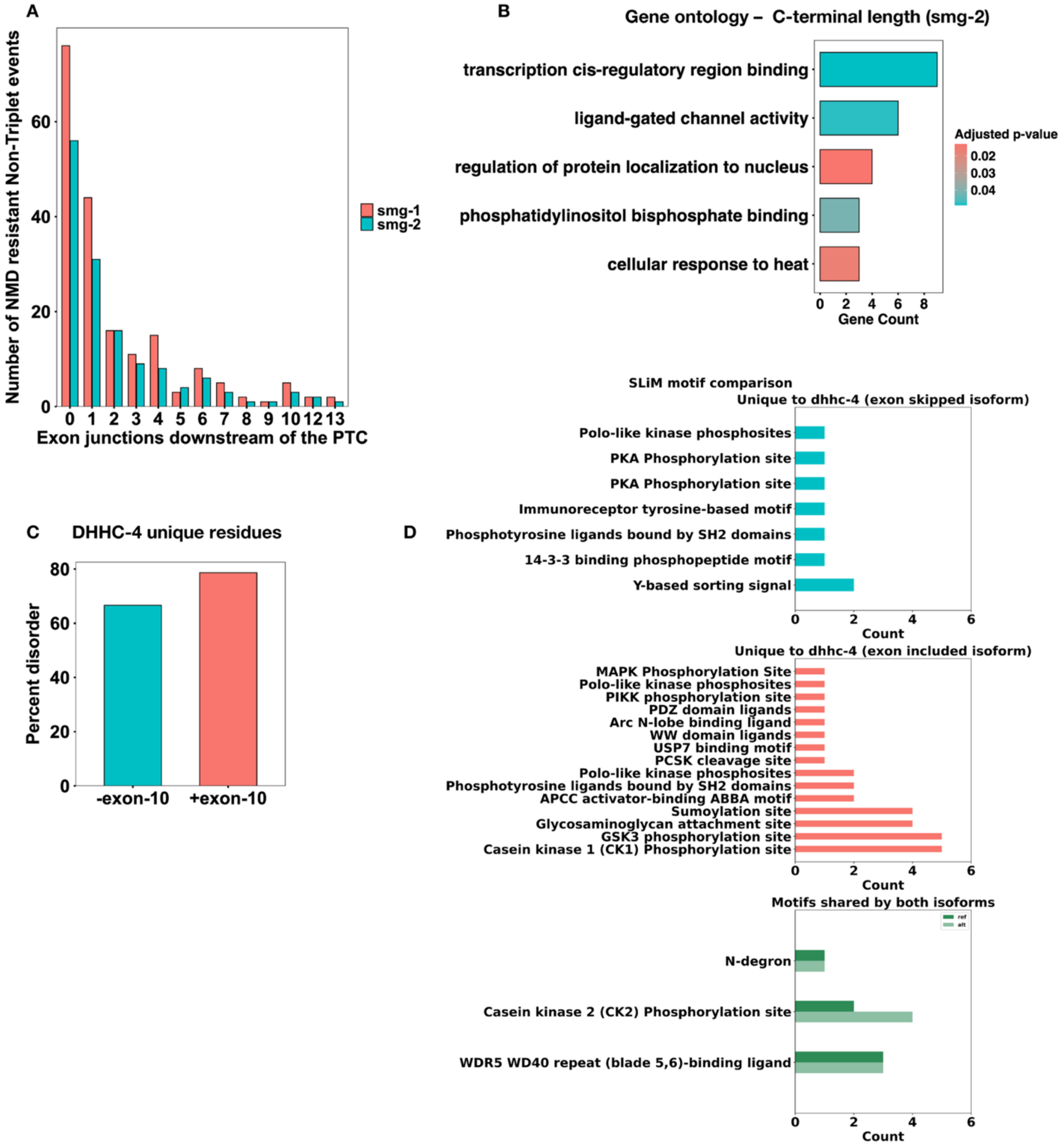

Figure S5: NMD resistant alternative splicing of non-triplets diversifies the transcriptome by shifting the reading frame. (A) Histogram of NMD resistant events plotted against number of exon junctions located downstream of the Premature Termination Codon in *smg-1* and *smg-2*. ((B) Gene ontology analysis for the C-terminal length category in *smg-2,* statistics were calculated using hypergeometric test (equivalent to a one-sided Fisher's exact test**)**, Benjamini Hochberg correction was applied to control the false discovery rate (FDR) for multiple comparisons, adjusted p-values < 0.05 were considered significant. (C) AIUPred predicted disorder scores for the unique amino acid residues in exon-10 skipped and included versions of DHHC-4 protein. (D) Eukaryotic Linear Motif (ELM) SLiM analysis for the unique C terminal residues of the DHHC-4 exon included and skipped isoform.

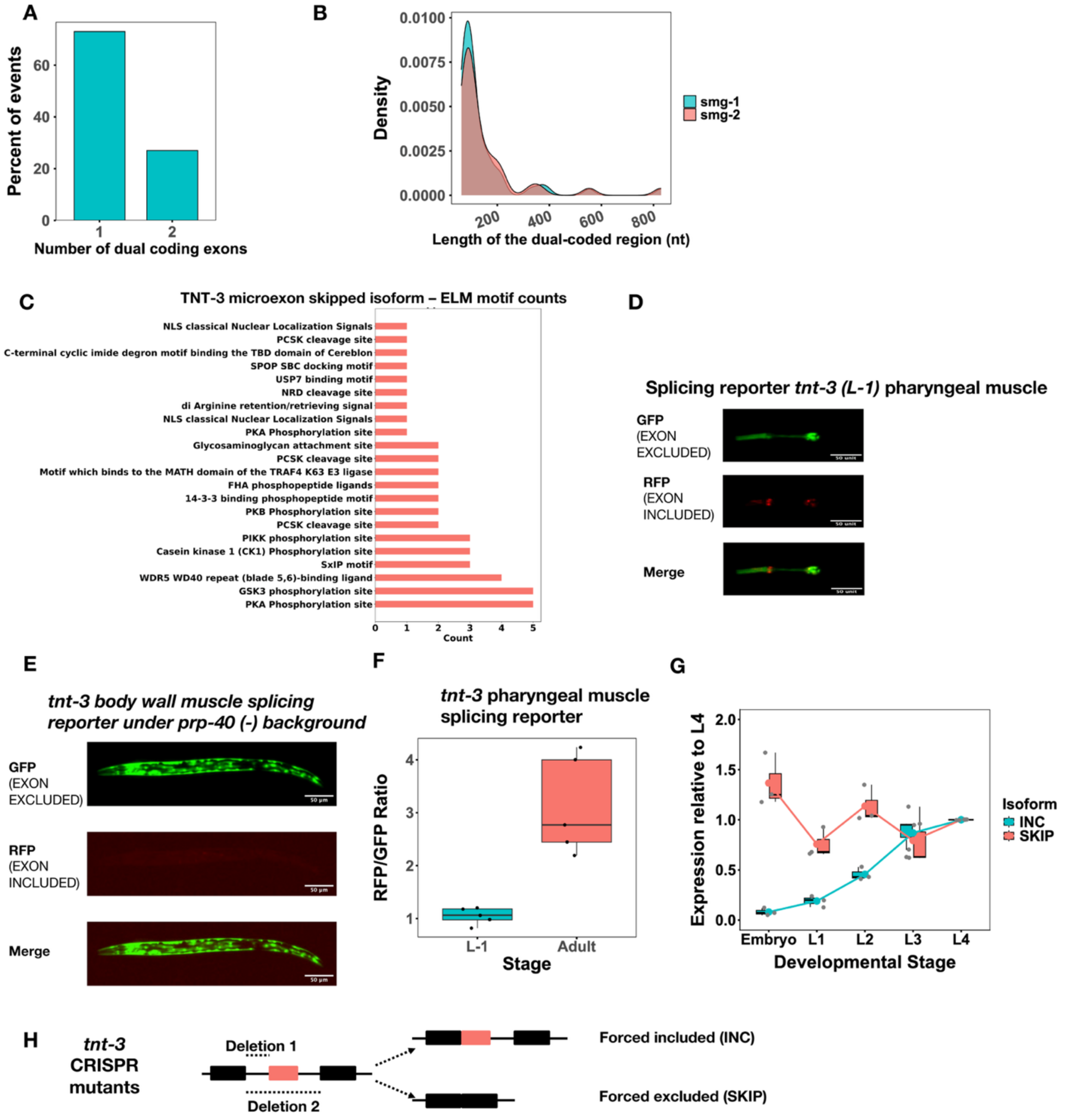

Figure S6: Non triplet alternative splicing gives rises to dual coding isoforms. (A) Histogram for the number of dual coding exons in *smg-2*. (B) Density plot of the dual coded region length in smg-1 and smg-2. (C) ELM SLiM motif analysis for the unique frameshifted residues of the TNT-3 microexon included isoform. (D) Splicing reporter images for the tnt-3 microexon skipped and included minigenes under the myo-2 pharyngeal muscle promoter (scale bar = 50 µm), representative image shown from five independent experiments with similar results. (E) Splicing reporter images for the tnt-3 microexon under the myo-3 body wall muscle promoter in *prp-40* null background (scale bar = 50 µm), representative image shown from three independent experiments with similar results, refer Figure 7H for wild type image. (F) Quantification of the RFP/GFP ratio (expressed as Percent spliced in) from the splicing reporter expressed in *myo-2* pharyngeal muscles of L1 larvae and adult animals (n = 5 worms in each stage), The center line in the box

plots show the median, with the bounds of the box representing the 25th and 75th percentiles (interquartile range). Whiskers extend to the minimum and maximum values within 1.5 × the interquartile range, points beyond the whiskers represent outliers (G) Isoform specific qRT-PCR for the *tnt-3* skipped (SKIP) and included transcript (INC) in wild type worms across various stages of development, expression was normalized to the corresponding L4 level for each isoform. Three independent biological replicates were analyzed per stage per isoform (n = 3). Points represent individual replicates; lines connect mean values across stages. (I) Generation of CRISPR tnt-3 forced included (INC) and forced skipped (SKIP) mutant.

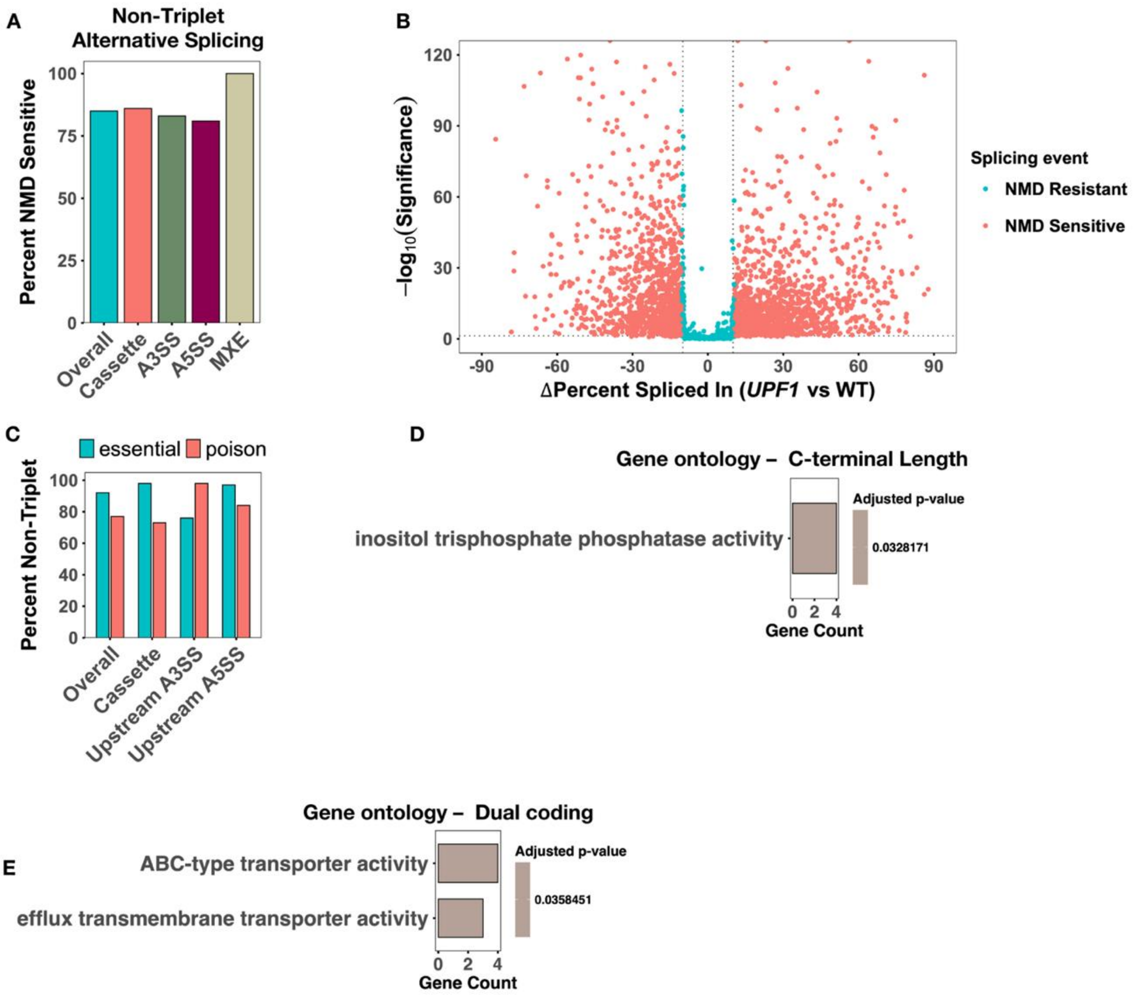

Figure S7: Non-triplet splicing outcomes in human transcriptome. (A) Histogram quantifying percentage of non-triplet alternative splicing events that are NMD sensitive (>|10%| change in PSI between wild type and *UPF1* knockdown cell line). (B) Volcano plot of non-triplet alternatively spliced genes between wild-type and *UPF1 KD*, Negative and positive change in PSI values represent exon skipping and inclusion in *UPF1 KD* relative to wild type respectively, color coding is based on the NMD sensitivity of the splicing event. (C) Proportion of non-triplet essential and poison exons, upstream 3' and 5' splice sites among NMD substrates (a subset of NMD sensitive cases whose alternative splicing changes the stop codon position identified from our computational pipeline). (D) Gene ontology for the alternative C-terminal length category. (E) Gene ontology for the dual coding category, for D and E statistics were calculated using hypergeometric test (equivalent to a one-sided Fisher's exact test**)**, Benjamini Hochberg correction was applied to control the false discovery rate (FDR) for multiple comparisons, adjusted p-values < 0.05 were considered significant.
