# Supplementary material for: Pervasive non-triplet alternative splicing drives functional isoform diversity
Source: Nat Commun. 2026 Apr 10;17:5112. doi: 10.1038/s41467-026-71615-5 (PMC13247117; doi:10.1038/s41467-026-71615-5)
Supplement: Supplementary file 2 — Description of Additional Supplementary Files [file 41467_2026_71615_MOESM2_ESM.pdf]

Description of Additional Supplementary Files

Supplementary Data 1. The robustness of cfDNA fragmentomics in relation to gene expressions in blood and placental tissues at different sequencing depths.

Supplementary Data 2. The number of genes in eight groups.

Supplementary Data 3. The summary of whole-genome sequencing data for plasma cfDNA.

Supplementary Data 4. The enrichment of pathways, biological processes and human gene-disease associations on a gene set with differential TSS coverages between early-onset PE pregnancies and controls. P-values for functional enrichment were calculated using the accumulative hypergeometric test in Metascape. Q-values were adjusted using the Benjamini-Hochberg (BH) method for multiple comparisons.

Supplementary Data 5. The enrichment of pathways, biological processes and human gene-disease associations on a gene set with differential TSS scores between early-onset PE pregnancies and controls. P-values for functional enrichment were calculated using the accumulative hypergeometric test in Metascape. Q-values were adjusted using the Benjamini-Hochberg (BH) method for multiple comparisons.

Supplementary Data 6. The enrichment of pathways, biological processes and human gene-disease associations on a gene set with differential gini coefficients between early-onset PE pregnancies and controls. P-values for functional enrichment were calculated using the accumulative hypergeometric test in Metascape. Q-values were adjusted using the Benjamini-Hochberg (BH) method for multiple comparisons.

Supplementary Data 7. The enrichment of pathways, biological processes and human gene-disease associations on a gene set with differential TSS coverages between late-onset PE pregnancies and controls. P-values for functional enrichment were calculated using the accumulative hypergeometric test in Metascape. Q-values were adjusted using the Benjamini-Hochberg (BH) method for multiple comparisons.

Supplementary Data 8. The enrichment of pathways, biological processes and human gene-disease associations on a gene set with differential TSS scores between late-onset PE pregnancies and controls. P-values for functional enrichment were calculated using the accumulative hypergeometric test in Metascape. Q-values were adjusted using the Benjamini-Hochberg (BH) method for multiple comparisons.

Supplementary Data 9. The enrichment of pathways, biological processes and human gene-disease associations on a gene set with differential gini coefficients between late-onset PE pregnancies and controls. P-values for functional enrichment were calculated using the accumulative hypergeometric test in Metascape. Q-values were adjusted using the Benjamini-Hochberg (BH) method for multiple comparisons.

Supplementary Data 10. The importance of the 254 TSS coverages ranked by the Boruta algorithm for predicting early-onset PE.

Supplementary Data 11. The importance of the 27 TSS scores ranked by the Boruta algorithm for predicting early-onset PE.

Supplementary Data 12. The importance of the 666 Gini coefficients ranked by the Boruta algorithm for predicting early-onset PE.

Supplementary Data 13. The importance of the 33 TSS coverages ranked by the Boruta algorithm for predicting late-onset PE.

Supplementary Data 14. The importance of the 24 TSS scores ranked by the Boruta algorithm for predicting late-onset PE.

Supplementary Data 15. The importance of the 526 Gini coefficients ranked by the Boruta algorithm for predicting late-onset PE.

Supplementary Data 16. The performance of random forest models based on cfDNA fragmentomics in predicting early-onset PE.

Supplementary Data 17. The performance of random forest models based on cfDNA fragmentomics in predicting late-onset PE.

Supplementary Data 18. The performance of random forest models based on cfDNA fragmentomics in predicting early-onset PE across read depths greater than 10×.

Supplementary Data 19. The performance of random forest models based on cfDNA fragmentomics in predicting late-onset PE across read depths greater than 10×.

Supplementary Data 20. The pearson correlation between gestational age and cfDNA fragmentomics in early-onset PE pregnancies and controls.

Supplementary Data 21. The pearson correlation between gestational age and cfDNA fragmentomics in late-onset PE pregnancies and controls.

Supplementary Data 22. Comparison of predictive performance among models based on cfDNA fragmentomics, maternal risk factors, and combined models for predicting early-onset PE. The DeLong's test (two-sided) was used to compare area under the curves (AUCs) of two models.

Supplementary Data 23. Comparison of predictive performance among models based on cfDNA fragmentomics, maternal risk factors, and combined models for predicting late-onset PE. The DeLong's test (two-sided) was used to compare area under the curves (AUCs) of two models.
