# Supplementary material for: Pervasive non-triplet alternative splicing drives functional isoform diversity
Source: Nat Commun. 2026 Apr 10;17:5112. doi: 10.1038/s41467-026-71615-5 (PMC13247117; doi:10.1038/s41467-026-71615-5)
Supplement: Supplementary file 8 — Reporting Summary [file 41467_2026_71615_MOESM8_ESM.pdf]

nature portfolio

Corresponding author(s): Adam Norris

Last updated by author(s): Feb 14, 2026

# Reporting Summary

Nature Portfolio wishes to improve the reproducibility of the work that we publish. This form provides structure for consistency and transparency in reporting. For further information on Nature Portfolio policies, see our Editorial Policies and the Editorial Policy Checklist.

## Statistics

For all statistical analyses, confirm that the following items are present in the figure legend, table legend, main text, or Methods section.

| n/a | Confirmed | |
|---|---|---|
| ☐ | ☒ | The exact sample size ($n$) for each experimental group/condition, given as a discrete number and unit of measurement |
| ☐ | ☒ | A statement on whether measurements were taken from distinct samples or whether the same sample was measured repeatedly |
| ☐ | ☒ | The statistical test(s) used AND whether they are one- or two-sided<br>*Only common tests should be described solely by name; describe more complex techniques in the Methods section.* |
| ☒ | ☐ | A description of all covariates tested |
| ☐ | ☒ | A description of any assumptions or corrections, such as tests of normality and adjustment for multiple comparisons |
| ☐ | ☒ | A full description of the statistical parameters including central tendency (e.g. means) or other basic estimates (e.g. regression coefficient) AND variation (e.g. standard deviation) or associated estimates of uncertainty (e.g. confidence intervals) |
| ☐ | ☒ | For null hypothesis testing, the test statistic (e.g. $F$, $t$, $r$) with confidence intervals, effect sizes, degrees of freedom and $P$ value noted<br>*Give P values as exact values whenever suitable.* |
| ☒ | ☐ | For Bayesian analysis, information on the choice of priors and Markov chain Monte Carlo settings |
| ☒ | ☐ | For hierarchical and complex designs, identification of the appropriate level for tests and full reporting of outcomes |
| ☐ | ☒ | Estimates of effect sizes (e.g. Cohen's $d$, Pearson's $r$), indicating how they were calculated |

*Our web collection on statistics for biologists contains articles on many of the points above.*

## Software and code

Policy information about availability of computer code

| | |
|---|---|
| Data collection | No software used in the collection of the data |
| Data analysis | Junction Usage Model (2.0.2) (JUM)<br>STAR (2.0)<br>MAJIQ (2.5.7)<br>Custom scripts available at https://github.com/shameerudeen/Non-Triplet-splicing |

For manuscripts utilizing custom algorithms or software that are central to the research but not yet described in published literature, software must be made available to editors and reviewers. We strongly encourage code deposition in a community repository (e.g. GitHub). See the Nature Portfolio guidelines for submitting code & software for further information.

## Data

Policy information about availability of data

All manuscripts must include a data availability statement. This statement should provide the following information, where applicable:

- Accession codes, unique identifiers, or web links for publicly available datasets
- A description of any restrictions on data availability
- For clinical datasets or third party data, please ensure that the statement adheres to our policy

The RNA-Seq data generated in this study have been deposited in the NCBI GEO database under accession code GSE313026 [https://www.ncbi.nlm.nih.gov/geo/

query/acc.cgi?acc=GSE101099]. The human RNA-Seq data re-analyzed for this study have been deposited in the European Nucleotide Archive under the accession code E-MTAB-13787 [www.ebi.ac.uk/biostudies/ArrayExpress/studies/E-MTAB-13787]. Source Data are provided with this paper.

## Research involving human participants, their data, or biological material

Policy information about studies with human participants or human data. See also policy information about sex, gender (identity/presentation), and sexual orientation and race, ethnicity and racism.

| | |
|---|---|
| Reporting on sex and gender | n/a |
| Reporting on race, ethnicity, or other socially relevant groupings | n/a |
| Population characteristics | n/a |
| Recruitment | n/a |
| Ethics oversight | n/a |

Note that full information on the approval of the study protocol must also be provided in the manuscript.

# Field-specific reporting

Please select the one below that is the best fit for your research. If you are not sure, read the appropriate sections before making your selection.

[x] Life sciences [ ] Behavioural & social sciences [ ] Ecological, evolutionary & environmental sciences

For a reference copy of the document with all sections, see nature.com/documents/nr-reporting-summary-flat.pdf

# Life sciences study design

All studies must disclose on these points even when the disclosure is negative.

| | |
|---|---|
| Sample size | Our RNA-Seq studies used a standard in the field of three biological replicates per strain. No sample size calculation was performed. Three biological replicates, at sequencing depths greater than 10 million reads per sample, have been empirically determined to give satisfactory statistical power in alternative splicing studies. |
| Data exclusions | None |
| Replication | Independent biological replicates used (3 for each condition). All attempts at replication were successful |
| Randomization | Randomization not relevant to this study: global gene expression analysis was used on every cell from every organism in the population |
| Blinding | Blinding was not used. Blinding was not relevant to our studies (e.g. RNA Seq data are not subjective values, nor are the computational pipelines or RT-PCR results) |

# Reporting for specific materials, systems and methods

We require information from authors about some types of materials, experimental systems and methods used in many studies. Here, indicate whether each material, system or method listed is relevant to your study. If you are not sure if a list item applies to your research, read the appropriate section before selecting a response.

### Materials & experimental systems

| n/a | Involved in the study | |
|---|---|---|
| [x] | [ ] | Antibodies |
| [x] | [ ] | Eukaryotic cell lines |
| [x] | [ ] | Palaeontology and archaeology |
| [ ] | [x] | Animals and other organisms |
| [x] | [ ] | Clinical data |
| [x] | [ ] | Dual use research of concern |
| [x] | [ ] | Plants |

### Methods

| n/a | Involved in the study | |
|---|---|---|
| [x] | [ ] | ChIP-seq |
| [x] | [ ] | Flow cytometry |
| [x] | [ ] | MRI-based neuroimaging |

# Animals and other research organisms

Policy information about studies involving animals; ARRIVE guidelines recommended for reporting animal research, and Sex and Gender in Research

| | |
|---|---|
| Laboratory animals | Caenorhabditis elegans, N2 strain, L4 (4th larval) stage, hermaphrodite, available from https://cgc.umn.edu/strain/CGC1 |
| Wild animals | Study did not involve wild animals |
| Reporting on sex | All animals used were hermaphroditic |
| Field-collected samples | Study did not involve field-collected samples |
| Ethics oversight | No ethical oversight was required for invetebrate animal use |

Note that full information on the approval of the study protocol must also be provided in the manuscript.

# Plants

| | |
|---|---|
| Seed stocks | n/a |
| Novel plant genotypes | n/a |
| Authentication | n/a |
