# Supplementary material for: Pervasive non-triplet alternative splicing drives functional isoform diversity
Source: Nat Commun. 2026 Apr 10;17:5112. doi: 10.1038/s41467-026-71615-5 (PMC13247117; doi:10.1038/s41467-026-71615-5)
Supplement: Supplementary file 10 — Source Data [file 41467_2026_71615_MOESM10_ESM.zip › source data/raw_gel.pdf]

Figure 3E

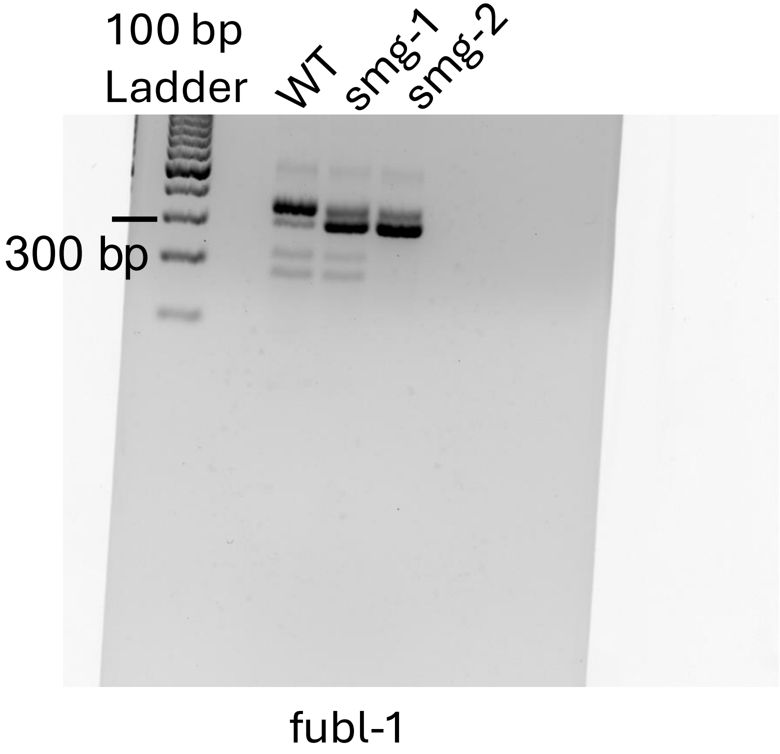
100 bp
Ladder
WT
smg-1
smg-2
300 bp
fubl-1
100 bp
Ladder
WT
smg-1
smg-2
200 bp
fubl-3

Figure 3E

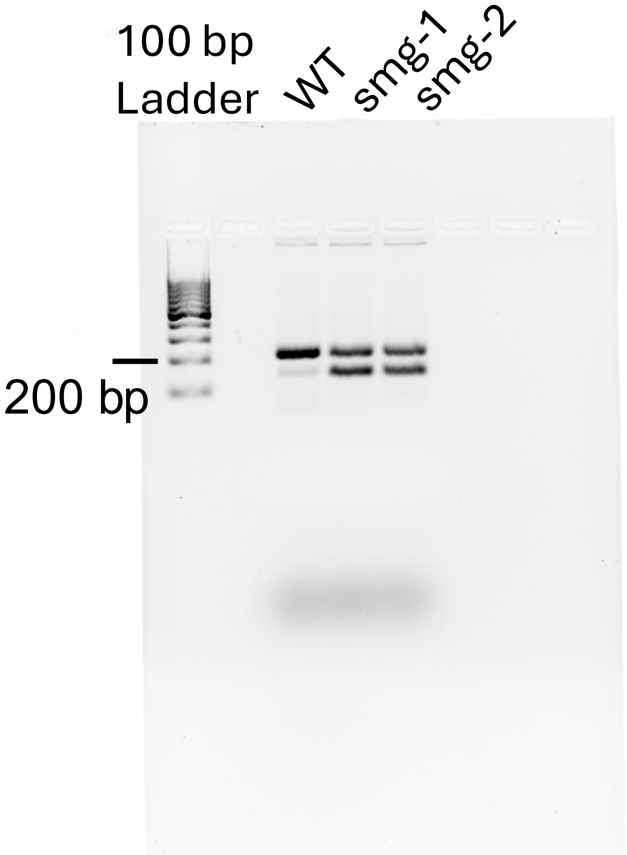

sfa-1

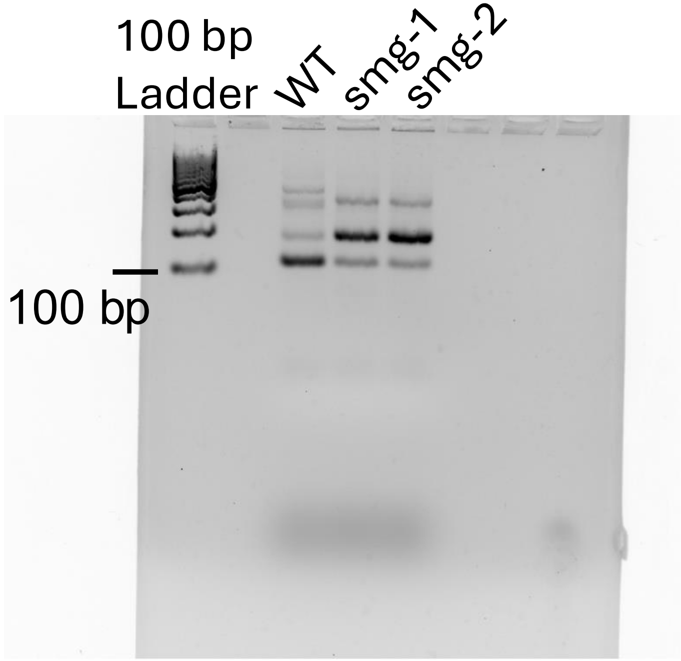

rsp-7

Figure 3E

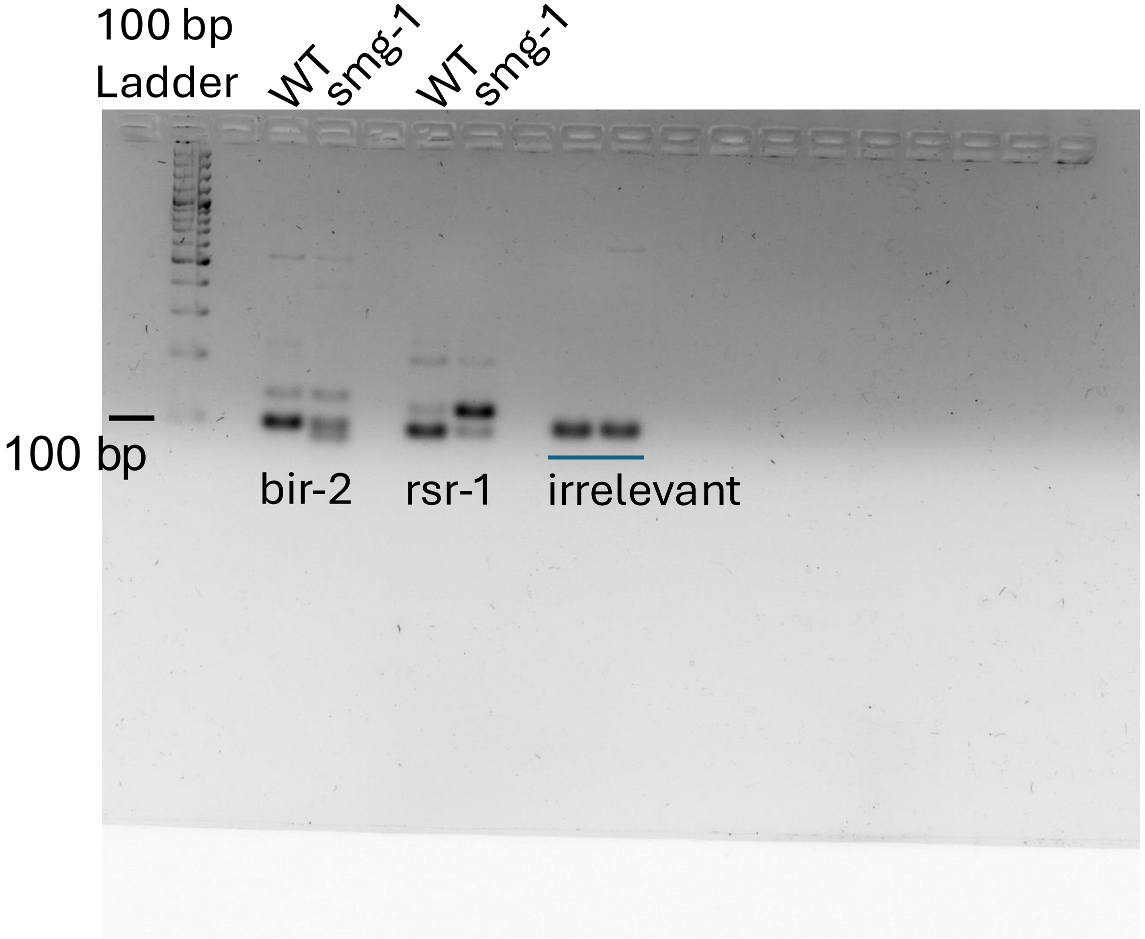

Figure 3E

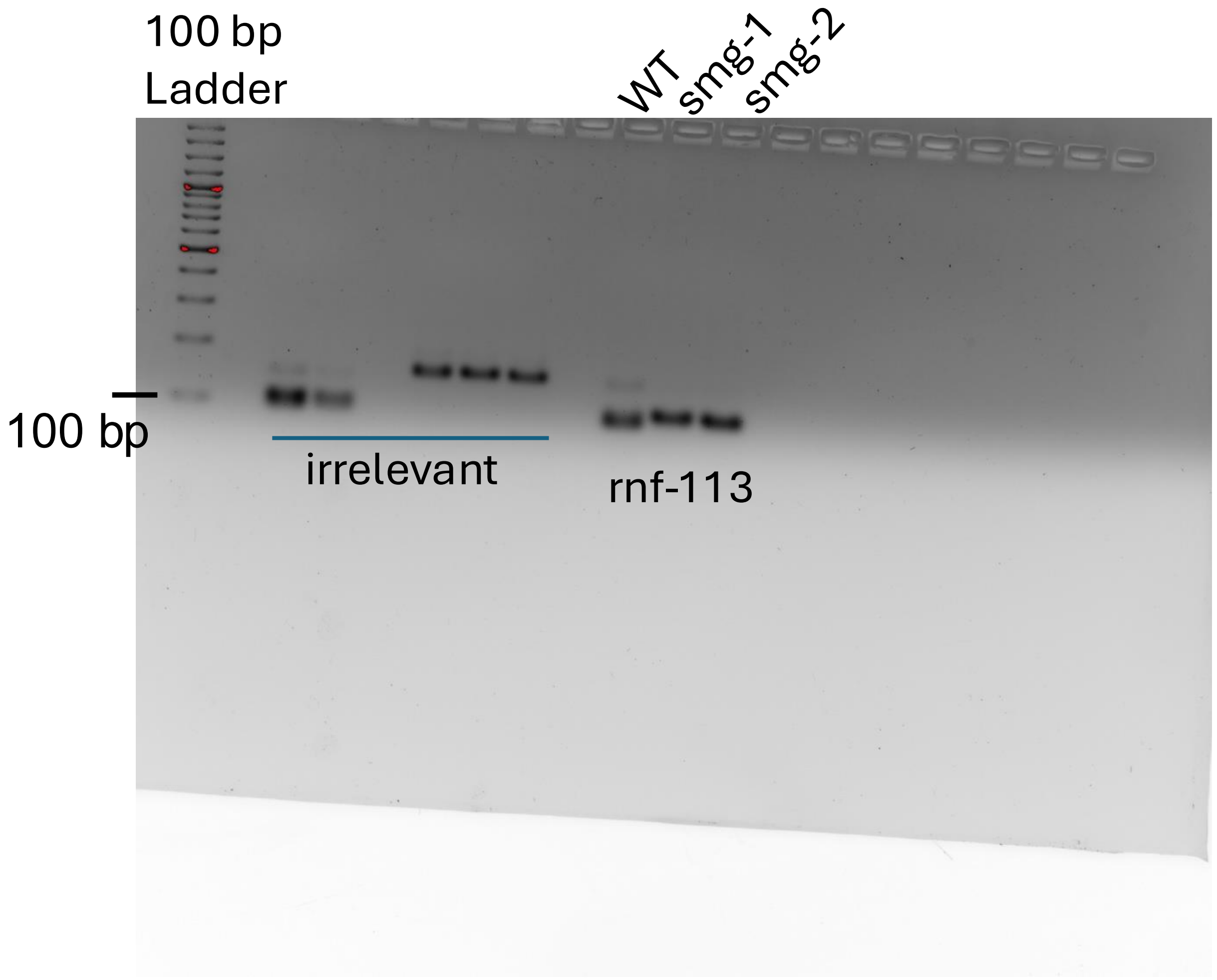

rnf-113

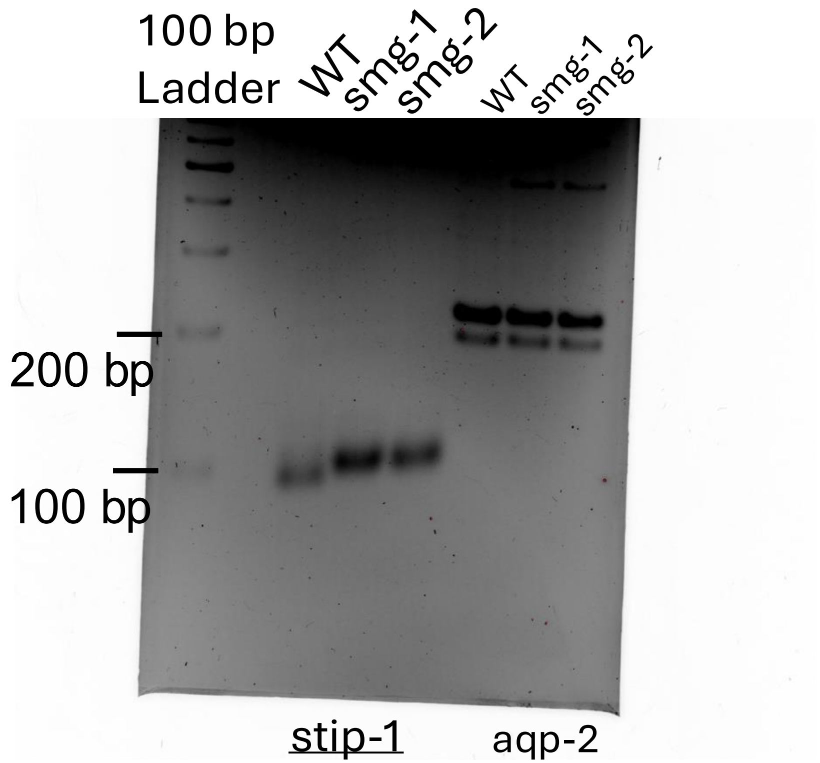

Figure 4F

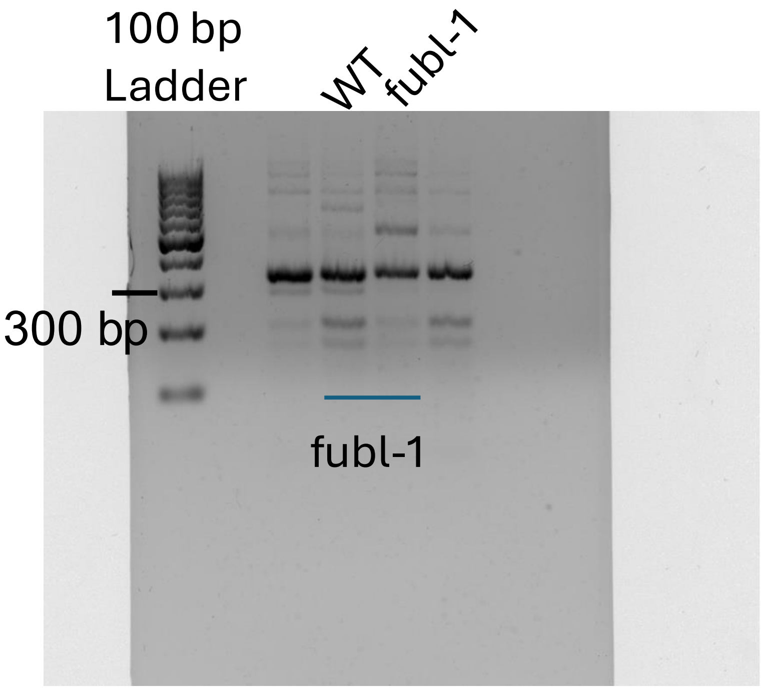

Figure 4G

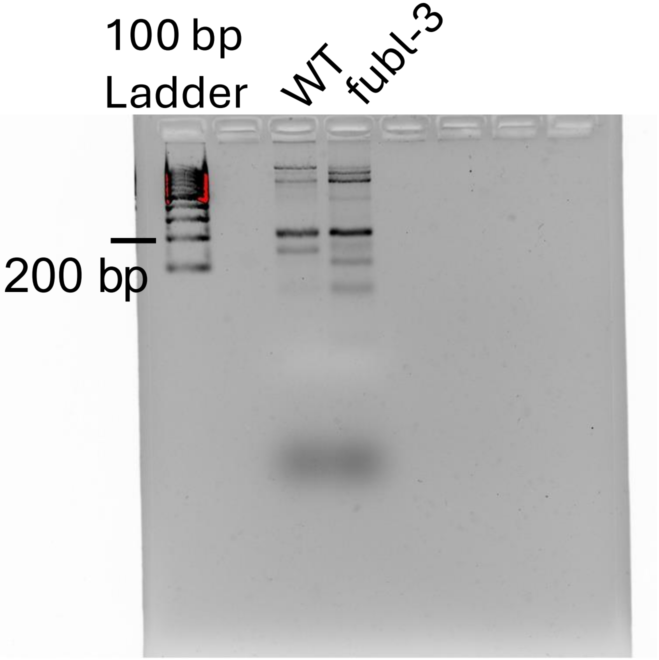

Figure 4M

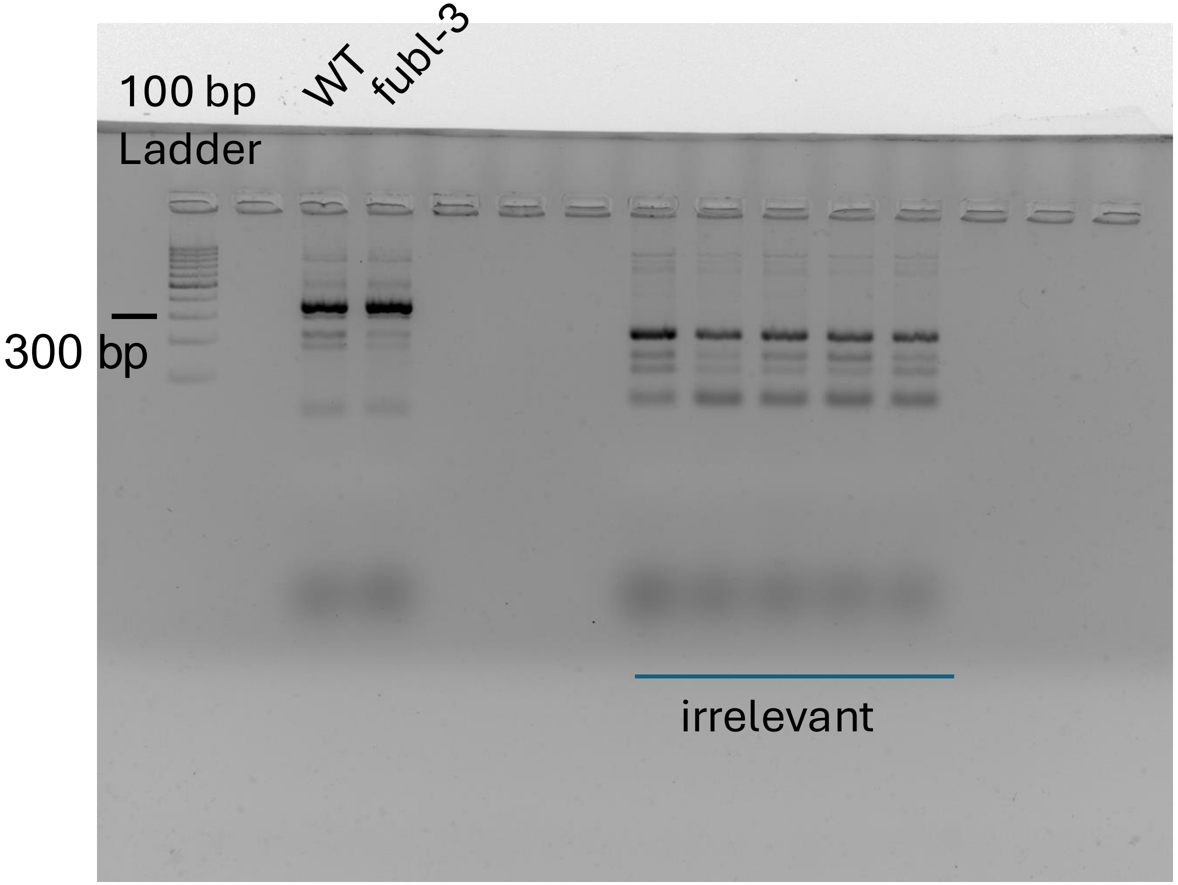

Figure 4N

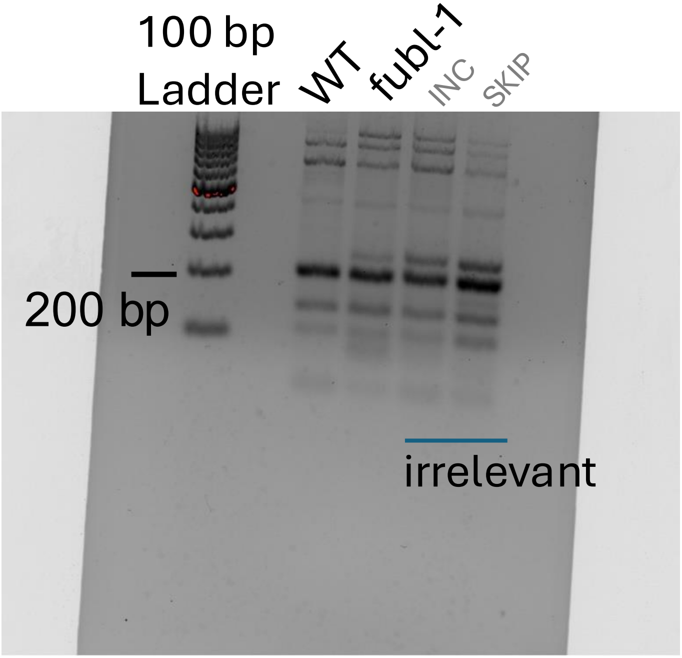

Figure 6A

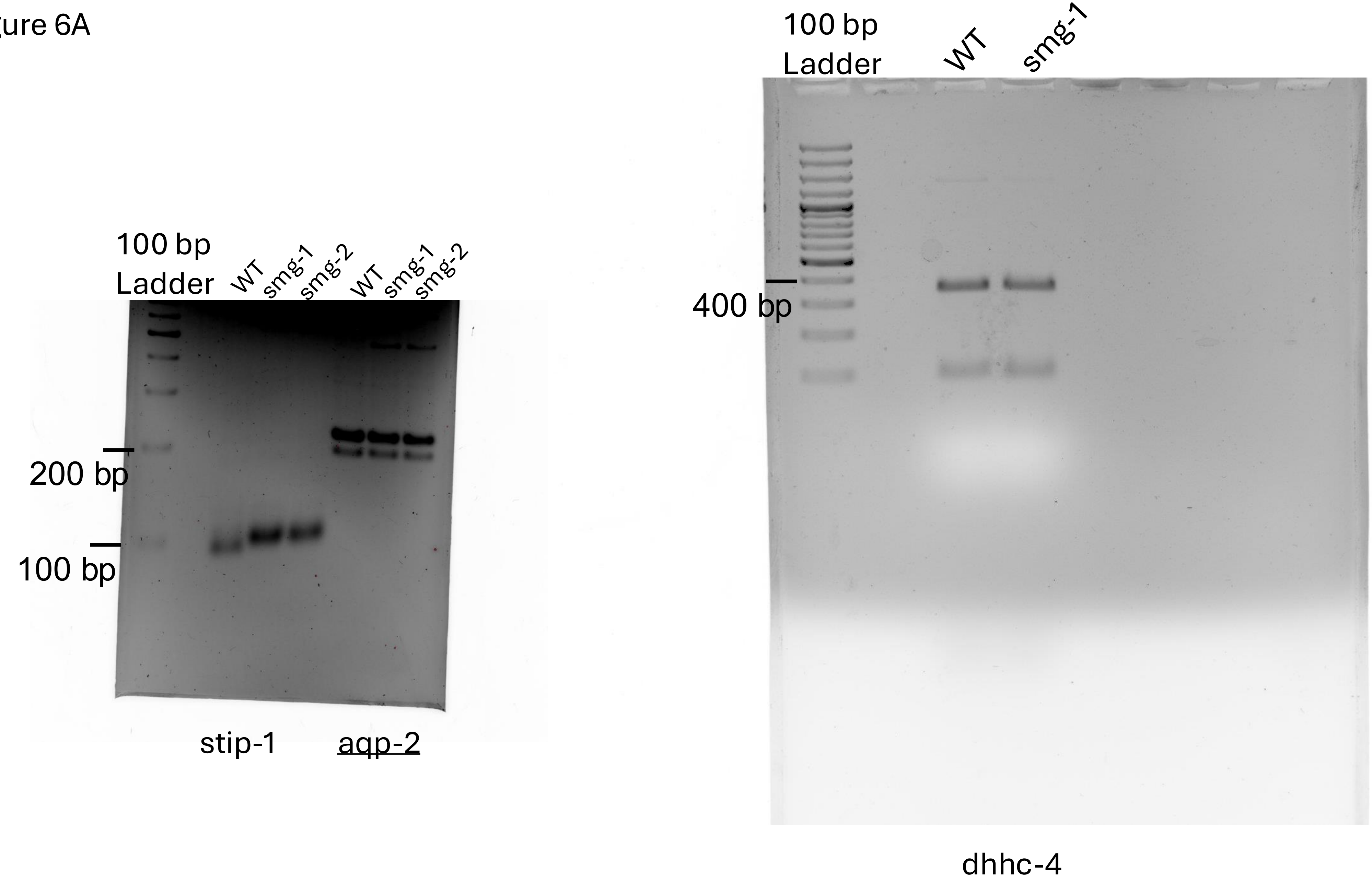

100 bp
Ladder
WT
smg-1
smg-2
WT
smg-1
smg-2
200 bp
100 bp
stip-1
aqp-2
100 bp
Ladder
WT
smg-1
400 bp
dhhc-4

Figure 6A

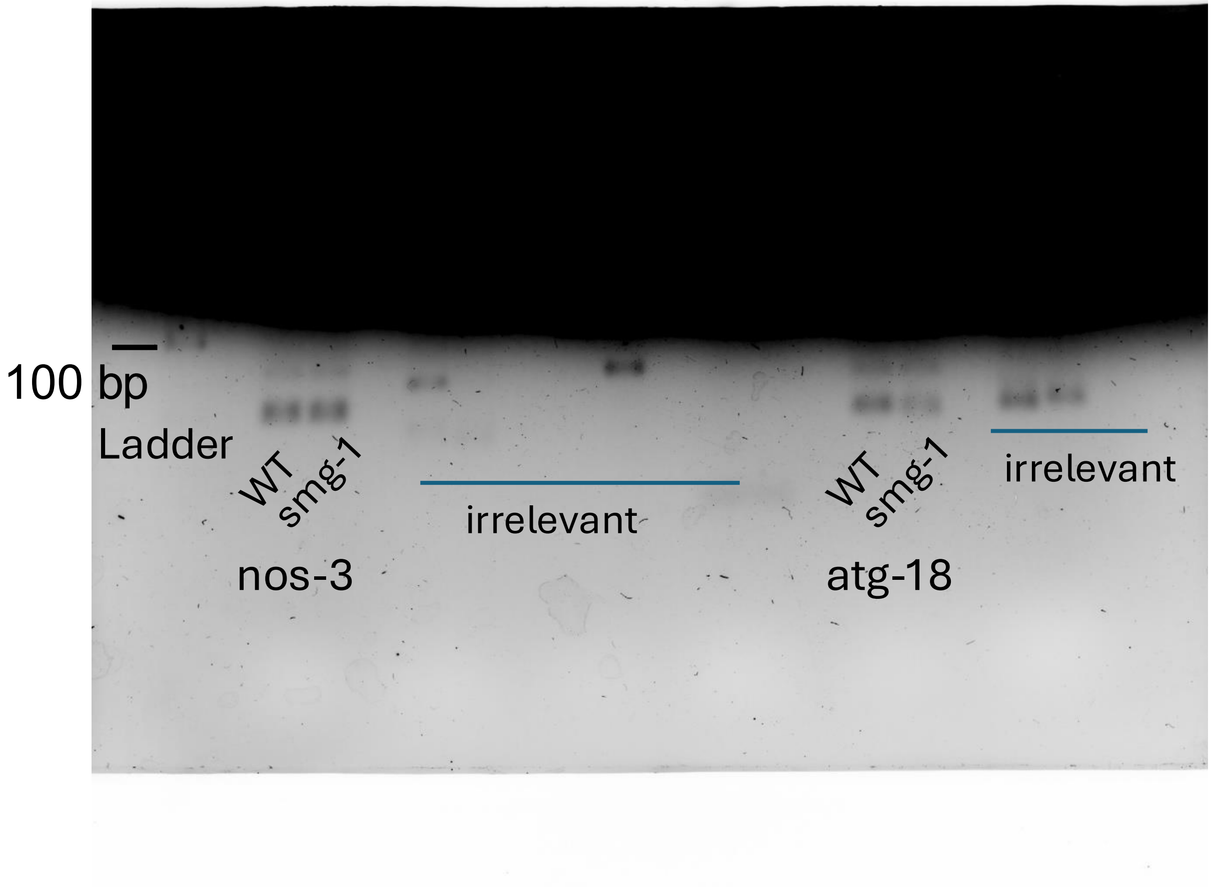
100 bp
Ladder
WT
smg-1
nos-3
irrelevant
WT
smg-1
atg-18
irrelevant

Figure 7- A

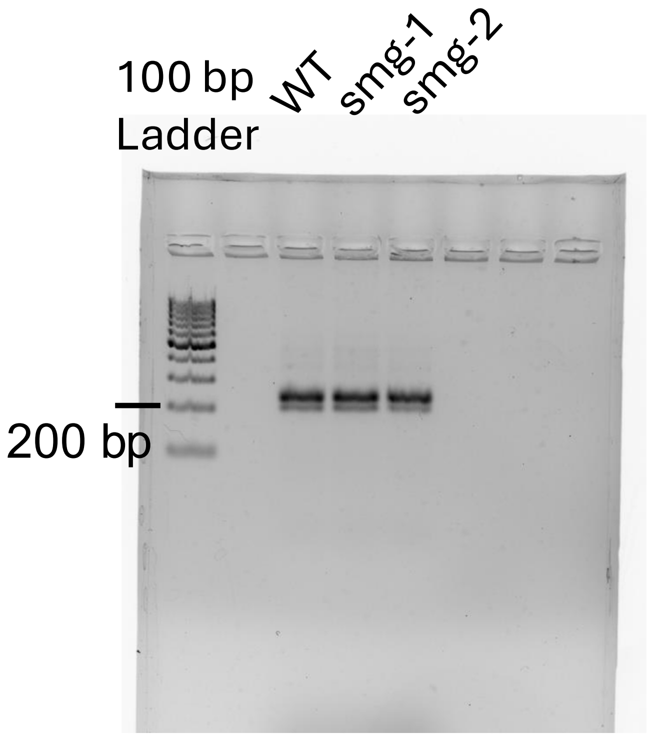

tnt-3

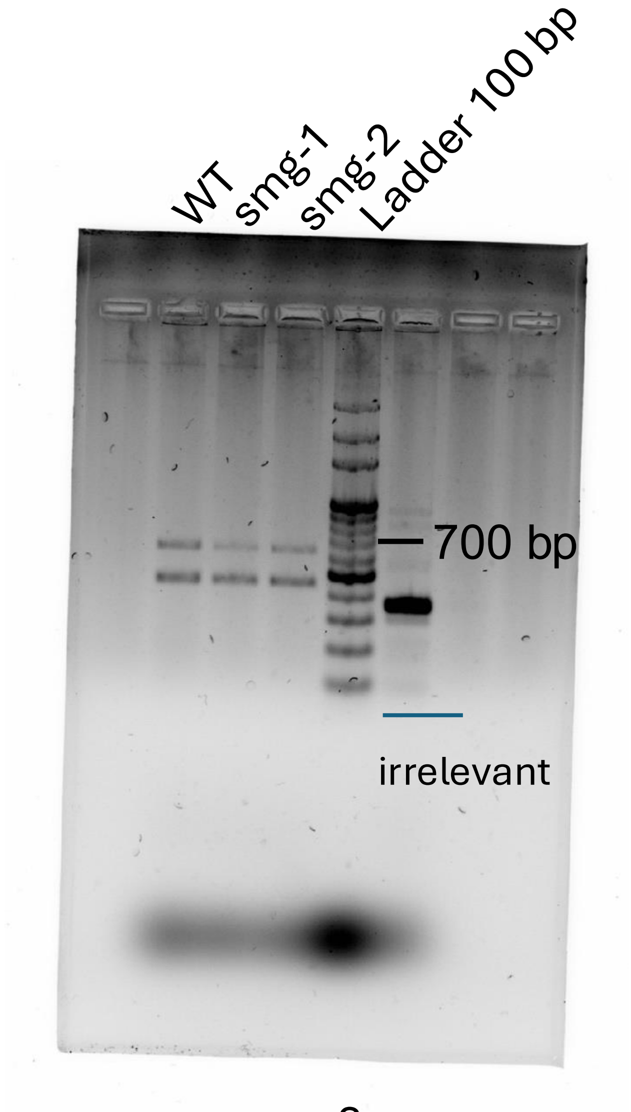

par-3

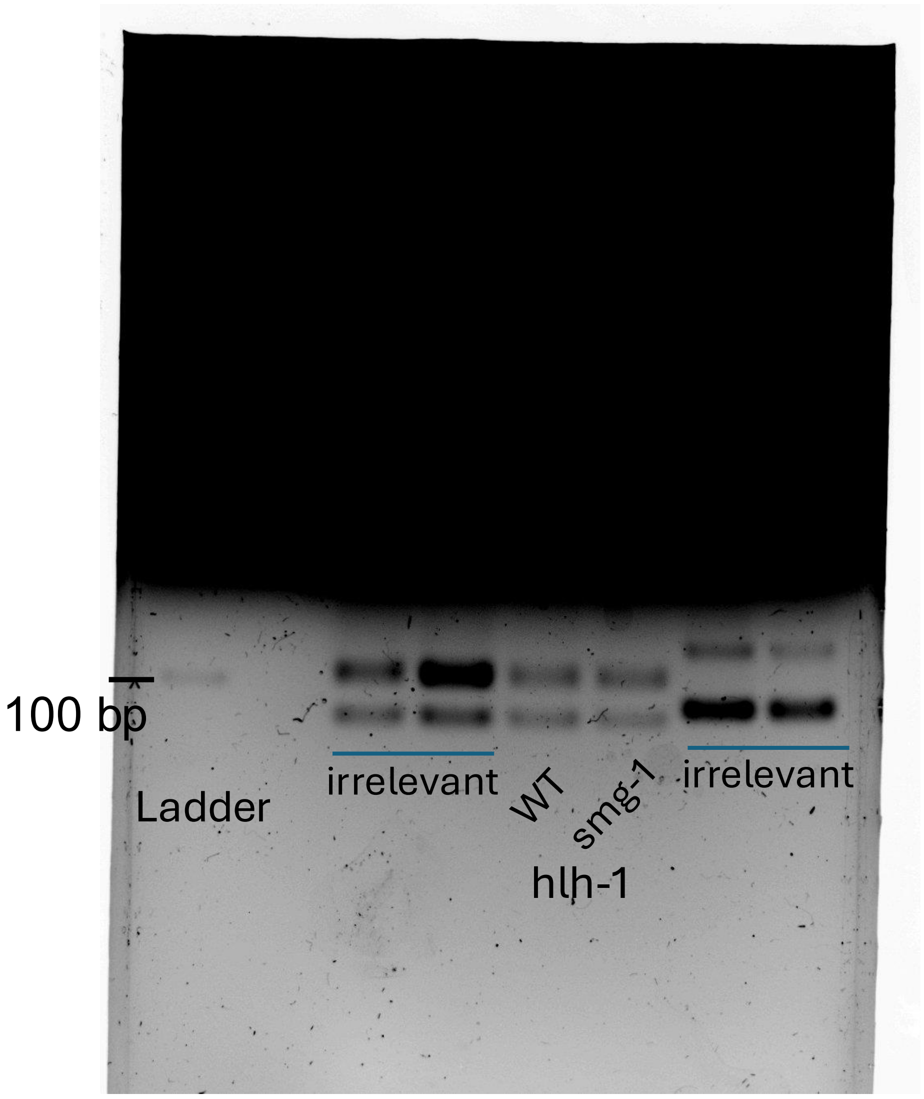

hlh-1

Figure 7- I & J

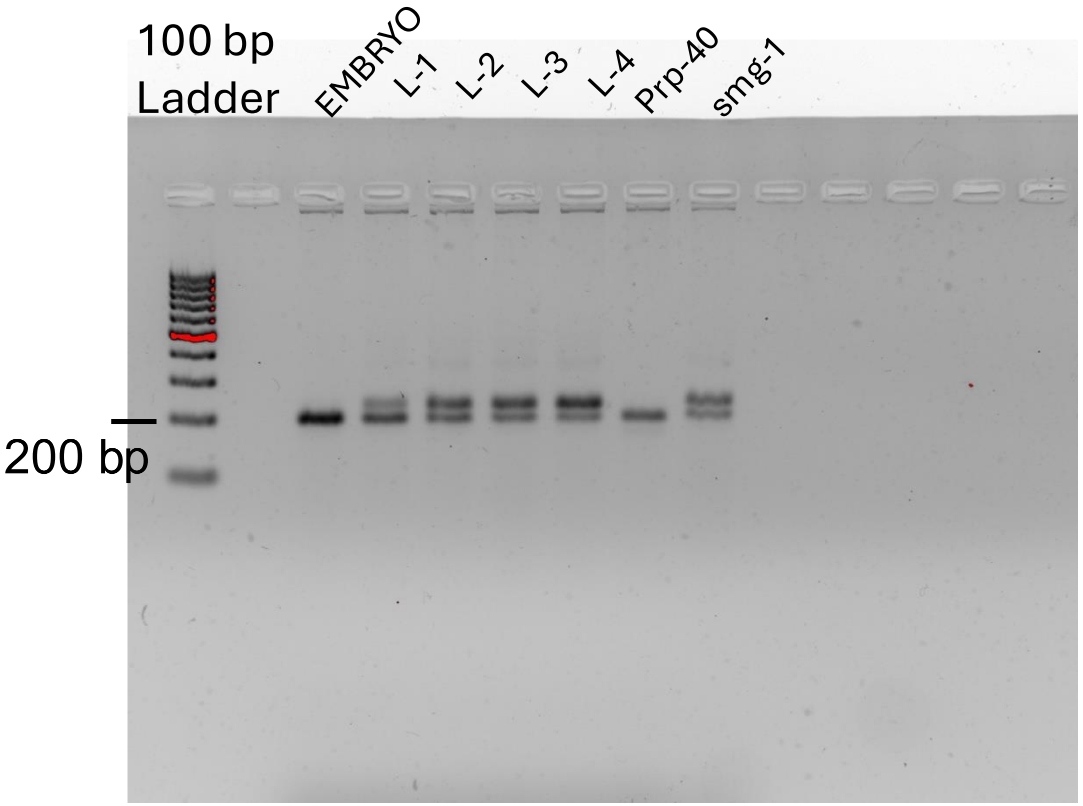

tnt-3
