# Supplementary figures and images for: Pervasive non-triplet alternative splicing drives functional isoform diversity
Source: Nat Commun. 2026 Apr 10;17:5112. doi: 10.1038/s41467-026-71615-5 (PMC13247117; doi:10.1038/s41467-026-71615-5)

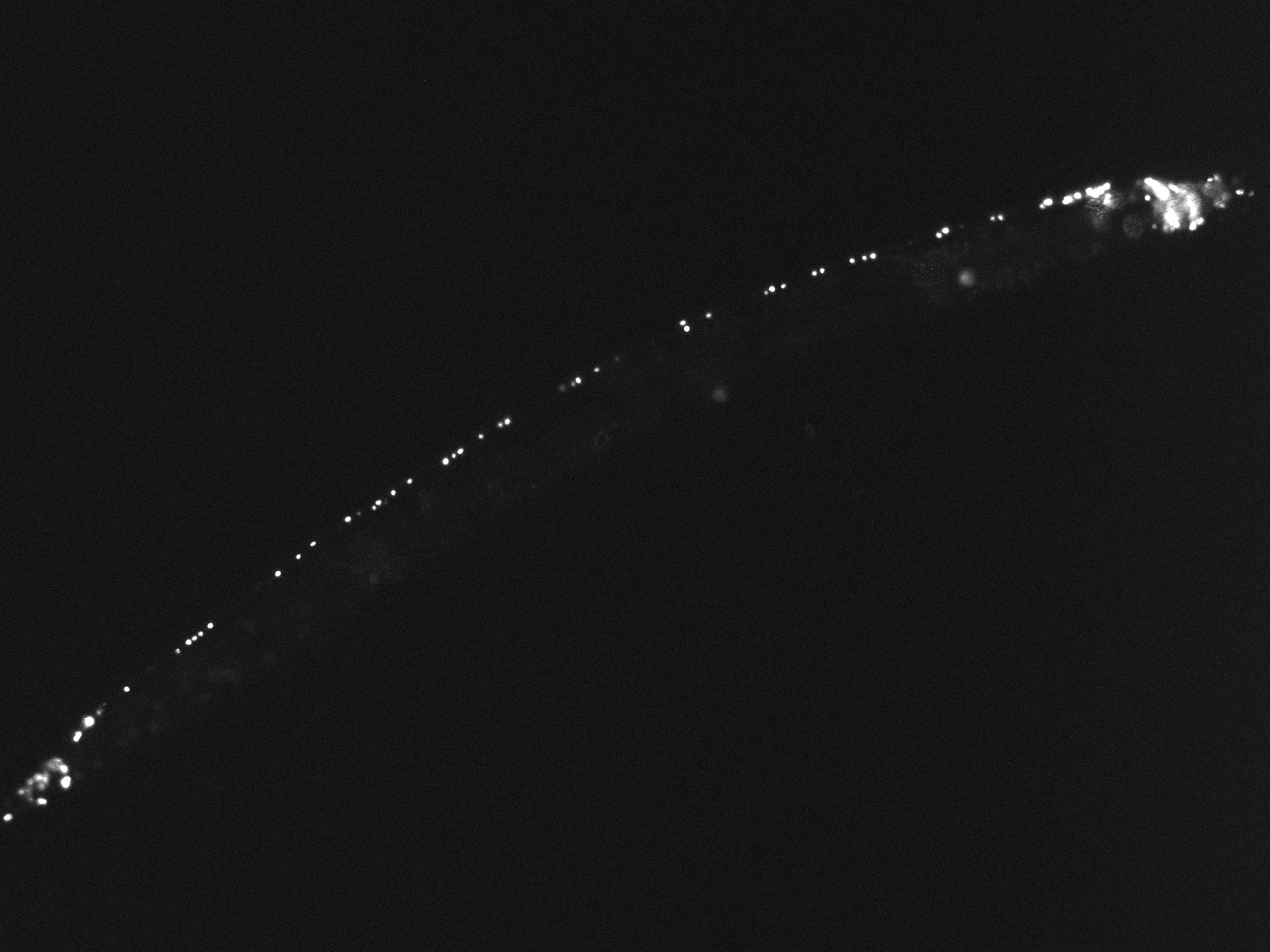

Supplement: Supplementary file 10 — Source Data [file 41467_2026_71615_MOESM10_ESM.zip › source data/microscopy_raw_images/FIGURE-6I/6I_RFP.tif]

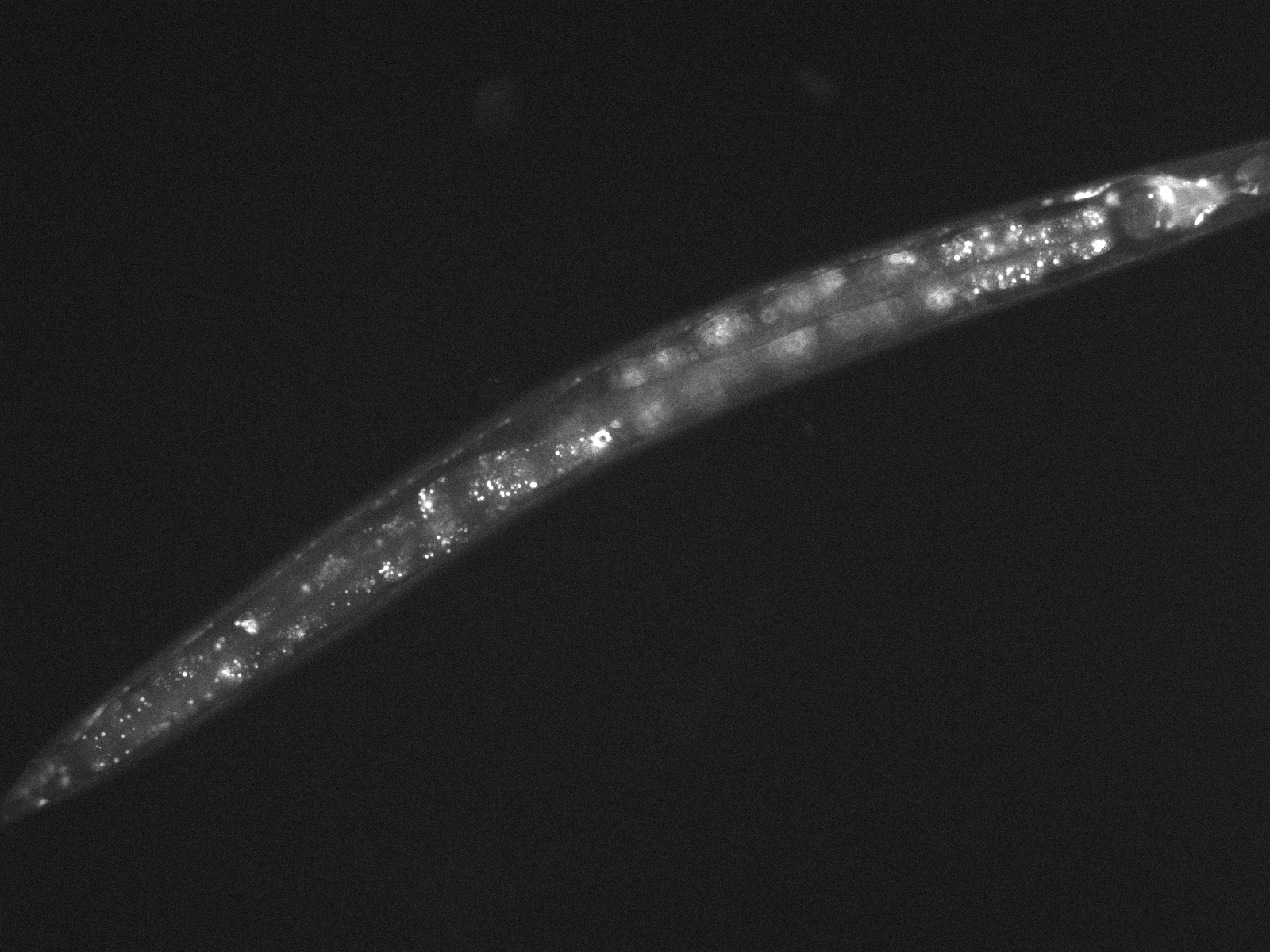

Supplement: Supplementary file 10 — Source Data [file 41467_2026_71615_MOESM10_ESM.zip › source data/microscopy_raw_images/FIGURE-6I/6I_GFP.tif]

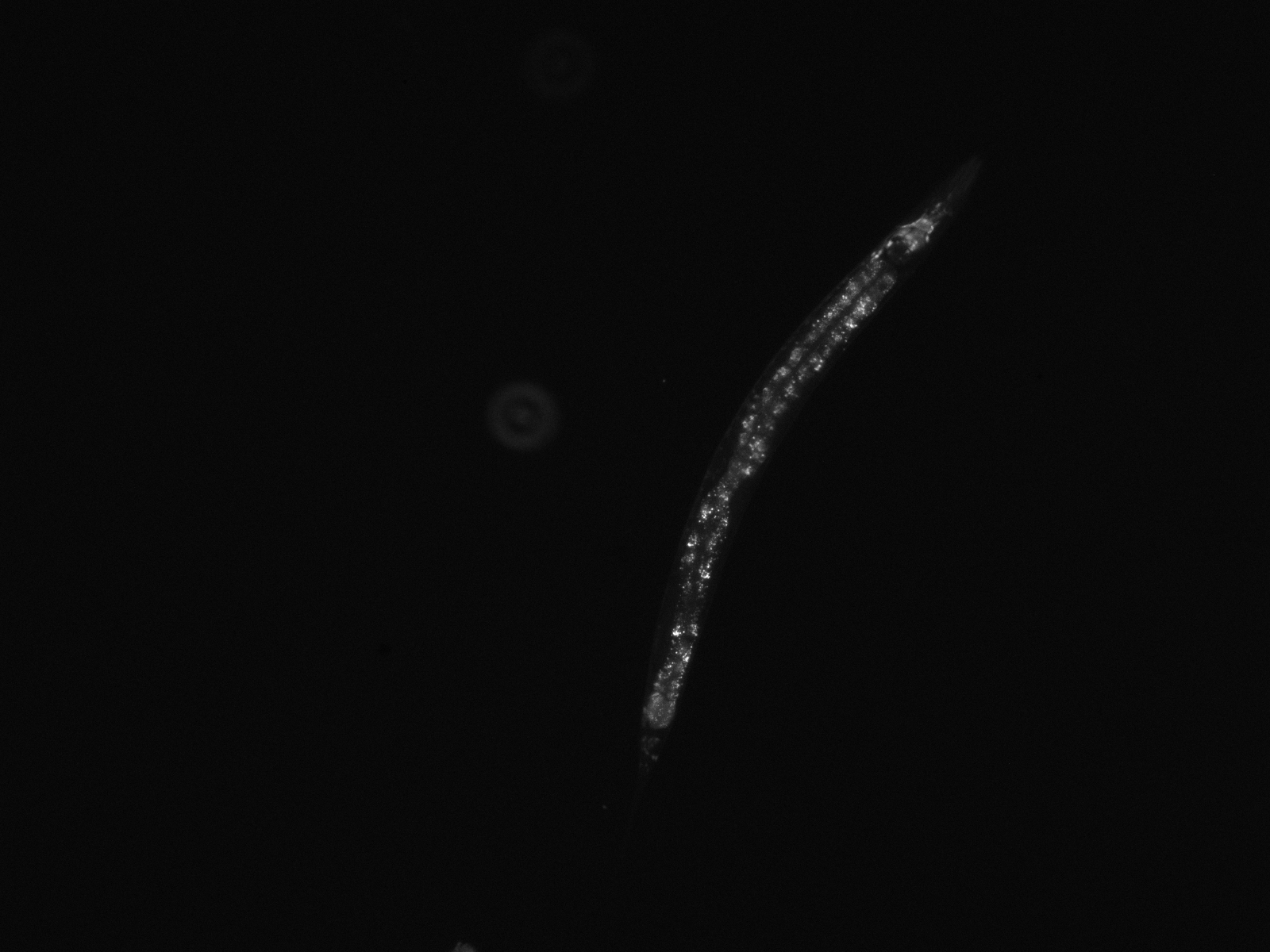

Supplement: Supplementary file 10 — Source Data [file 41467_2026_71615_MOESM10_ESM.zip › source data/microscopy_raw_images/FIGURE-6G/6G_GFP.tif]

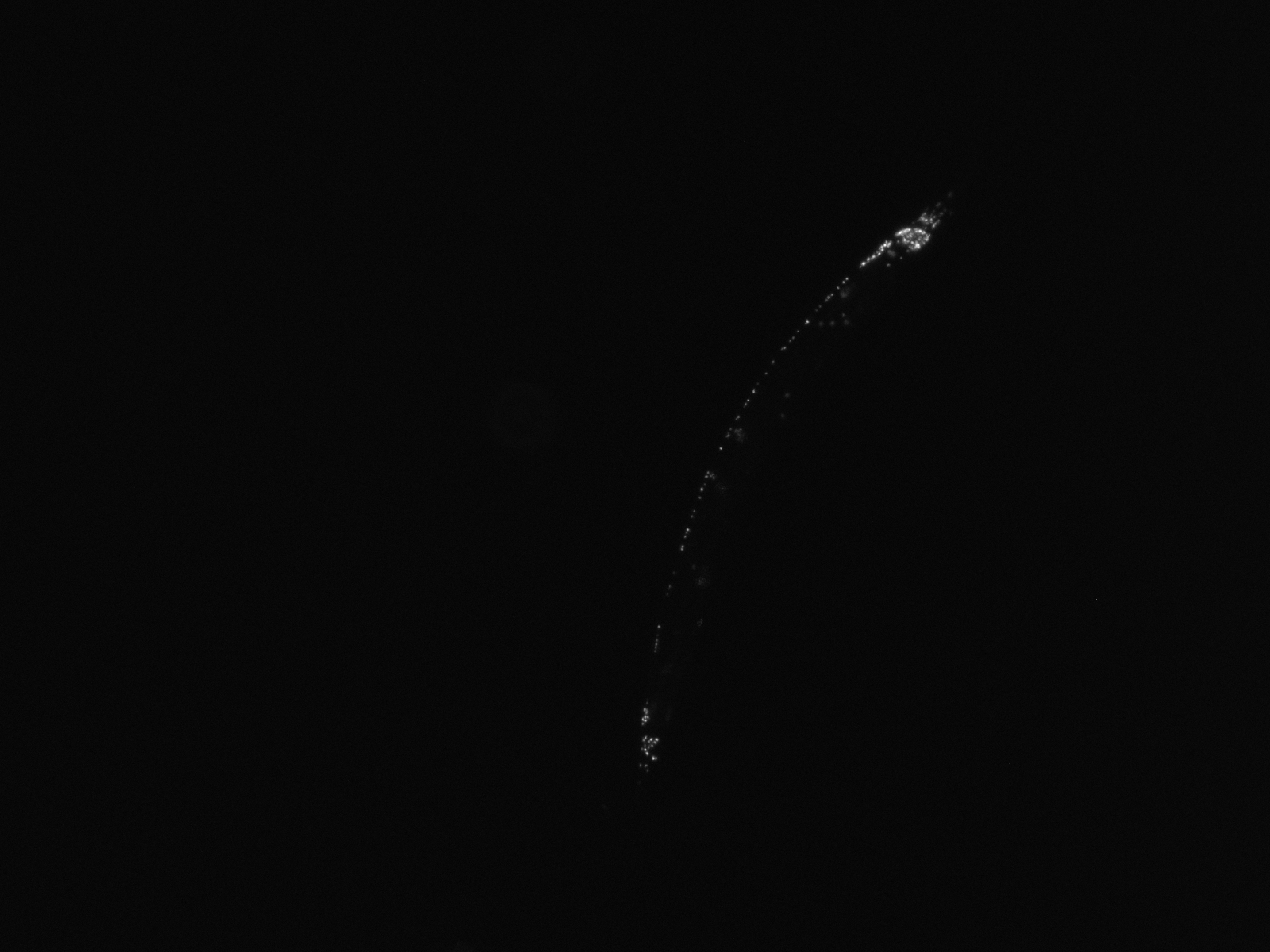

Supplement: Supplementary file 10 — Source Data [file 41467_2026_71615_MOESM10_ESM.zip › source data/microscopy_raw_images/FIGURE-6G/6G_RFP.tif]

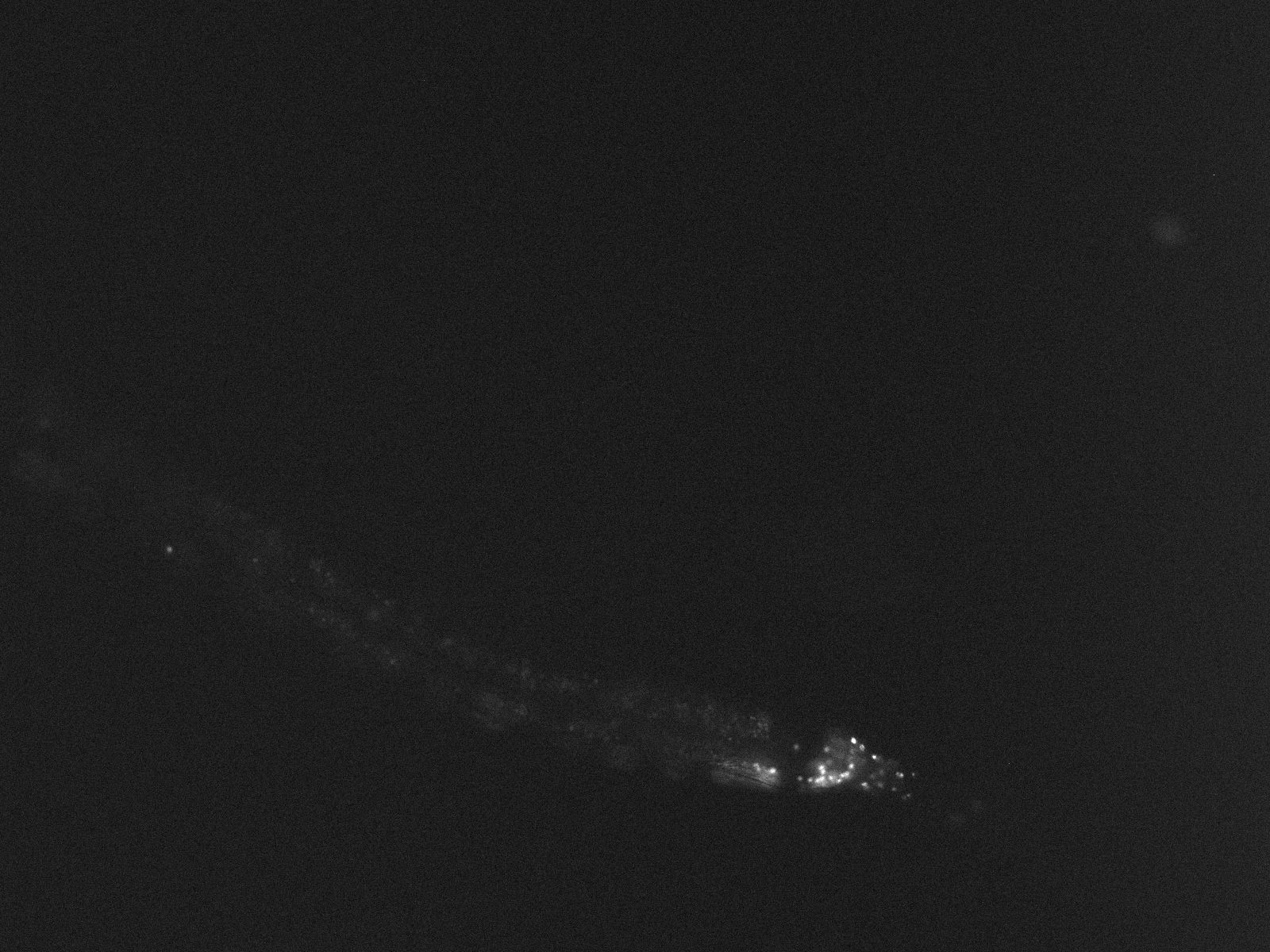

Supplement: Supplementary file 10 — Source Data [file 41467_2026_71615_MOESM10_ESM.zip › source data/microscopy_raw_images/FIGURE-6H/6H_RFP.tif]

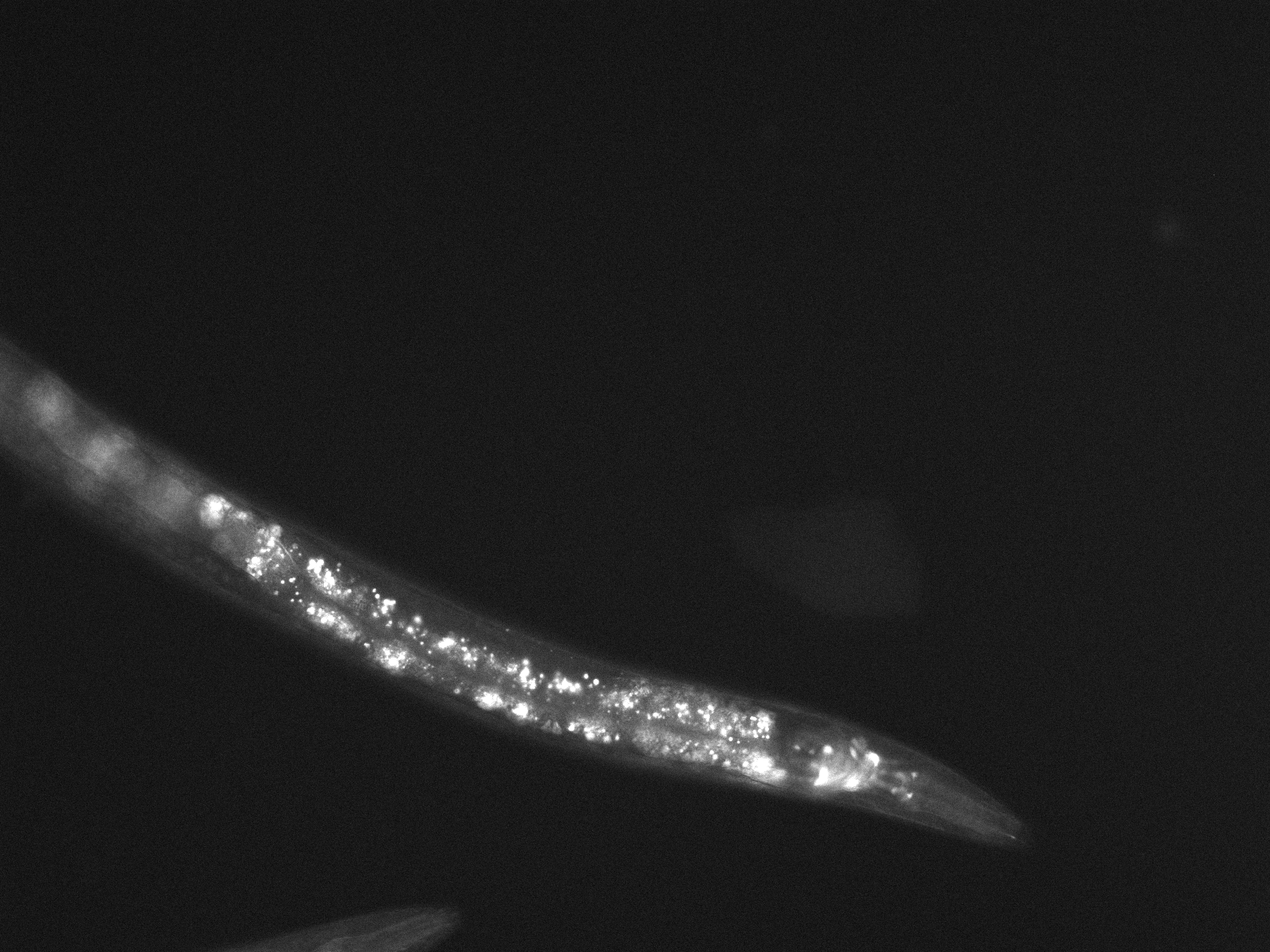

Supplement: Supplementary file 10 — Source Data [file 41467_2026_71615_MOESM10_ESM.zip › source data/microscopy_raw_images/FIGURE-6H/6H_GFP.tif]

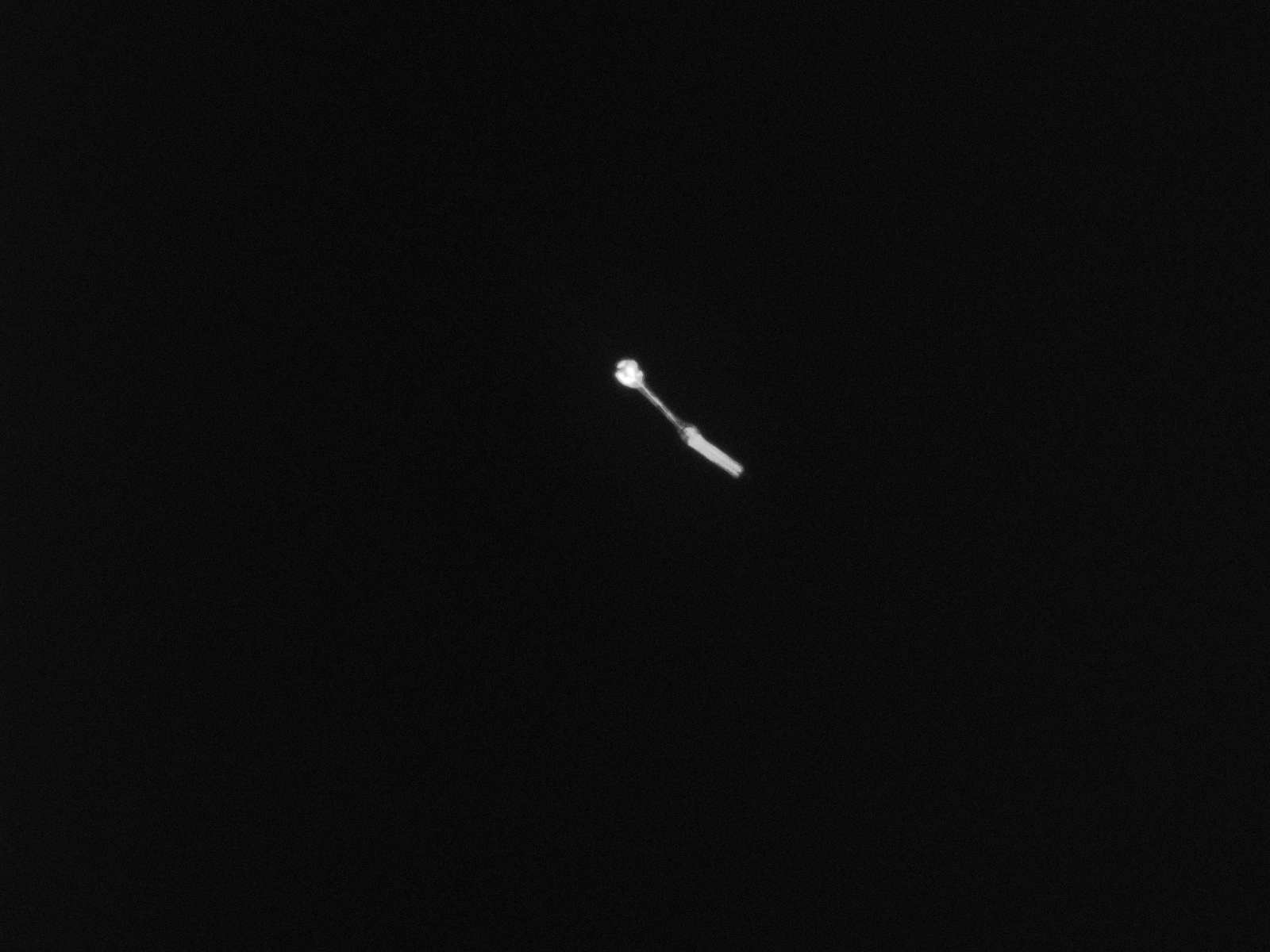

Supplement: Supplementary file 10 — Source Data [file 41467_2026_71615_MOESM10_ESM.zip › source data/microscopy_raw_images/SUPPLEMENT -S6D/tnt3_myo2_L1gfp.tif]

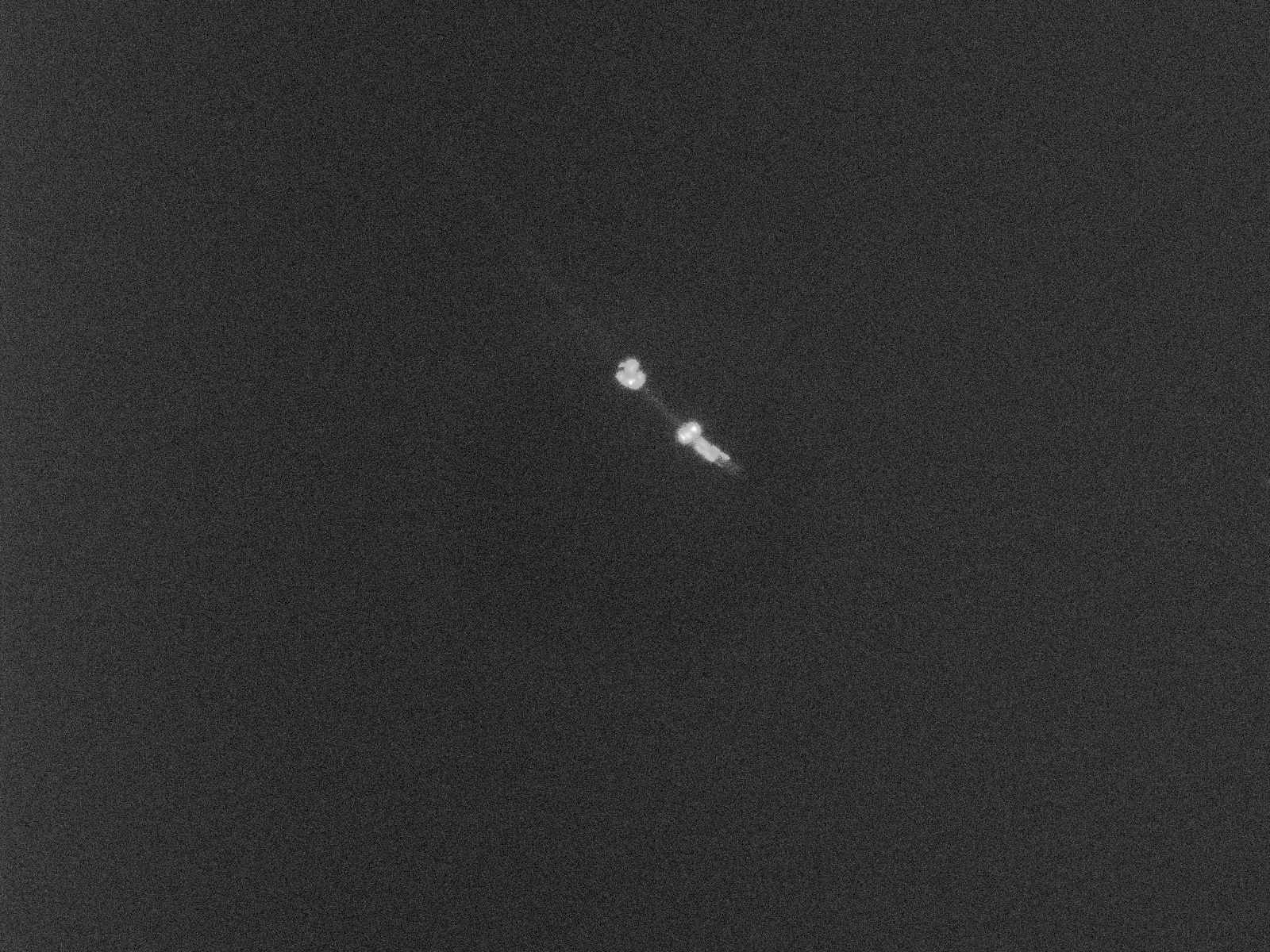

Supplement: Supplementary file 10 — Source Data [file 41467_2026_71615_MOESM10_ESM.zip › source data/microscopy_raw_images/SUPPLEMENT -S6D/tnt3_myo2_L1rfp.tif]

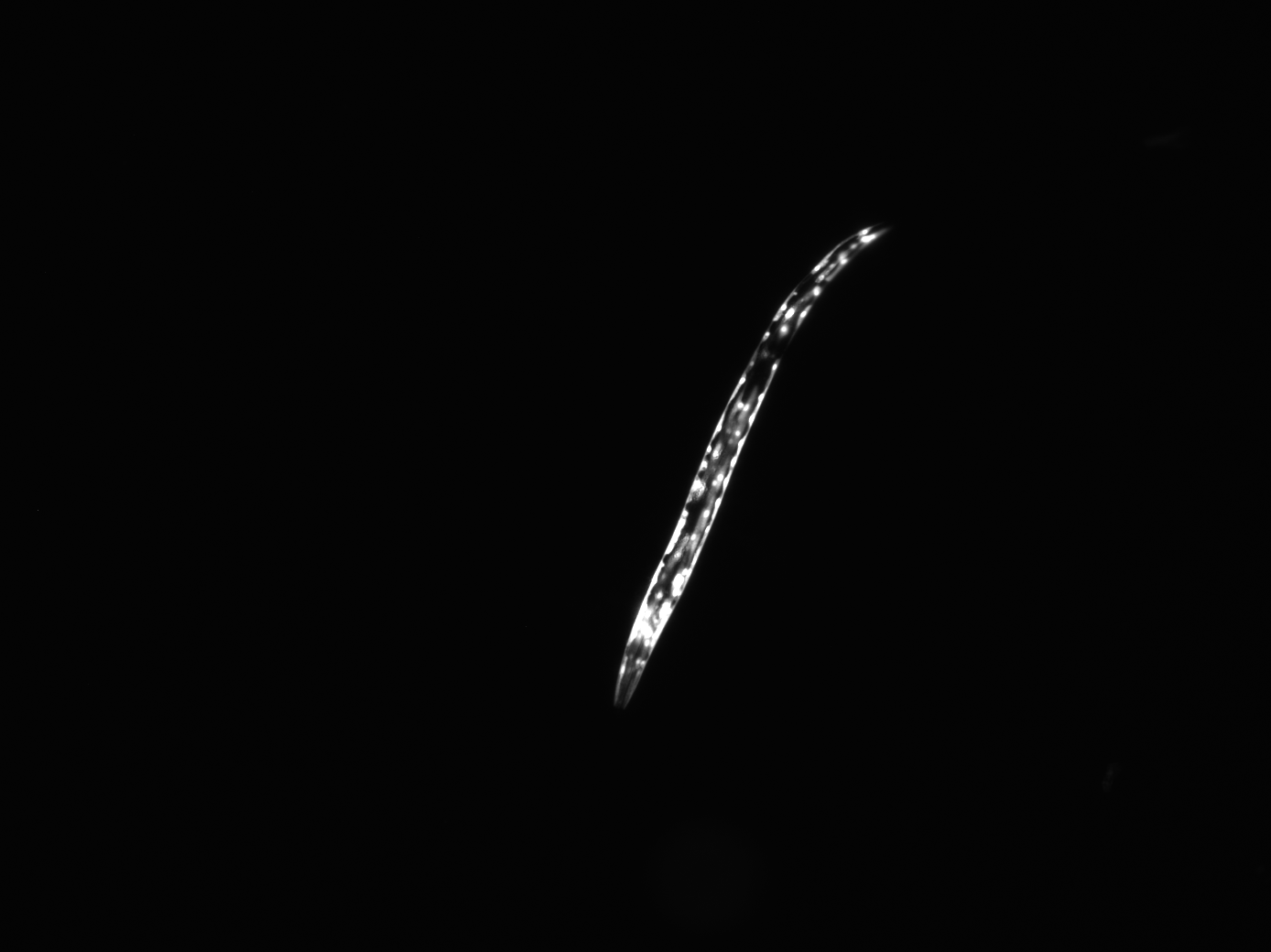

Supplement: Supplementary file 10 — Source Data [file 41467_2026_71615_MOESM10_ESM.zip › source data/microscopy_raw_images/SUPPLEMENT -S6E/tnt3_myo3_prp40_gfp.tif]

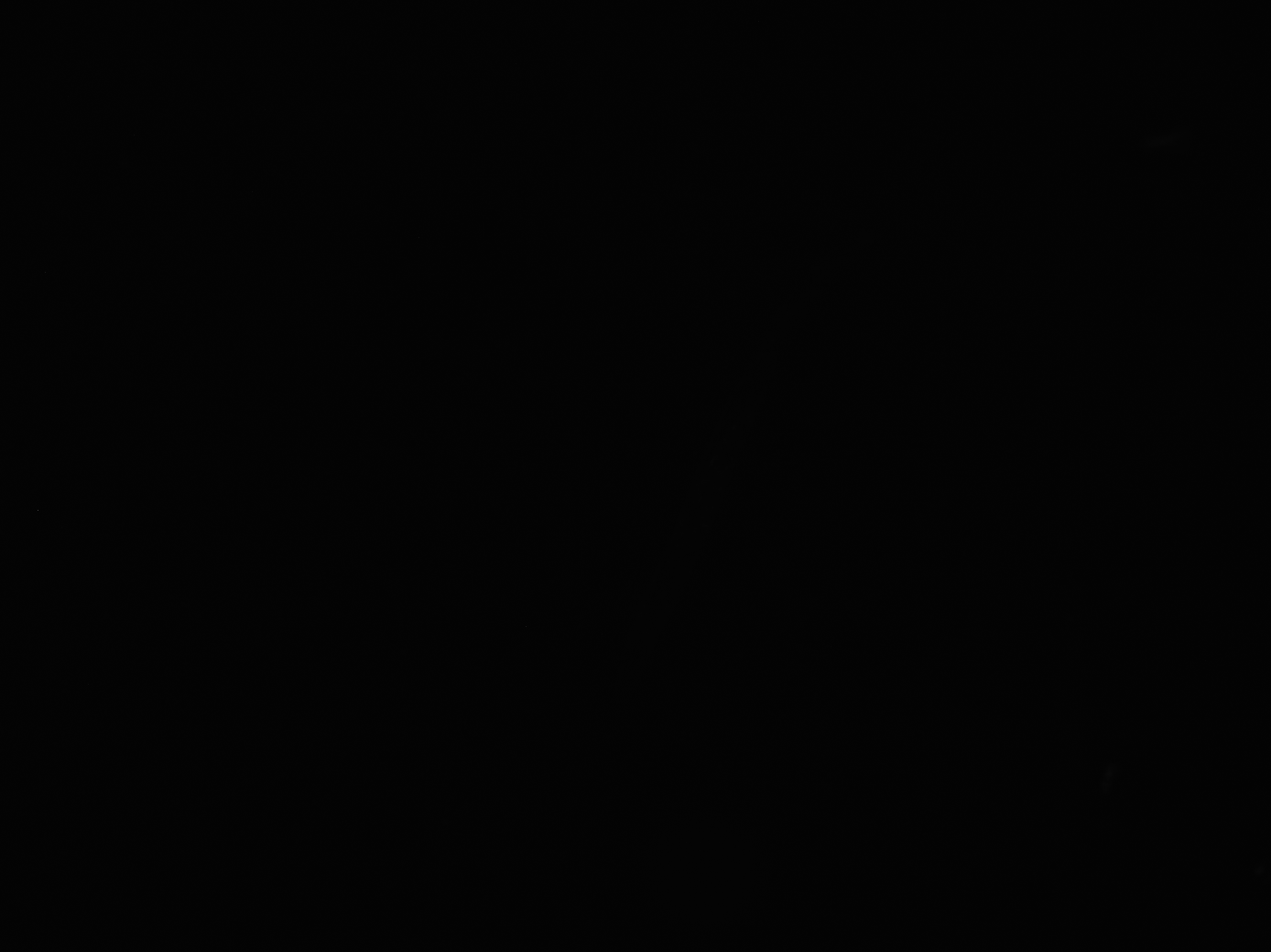

Supplement: Supplementary file 10 — Source Data [file 41467_2026_71615_MOESM10_ESM.zip › source data/microscopy_raw_images/SUPPLEMENT -S6E/tnt3_myo3_prp40_rfp.tif]

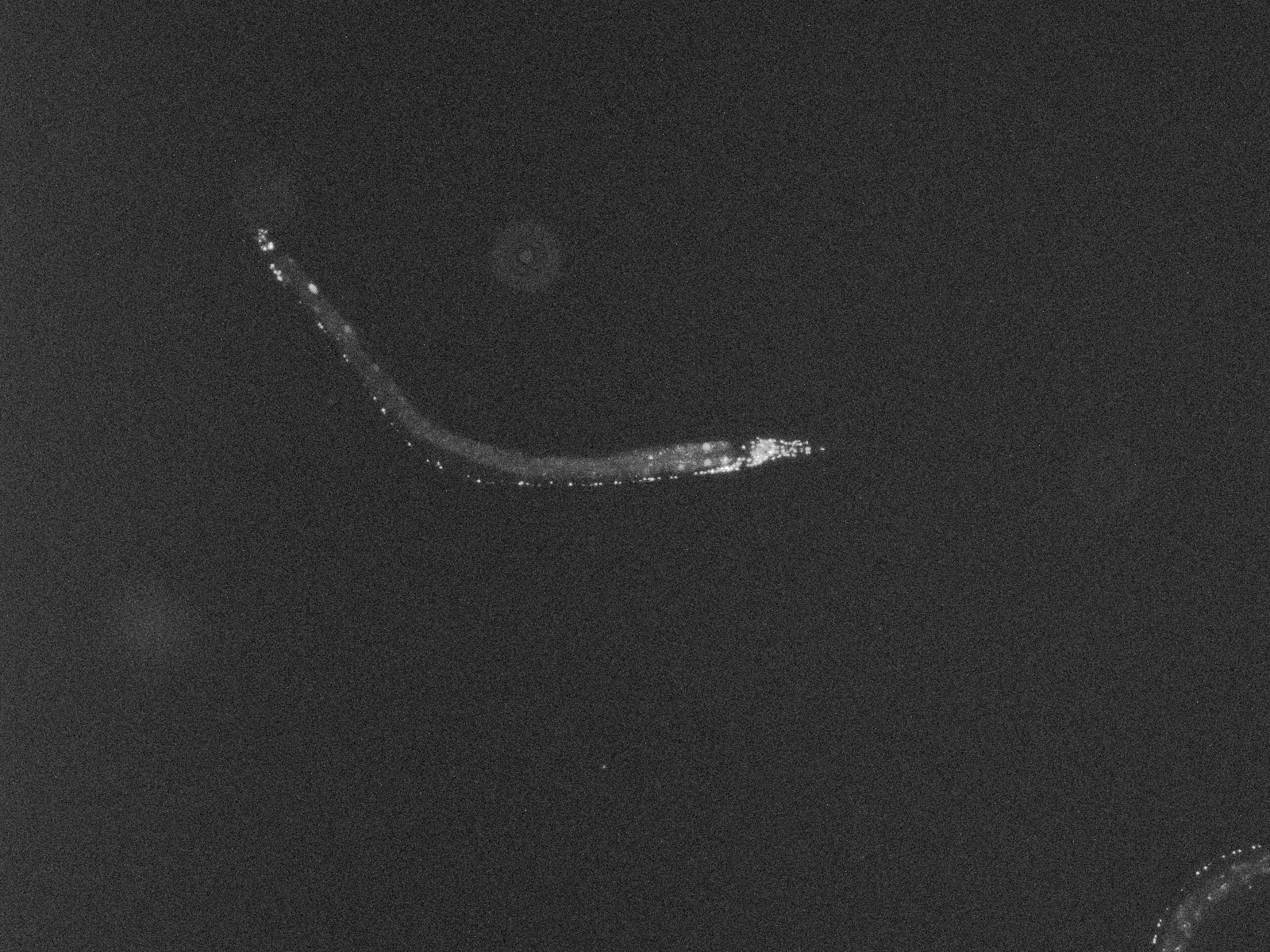

Supplement: Supplementary file 10 — Source Data [file 41467_2026_71615_MOESM10_ESM.zip › source data/microscopy_raw_images/FIGUTE-4J/fubl3_LOF_rfp.tif]

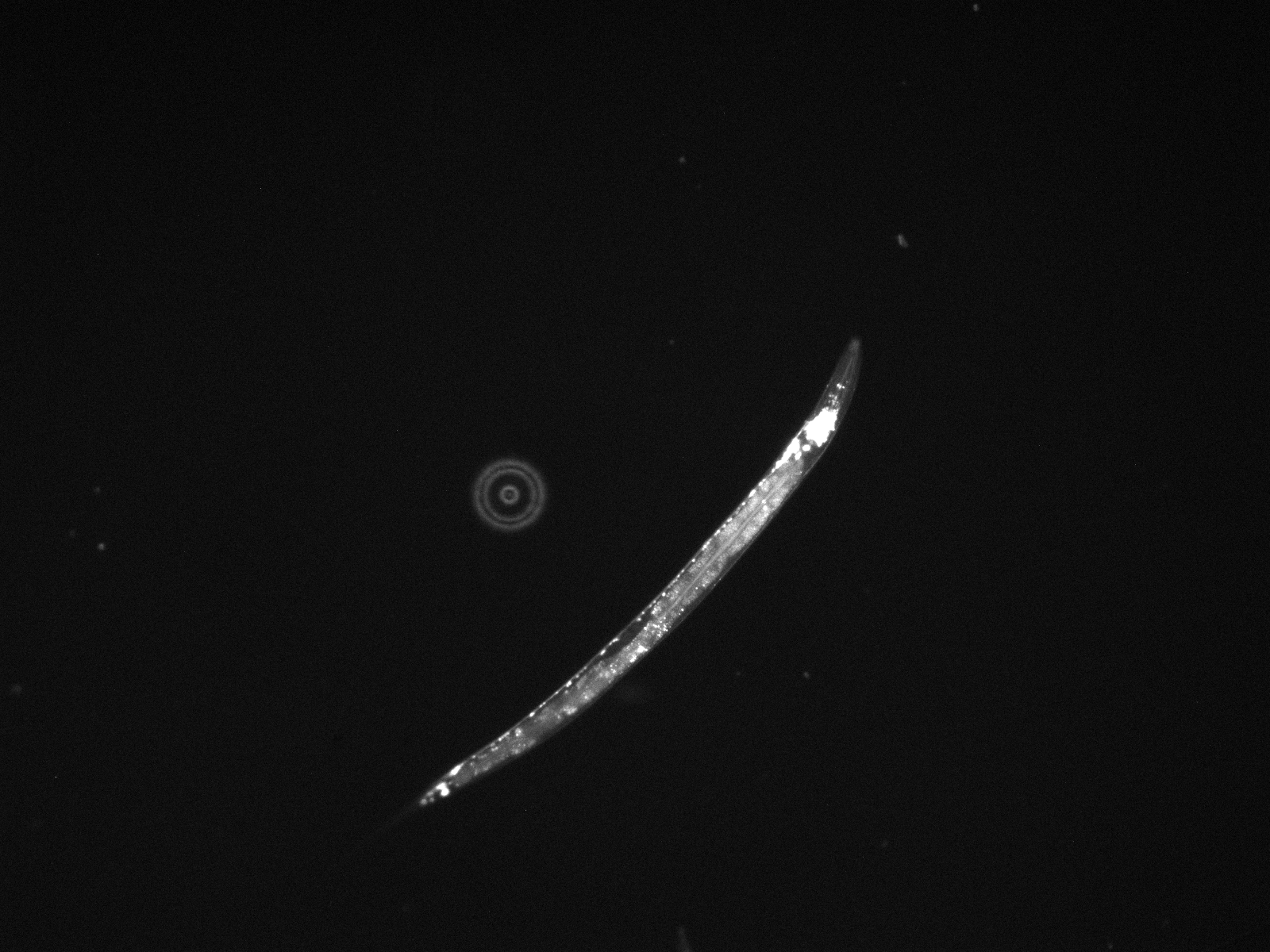

Supplement: Supplementary file 10 — Source Data [file 41467_2026_71615_MOESM10_ESM.zip › source data/microscopy_raw_images/FIGUTE-4J/fubl3_WT_gfp.tif]

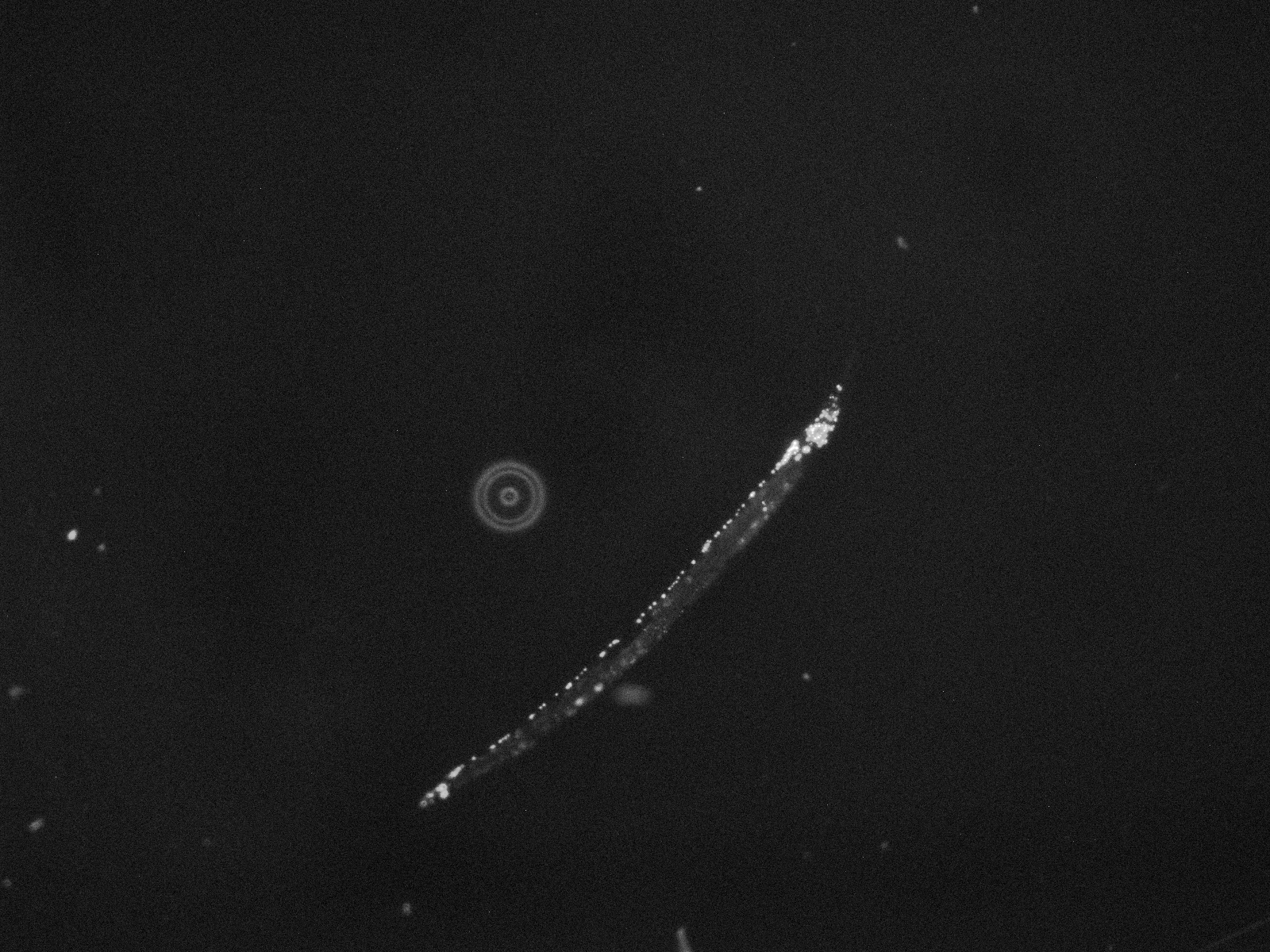

Supplement: Supplementary file 10 — Source Data [file 41467_2026_71615_MOESM10_ESM.zip › source data/microscopy_raw_images/FIGUTE-4J/fubl3_WT_rfp.tif]

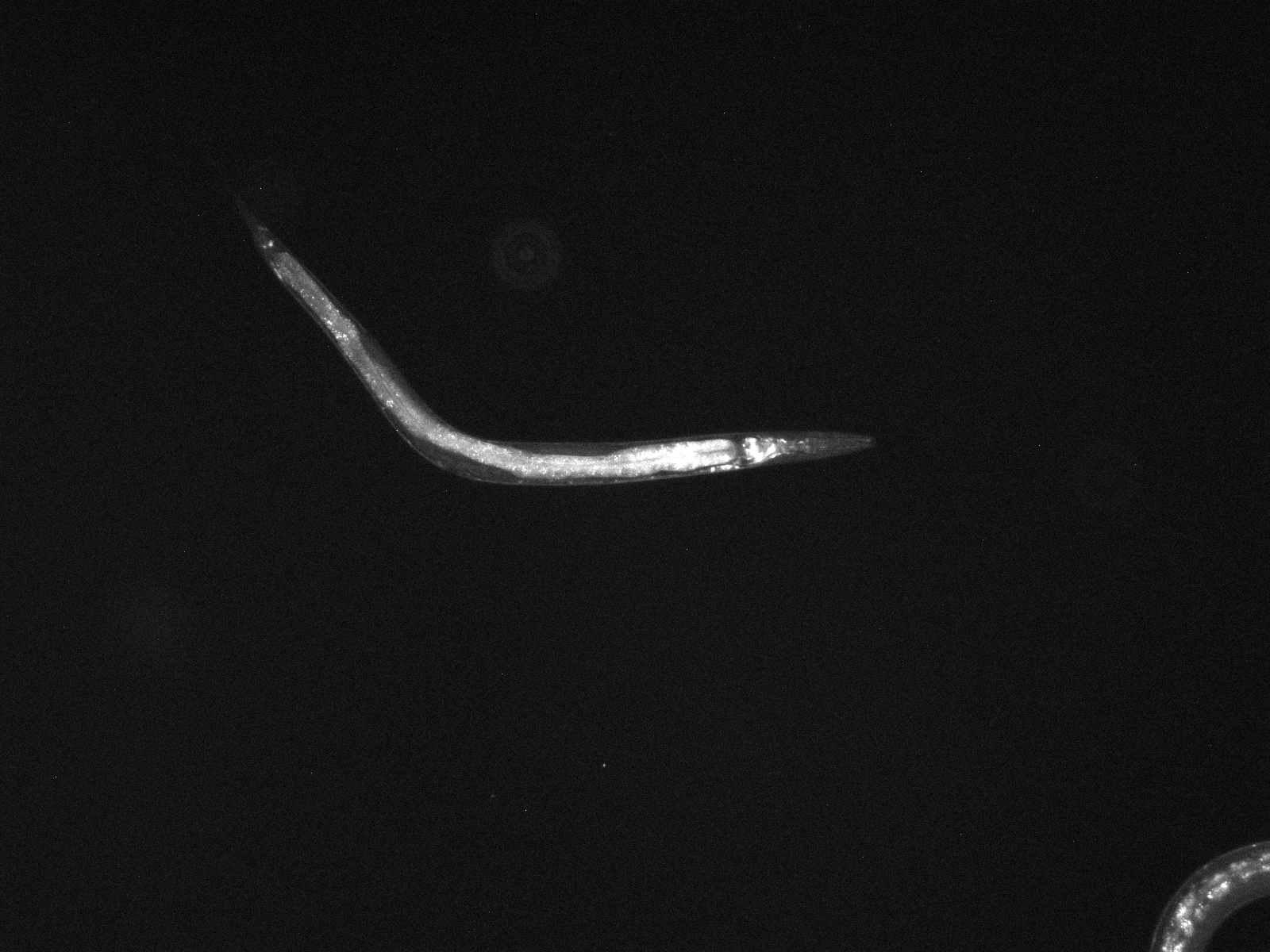

Supplement: Supplementary file 10 — Source Data [file 41467_2026_71615_MOESM10_ESM.zip › source data/microscopy_raw_images/FIGUTE-4J/fubl3_LOF_gfp.tif]

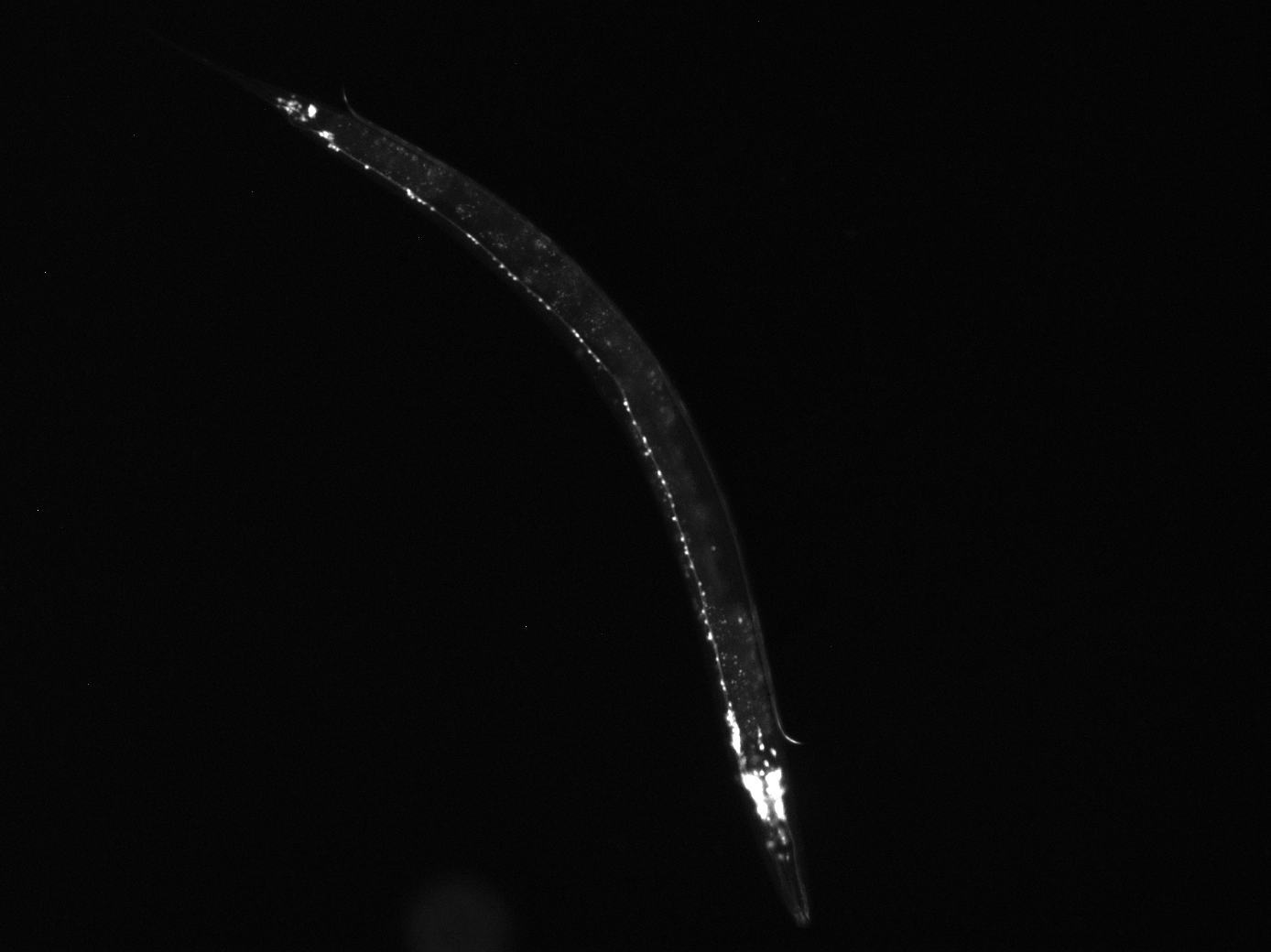

Supplement: Supplementary file 10 — Source Data [file 41467_2026_71615_MOESM10_ESM.zip › source data/microscopy_raw_images/FIGURE-4I/fubl1_wt_gfp.tif]

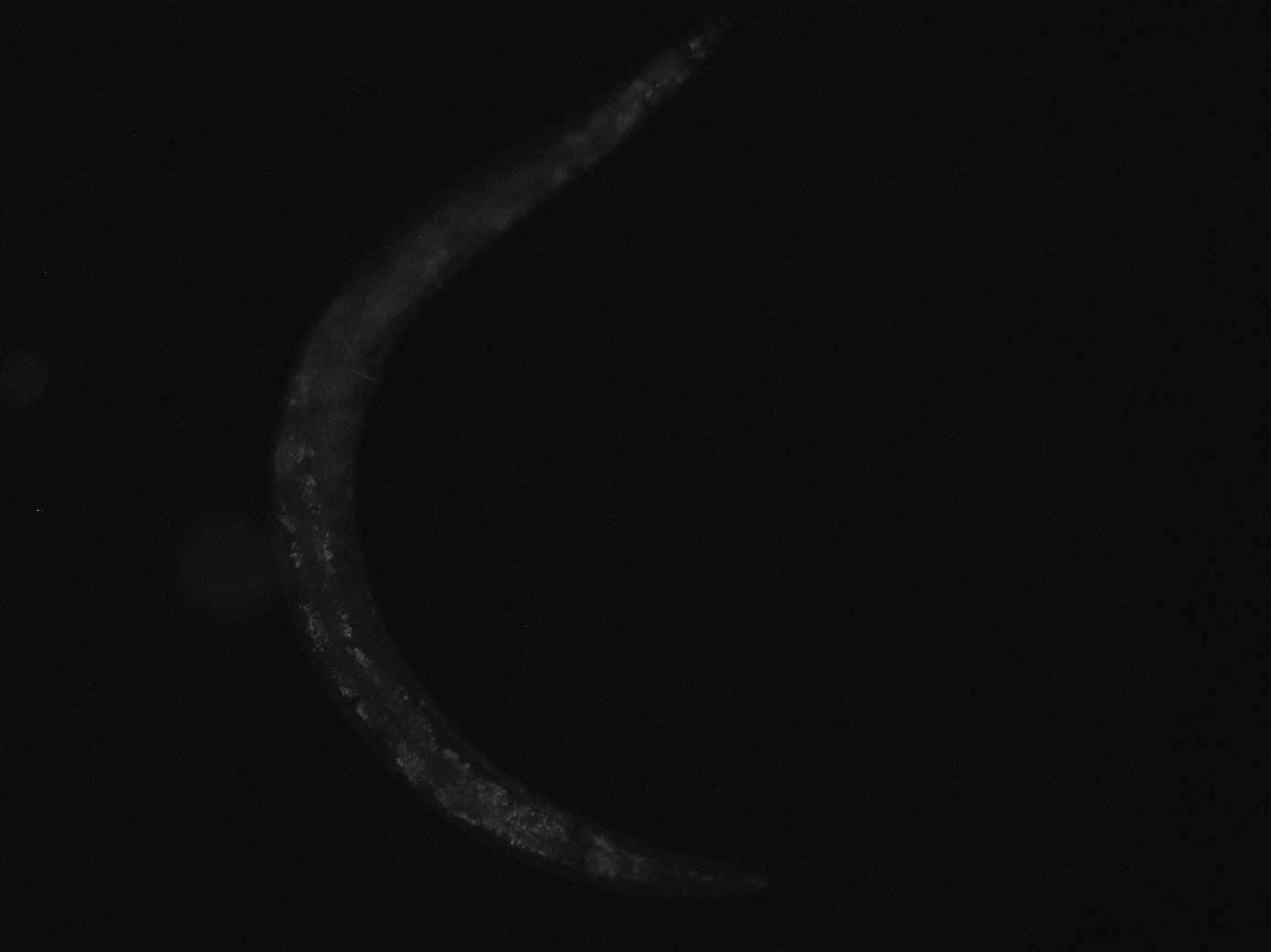

Supplement: Supplementary file 10 — Source Data [file 41467_2026_71615_MOESM10_ESM.zip › source data/microscopy_raw_images/FIGURE-4I/fubl1_lof_gfp.tif]

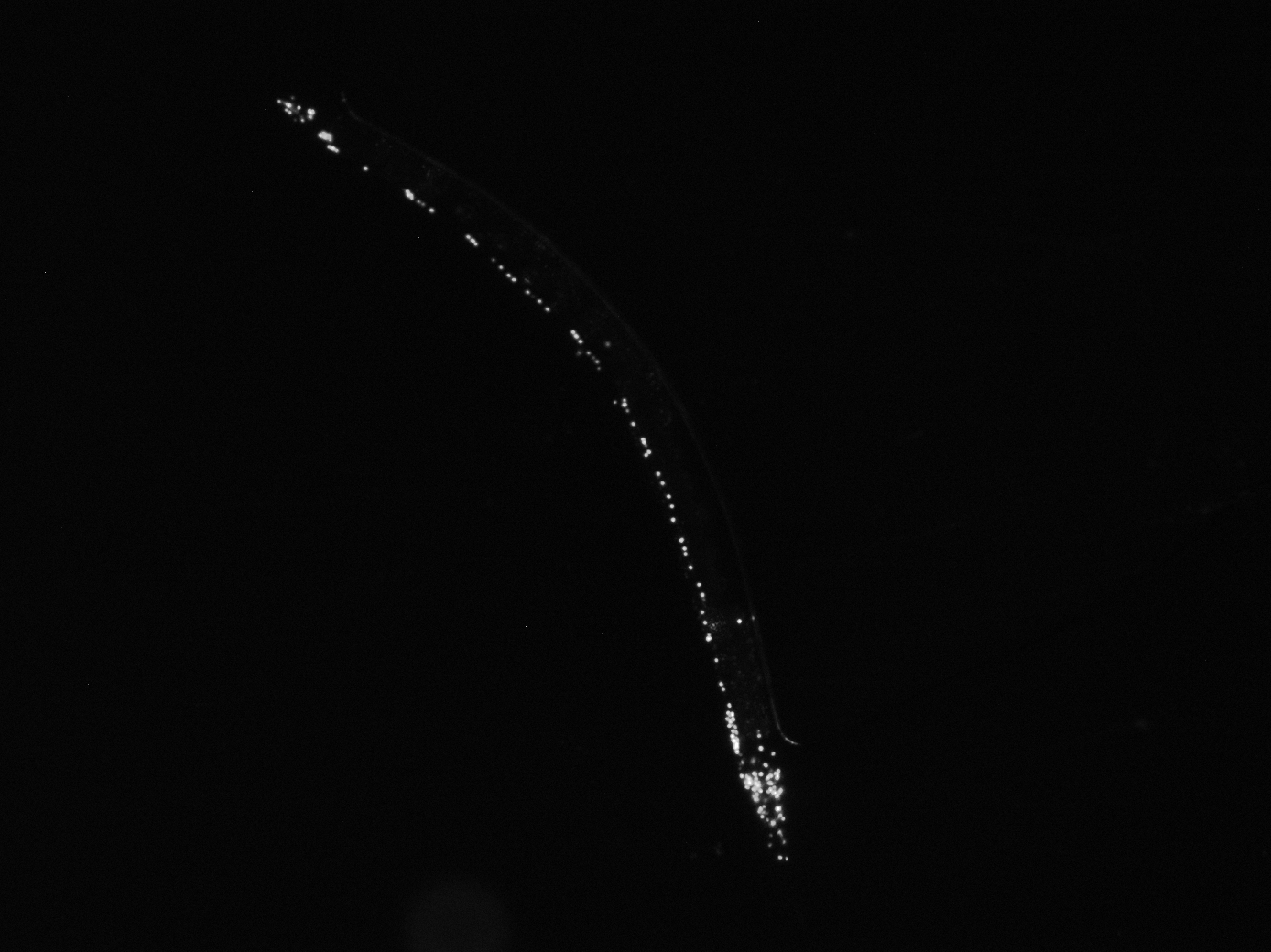

Supplement: Supplementary file 10 — Source Data [file 41467_2026_71615_MOESM10_ESM.zip › source data/microscopy_raw_images/FIGURE-4I/fubl1_wt_rfp.tif]

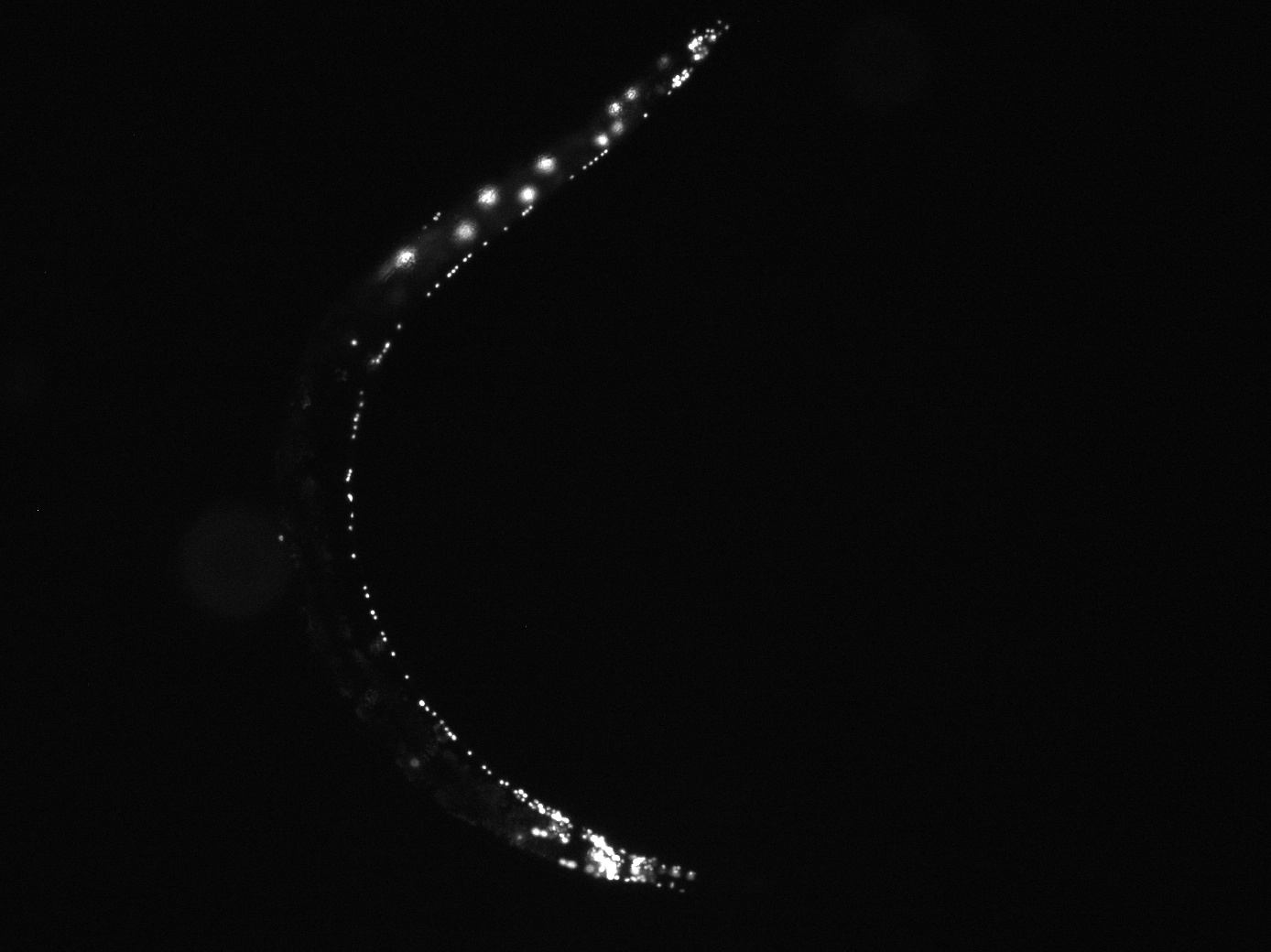

Supplement: Supplementary file 10 — Source Data [file 41467_2026_71615_MOESM10_ESM.zip › source data/microscopy_raw_images/FIGURE-4I/fubl1_lof_rfp.tif]

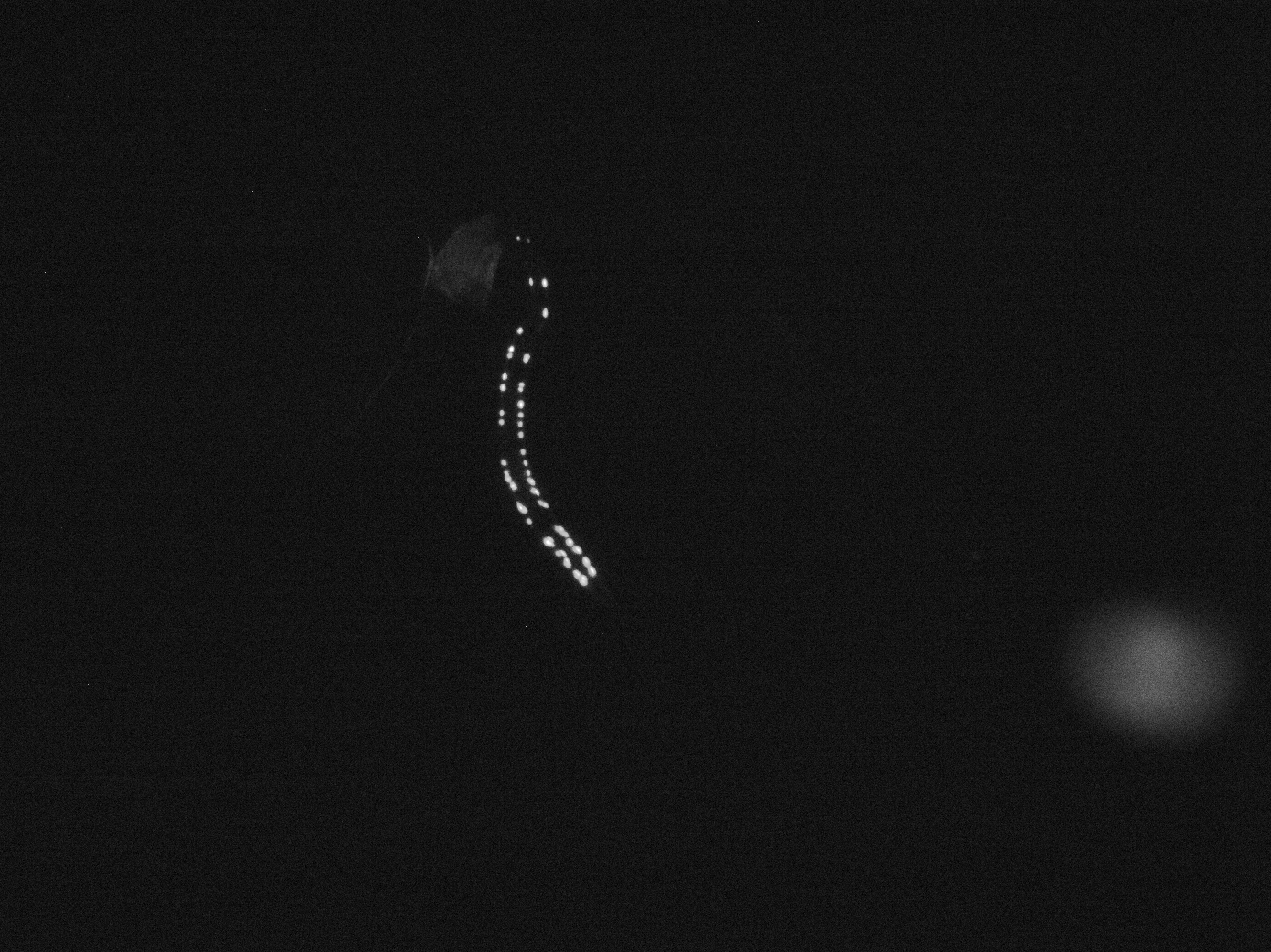

Supplement: Supplementary file 10 — Source Data [file 41467_2026_71615_MOESM10_ESM.zip › source data/microscopy_raw_images/FIGURE-7H/tnt3_myo3_rfp.tif]

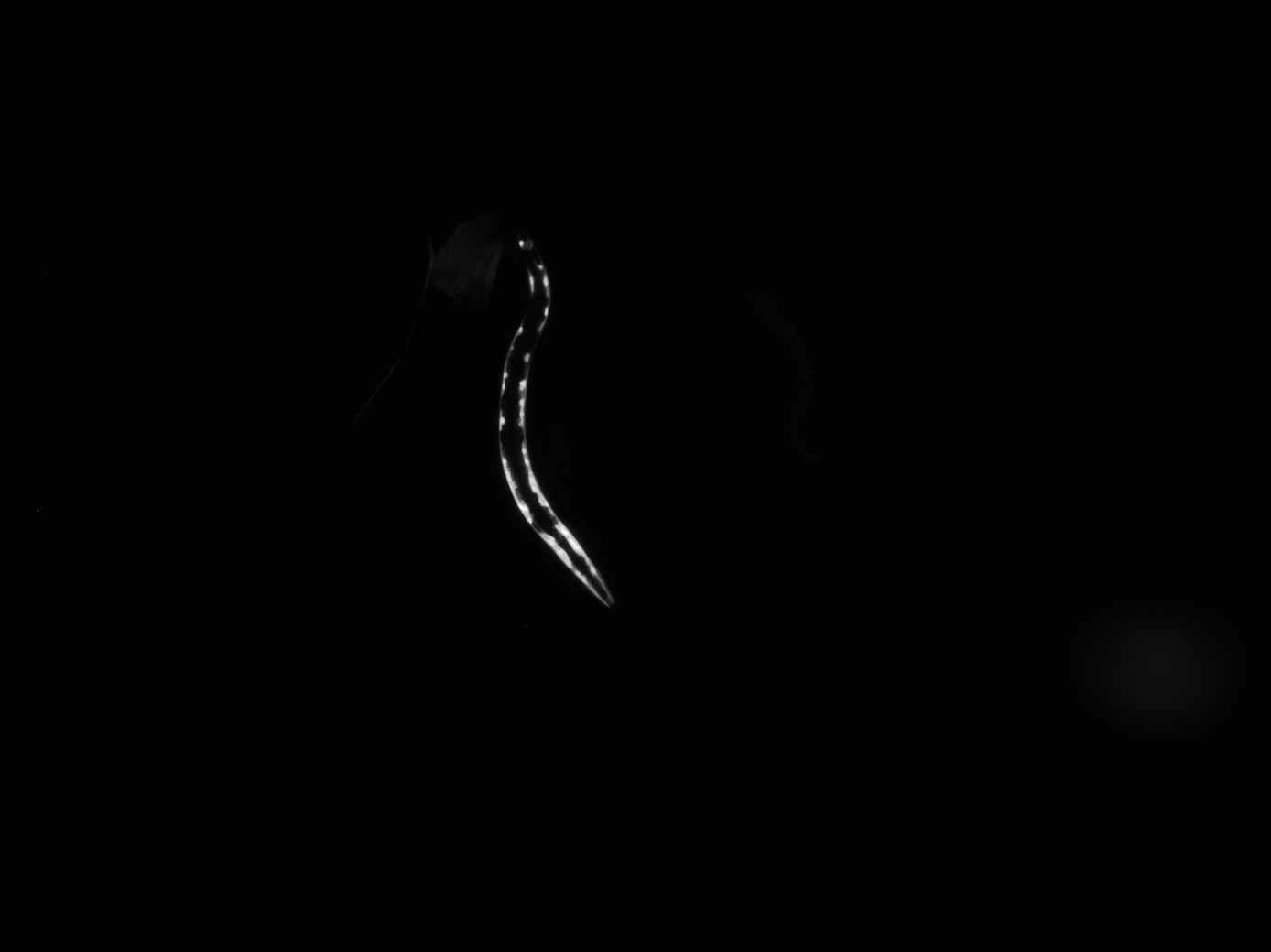

Supplement: Supplementary file 10 — Source Data [file 41467_2026_71615_MOESM10_ESM.zip › source data/microscopy_raw_images/FIGURE-7H/tnt3_myo3_gfp.tif]
