# Supplementary material for: Pervasive non-triplet alternative splicing drives functional isoform diversity
Source: Nat Commun. 2026 Apr 10;17:5112. doi: 10.1038/s41467-026-71615-5 (PMC13247117; doi:10.1038/s41467-026-71615-5)
Supplement: Supplementary file 9 — Transparent Peer Review file [file 41467_2026_71615_MOESM9_ESM.pdf]

nature portfolio

Peer Review File

Pervasive non-triplet alternative splicing drives functional isoform diversity

Corresponding Author: Dr Adam Norris

This file contains all reviewer reports in order by version, followed by all author rebuttals in order by version.

Version 0:

Reviewer comments:

Reviewer #1

(Remarks to the Author)
In their manuscript, the authors investigate non-triplet forms of alternative splicing in C.elegans and its potential consequences on both gene expression levels and isoform-specific functions. Their work address a long-known but still relatively understudied question: what is the overall prevalence and functional relevance of non-triplet splicing? While this is most often associated with NMD and described as a means of regulating expression levels, the authors find that in addition to this category, there are also many examples in which splicing of a non-triplet exon can recode significant regions of the expressed protein. This work also serves to illustrate that the steady-state levels of different splicing outcomes can be heavily affected by NMD activity (ie. unproductive NMD-sensitive splicing can be much more prevalent than anticipated) and that isoform-specific expression can have distinct and unexpected phenotypes as compared to whole-gene knockouts (as they demonstrate in the fubl-1 experiments). Overall, this study is well done and in particular, the authors do a nice job of taking the results of their global analyses and functionally validating specific examples in each of their described categories of non-triplet splicing outcomes (ie. NMD-sensitive and NMD-resistant isoform-altering).

Some specific comments/questions are as follows:
1. For the analysis of dual-coding isoforms, the authors state "We set a threshold of 60 recoded nucleotides to qualify as dual coding, reasoning that this is a sufficient number of codons to encode a small protein domain (Ref 38,39)." While I agree that this is a reasonable threshold, have the authors performed any analyses to examine this? Specifically, how does the number of dual coding events change with the length of this threshold? Is there any bias in the distribution of the change in coding length between isoforms?
2. In Fig 3F/G, an important caveat is that the mutants of Fubl-1 or 3 may also be affecting expression levels and/or splicing of the RNA in unanticipated ways (ie. not necessarily via loss of the autoregulation -- although this possibility is somewhat addressed by the reporter experiments of panel H/I/J). What are the actual mutants used in these experiments? And what is the overall expression of Fubl-1 and Fubl-3 in their respective mutants as compared to wildtype? Is it possible that the mutants have lower expression which could be affecting the sensitivity of detecting both isoforms by RT-PCR? I am specifically wondering about this for Fubl-1 (Fig 3F), where the excluded band is already faint in the WT.
3. For the Fig 7 human transcriptome data, it would be informative to provide some of same additional analyses as was done for the C.elegans data. For example, as in Fig 2B/C, what is breakdown between essential vs poison exons in human? And as was done in Fig 2J, for the upregulated/downregulated non-triplet splicing events in human, what is the gene ontology enrichment look like?
4. I believe the ref 50 Wollerton et al PTBP1 paper (PMID: 14731397) may be mis-cited here. The authors reference it stating: "This parallels previous reports of poison cassette exons enriched in splicing factor genes (ref 23,50), indicating that non-triplet poison/essential splicing affects similar gene networks to those of traditional poison cassette exons." However, my understanding of this paper is that it does not describe a poison exon and instead represents an example of RBP autoregulation via promoting skipping of an essential exon (much like their Fubp-1/3 example). Another relevant example is described in Zhou et al about FUS (PMID:24204307).

(Remarks on code availability)

Reviewer #2

(Remarks to the Author)
In this study, Athavudeen and colleagues investigate non-triplet alternative splicing in NMD-deficient worms. They identify and catalog hundreds of non-triplet splicing events that result in one of three functional outcomes: NMD-mediated depletion, alternative C-terminal length, or dual-coding potential. Prior studies of non-triplet splicing have primarily focused on their propensity to trigger NMD, with a few studies identifying homeostatic regulatory roles for such splicing events. This study highlights additional consequences that have thus far been underappreciated. The key strength of this study is its demonstration of physiological significance for several examples of non-triplet alternative splicing, taking advantage of the in vivo model system. This is an important contribution to the literature and highlights the regulatory potential of non-triplet splicing beyond its role in homeostatic dosage control.

A major concern I have with this study is its reliance solely on the bioinformatics pipeline, JUM, to discover and catalog splicing events. JUM has known limitations, especially in providing inaccurate coordinates of splicing events (see the benchmarking study, Jiang et al. 2023). For a study that requires accurate determination of reading frame and sequence coordinates, it would be important to demonstrate that more robust pipelines such as rMATS or Leafcutter (for annotation-free analysis) produce the same/similar results.

Some minor points to address or suggestions to improve the study are as follows:
1. Have the authors looked at the sequence conservation of specific non-triplet splicing events, especially those with physiological relevance?
2. Are the alternative dual coding regions introduced by non-triplet splicing conserved in the same way as the main reading frame, specifically when considering the wobble position of codons?
3. I recommend systematically reviewing all axis labels and legends to ensure that all text is legible and consistently formatted.

(Remarks on code availability)

Reviewer #3

(Remarks to the Author)
This manuscript analyzes non-triplet alternative splicing in C. elegans. Importantly, a key innovation is using NMD-defective mutants. This allos disambiguation of splicing from NMD. Many novel instances of non-triplet splicing are identified. Further analysis classifies these into several distinct categories—NMD-sensitive, potein truncation, and isoform sw2itching. In vivo experiments are then performed to validate the findings and investigate the function of specific non-triplet splice events.
The manuscript presents a unique and important data set, combined with deep analysis and validation. It will be of interest to many readers. I am strongly in favor of publication essentially as is. I have only one suggestion for revision:
Using the smg-1 and smg-2 mutants is key to identify which splice forms are subject to NMD, and to disambiguate splicing and NMD. But the explanation of how these data were analyzed is lacking. Moving parts of Sup fig 1-3 into a main figure devoted to this analysis would improve the readability. It would be helpful to walk through the analysis, explaining how transcripts were identified as NMD-sensitive, to what extent smg-1 and smg-2 gave similar results, etc. This analysis could be figure 1, since these results are implicit in the existing fig 1 and onward.

(Remarks on code availability)

Version 1:

Reviewer comments:

Reviewer #1

(Remarks to the Author)
I thank the authors for addressing my comments and am satisfied with the revisions made.

(Remarks on code availability)

Reviewer #2

(Remarks to the Author)
The authors have sufficiently addressed all of my concerns.

(Remarks on code availability)

Open Access This Peer Review File is licensed under a Creative Commons Attribution 4.0 International License, which permits use, sharing, adaptation, distribution and reproduction in any medium or format, as long as you give appropriate credit to the original author(s) and the source, provide a link to the Creative Commons license, and indicate if changes were made.
In cases where reviewers are anonymous, credit should be given to 'Anonymous Referee' and the source.
The images or other third party material in this Peer Review File are included in the article's Creative Commons license, unless indicated otherwise in a credit line to the material. If material is not included in the article's Creative Commons license and your intended use is not permitted by statutory regulation or exceeds the permitted use, you will need to obtain permission directly from the copyright holder.
To view a copy of this license, visit https://creativecommons.org/licenses/by/4.0/

We thank the reviewers for their constructive suggestions. We believe we have successfully performed all the requested analyses, experiments, and modifications, as detailed below, and that the manuscript has improved in clarity and thoroughness. Modified text is highlighted in the manuscript by blue text color.

Reviewer #1

1. For the analysis of dual-coding isoforms, the authors state "We set a threshold of 60 recoded nucleotides to qualify as dual coding, reasoning that this is a sufficient number of codons to encode a small protein domain (Ref 38,39)." While I agree that this is a reasonable threshold, have the authors performed any analyses to examine this? Specifically, how does the number of dual coding events change with the length of this threshold? Is there any bias in the distribution of the change in coding length between isoforms?

We have now explored a range of cutoffs to qualify as dual coding, testing how this affects the ratio of 'alternative C terminal isoforms' versus 'dual coding isoforms,' as well as the length distribution of dual coding regions, and report the results in lines 135-137 and figure S2B and S2C. The impact of the threshold choice is in the expected direction, *e.g.* decreasing the threshold by two-fold results in a nearly two-fold increase in dual coding events at the expense of the alternative C-terminal isoforms category (figure S2B), but these changes occur without substantially impacting the coding-length-difference distribution (figure S2C).

2. In Fig 3F/G, an important caveat is that the mutants of Fubl-1 or 3 may also be affecting expression levels and/or splicing of the RNA in unanticipated ways (ie. not necessarily via loss of the autoregulation -- although this possibility is somewhat addressed by the reporter experiments of panel H/I/J). What are the actual mutants used in these experiments? And what is the overall expression of Fubl-1 and Fubl-3 in their respective mutants as compared to wildtype? Is it possible that the mutants have lower expression which could be affecting the sensitivity of detecting both isoforms by RT-PCR? I am specifically wondering about this for Fubl-1 (Fig 3F), where the excluded band is already faint in the WT.

To address the question of whether fubl-1 and fubl-3 transcript levels are affected in the respective loss-of-function mutants, which might hinder our ability to detect the minor isoform, we have now performed qPCR in these mutants (figure S4A-B). We find that the mutants do not cause a significant change in their respective transcripts, which we believe addresses the concern of decreased transcript abundance as a cause for decreased isoform detection. We also now clarify the molecular nature of the loss-of-function fubl-1 and fubl-3 mutants as small deletions that are located distally and downstream of the autoregulatory alternative splicing events (lines 595-598). We also now highlight (lines 263-266) that, as pointed out by the reviewer, the splicing reporters further support the trans-acting nature of the loss-of-function mutants, since the transgenic splicing reporters are driven by an orthogonal promoter and should therefore not be affected in *cis* by the loss-of-function mutations.

3. For the Fig 7 human transcriptome data, it would be informative to provide some of same additional analyses as was done for the C.elegans data. For example, as in Fig 2B/C, what is breakdown between essential vs poison exons in human? And as was done in Fig 2J, for the upregulated/downregulated non-triplet splicing events in human, what is the gene ontology enrichment look like?

We have now included additional human non-triplet analyses similar to those done for the C. elegans non-triplets, such as poison versus essential exons, number of exons recoded, and gene ontology categories (lines 504-515, figure 8E-G, figure SC-E). The general contours of the results are the same across species, though in most cases the total number of events is substantially higher in humans than in C. elegans.

4. I believe the ref 50 Wollerton et al PTBP1 paper (PMID: 14731397) may be mis-cited here. The authors reference it stating: "This parallels previous reports of poison cassette exons enriched in splicing factor genes (ref 23,50), indicating that non-triplet poison/essential splicing affects similar gene networks to those of traditional poison cassette exons." However, my understanding of this paper is that it does not describe a poison exon and instead represents an example of RBP autoregulation via promoting skipping of an essential exon (much like their Fubp-1/3 example). Another relevant example is described in Zhou et al about FUS (PMID:24204307).

Thank you for bringing this to our attention. We have now adopted the correct citations (line 219).

Reviewer #2

A major concern I have with this study is its reliance solely on the bioinformatics pipeline, JUM, to discover and catalog splicing events. JUM has known limitations, especially in providing inaccurate coordinates of splicing events (see the benchmarking study, Jiang et al. 2023). For a study that requires accurate determination of reading frame and sequence coordinates, it would be important to demonstrate that more robust pipelines such as rMATS or Leafcutter (for annotation-free analysis) produce the same/similar results.

We have now re-analyzed our data using identical pipelines except in place of JUM we employed a more widely-used alternative splicing tool (MAJIQ: Modeling Alternative Junction Inclusion Quantification) to perform the alternative splicing analysis. The comparisons are displayed in lines 95-97, figure S1A-C, and figure 2A, figure S2A, which show that the alternative splicing detection is highly concordant between the MAJIQ-based and the JUM-based pipelines. Most importantly for our purposes, the final proportions of non-triplet isoform types (NMD, alternative C-terminus, and dual coding) are similar between both methods (cf. figure S1C, figure 2A).

Some minor points to address or suggestions to improve the study are as follows:
1. Have the authors looked at the sequence conservation of specific non-triplet splicing events, especially those with physiological relevance?

We have now performed this analysis, plotting conservation (phyloP) scores for each category of alternative splicing (NMD, C-terminus, dual-coding) and compared both triplet non-triplet alternative exons. The general trends are that conservation scores are the same across different categories as well as between triplet and non-triplet events within a category (lines 139-141, figure S2D). We also highlight the splicing events we examined with physiological relevance (line 435-436, figure S2D), some of which (tnt-3, fubl-1) are above-average in conservation, others (fubl-3, dhhc-4) slightly lower than average.

2. Are the alternative dual coding regions introduced by non-triplet splicing conserved in the same way as the main reading frame, specifically when considering the wobble position of codons?

We addressed this question by plotting conservation scores for the dual-coding versus the non-dual-coding (single-coding) regions for all genes that encode dual-coding regions. Interestingly, while positions 1 and 2 of the canonical reading frame are slightly less conserved than those of the single-coding regions, position 3 is significantly more conserved in the dual-coding regions than in the single-coding regions (figure 7C, lines 421-423). This is consistent with the idea that both reading frames in dual-coding regions are under evolutionary selection.

3. I recommend systematically reviewing all axis labels and legends to ensure that all text is legible and consistently formatted.

In this revised manuscript we have reviewed all figure panels and tried to increase font sizes, uniformity of text font, and uniformity of text color throughout the manuscript.

Reviewer #3

Using the smg-1 and smg-2 mutants is key to identify which splice forms are subject to NMD, and to disambiguate splicing and NMD. But the explanation of how these data were analyzed is lacking. Moving parts of Sup fig 1-3 into a main figure devoted to this analysis would improve the readability. It would be helpful to walk through the analysis, explaining how transcripts were identified as NMD-sensitive, to what extent smg-1 and smg-2 gave similar results, etc. This analysis could be figure 1, since these results are implicit in the existing fig 1 and onward.

We have now enacted this suggestion: a new figure 1 now presents an explanation of how the RNA-Seq data were generated, how alternative splicing was called, and highlights the comparisons between smg-1 and smg-2 data, using a number of panels that were previously in supplemental figures 1-3. Furthermore, in new figure 2 (formerly figure 1), we add further details about the parameters governing Nonsense Mediated Decay identification as well as the categorization into the three molecular outcomes of non-triplet alternative splicing. We hope this will provide a clearer entry-point to understanding how our findings were obtained.
